# Estimation of Copy Number Alterations from Exome Sequencing Data

**DOI:** 10.1371/journal.pone.0051422

**Published:** 2012-12-19

**Authors:** Rafael Valdés-Mas, Silvia Bea, Diana A. Puente, Carlos López-Otín, Xose S. Puente

**Affiliations:** 1 Departamento de Bioquímica y Biología Molecular, Instituto Universitario de Oncología (IUOPA), Universidad de Oviedo, Oviedo, Spain; 2 Hematopathology Unit, Hospital Clinic, IDIBAPS, Barcelona, Spain; Fred Hutchinson Cancer Research Center, United States of America

## Abstract

Exome sequencing constitutes an important technology for the study of human hereditary diseases and cancer. However, the ability of this approach to identify copy number alterations in primary tumor samples has not been fully addressed. Here we show that somatic copy number alterations can be reliably estimated using exome sequencing data through a strategy that we have termed *exome2cnv*. Using data from 86 paired normal and primary tumor samples, we identified losses and gains of complete chromosomes or large genomic regions, as well as smaller regions affecting a minimum of one gene. Comparison with high-resolution comparative genomic hybridization (CGH) arrays revealed a high sensitivity and a low number of false positives in the copy number estimation between both approaches. We explore the main factors affecting sensitivity and false positives with real data, and provide a side by side comparison with CGH arrays. Together, these results underscore the utility of exome sequencing to study cancer samples by allowing not only the identification of substitutions and indels, but also the accurate estimation of copy number alterations.

## Introduction

The development of Next Generation Sequencing (NGS) technologies has allowed the study of the human genome at an unprecedented level. Whole-genome sequencing (WGS) of several individuals has been already performed shedding new light on human variation, genome complexity and molecular mechanisms of certain hereditary diseases [1], [2]. In addition, sequencing of cancer genomes has revealed a very complex landscape of somatic mutations [3]–[5] and has led to the identification of driver genes responsible for tumor initiation and growth [6]–[9]. Despite the utility of WGS to understand human disease, this global approach is still economically unaffordable for most laboratories and does not allow the analysis of hundreds or even thousands of samples in a timely manner. In this regard, the development of technologies for the capture of specific regions of the genome [10], [11], such as all coding exons or exome, followed by NGS, has proven very useful for the rapid and economic identification of mutations in different human hereditary diseases as well as in cancer [12]–[19]. Due to the high coverage obtained using exome sequencing, this technique constitutes an interesting approach for the identification of point mutations and small indels with high accuracy in both normal and tumor samples.

Point mutations and indels constitute the most frequent alterations present in a tumor genome [4], [5], and the ability to identify them using exome sequencing represents an important achievement in cancer genomics. However, cancer cells also present other type of mutations, including translocations, inversions or changes in copy number, which constitute important events for tumor development. For instance, copy number alterations (CNAs) due to either deletion or amplification of specific regions frequently lead to deletion of tumor suppressor genes or to the amplification of oncogenes, representing driver events during tumor development [20]–[22]. In addition, some hereditary diseases are caused not by point mutations but by CNAs resulting in the deletion or amplification of specific genes, exons or regulatory sequences [23], [24]. In fact, a recent study using WGS has identified a novel CNA in *TP53* causing Li-Fraumeni syndrome [25], reinforcing the importance of CNAs in human disease. A currently assumed limitation of exome sequencing is its inability to identify this type of structural variants, and the analysis of exome data is usually complemented with other technologies, such as comparative genomic hybridization arrays (aCGH) or high throughput sequencing at low coverage in order to identify CNAs [26], [27], resulting in the requirement of additional sample material as well as increases in costs per sample. The importance of CNAs in human disease implies that the study of human pathologies by exome sequencing data must be complemented by other approaches in order to cover this type of variation.

Recent studies have shown that by using depth of coverage of individual exons, it is possible to identify copy number alterations in tumor and matched normal tissue exomes [28]–[32]. However, these methods can result in the identification of false positive CNAs due to the inherent variability of the capturing method and/or sequencing efficiency of certain regions. Therefore, to analyze the utility of this technique for cancer genomics and to define the limits of this type of analysis it is necessary to analyze exome sequencing data obtained from different individuals and processed at different times. Furthermore, a side by side comparison between exome and aCGH data is necessary to determine the sensitivity and specificity using primary tumors. In this work, we demonstrate that exome data can be used to detect tumor-specific CNAs with high accuracy and sensitivity by analyzing 86 paired normal and primary tumor samples, and show a high concordance with aCGH data. This work provides the opportunity to re-analyze existing datasets to extract this additional layer of information of great importance for human disease.

## Materials and Methods

### Samples

Sequencing and genotyping data for chronic lymphocytic leukemia (CLL) patients was obtained from the CLL-ICGC Consortium and are deposited at the European Genome-Phenome Archive (EGA, http://www.ebi.ac.uk/ega/), which is hosted at the EBI, under accession number EGAS00000000092.

### Exome capture, sequencing and mapping

Three µg of genomic DNA were fragmented to 150–200 bp and hybridized using a SureSelect Human All Exon 50 Mb Kit (Agilent) together with the Paired-End Sample Preparation Kit from Illumina following manufacturers' protocols. The captured DNA fragments were sequenced using one lane of a Genome Analyzer *IIx* (Illumina) per sample and 76 cycles, resulting in more than 30 million paired-reads per sample. Reads were aligned to the reference genome (GRCh37) using BWA-0.5.7 [33] and Samtools-0.1.7 was used to remove PCR duplicates and to create BAM files [34].

### Analysis of copy number alterations from exome data

For each sample, we counted the number of individual reads mapped within 50 bp of each of the 212,997 target regions included in the SureSelect Human All Exon 50 Mb Kit. Then, the coverage per sample was normalized to Reads mapped Per Kilobase of probe and per Million of reads mapped (RPKMs) taking into account the probe length and the total number of reads mapped within the target regions with mapping quality ≥30. To create a reference exome to be compared with individual data, we calculated for each probe the average RPKMs obtained from 31 female individuals for the analysis of the X chromosome data, and from 86 different individuals for the analysis of tumor CNAs in autosomes. The log2 ratio of tumor RPKMs to normal RPKMs from the same patient was obtained for each exon and processed using the DNAcopy package [35]. Log2 ratios were smoothed by DNAcopy and CNAs were detected using default values. Tumor CNAs were defined as those regions containing a minimum of six exons, with an average log2 ratio below −0.3 or above 0.3 as determined by DNAcopy. To remove false positives due to the presence of consecutive exons with variable capture efficiency, we performed the following procedure: i) for each exon included in the CNA region we computed the average RPKMs in all 86 normal samples (RPKM_i_^N^), as well as the standard deviation (SD_i_^N^) for the RPKMs of that exon in normal samples; ii) then, the absolute difference between the RPKMs for that exon in the tumor sample (RPKM_i_^T^) and the average RPKMs for that exon in normal samples (RPKM_i_^N^) was divided by the standard deviation obtained from the normal samples (SD_i_^N^); iii) for each potential CNA the average deviation of all exons included in that region is required to be at least 1.5.
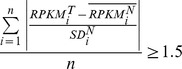



This allows to determine the deviation of tumor RPKMs from the average of normal samples. Those regions in which the average deviation was less than 1.5 SDs from the average of normal samples, and likely representing regions in which the capture efficiency of those exons was highly variable between samples, were removed. For the analysis of CNAs using the ExomeCNV package [28] we used the default parameters, and DNACopy settings were set to the same ones used before to allow a direct comparison of results. Tumor CNAs were defined as those regions containing a minimum of six exons, with an average log2 ratio below −0.3 or above 0.3. A CNA was described as supported by aCGH data when at least two exons were included within the boundaries obtained by aCGH analysis, and the copy number status (either loss or gain) was identical between both procedures.

### Comparative genomic hybridization arrays

Copy number analysis were performed in all samples hybridizing 1 µg of the test DNA and 1 µg of reference DNA on SurePrint G3 Human CGH Microarray 1 M (Agilent Technologies, Santa Clara, USA). The DNA samples hybridized were from the same preparations used for exome capture and sequencing. Raw data were generated from scanned images using Agilent Feature Extraction Software (v10.7). Log2 ratios of background corrected values for tumor over normal DNA were calculated. Post-hybridization quality control reports included DLRspread values, signal intensity, array with DLRspread over 0.3 was considered as low quality and consequently discarded. Detection of CNA was performed using the Aberration Detection Method-2 (ADM-2) algorithm implemented within the Agilent's genomics suite Genomic Workbench v5.0 with a threshold of 6.5 and a minimum of 5 consecutive probes. T-cell receptor regions rearranged in some non-tumor cells that might lead to the identification of false positive gains in the tumor were filtered for subsequent analysis.

## Results

### Detection of changes in copy number using exome sequencing

In exome sequencing, a DNA sample is captured by specific probes and then subjected to NGS, resulting in the generation of sequence reads corresponding to the target regions. Therefore, if there is a difference in copy number between two samples, the number of reads derived from that particular region should be different for both samples. However, the introduction of amplification steps during sample preparation and the limited number of bait probes which are added to the capture reaction could result in the saturation of the capturing probes, thereby hampering the identification of CNAs. In addition, the number of reads produced by different probes or in different experiments is highly variable due to several factors: i) the efficiency of the capturing procedure for a specific probe; ii) the sequencing efficiency for that particular region; iii) the total number of reads sequenced; and iv) the size of the target region. The first two factors are inherent to the technologies used for capturing and sequencing. However, they should not affect the comparison among samples, as probes with poor capturing efficiency or regions difficult to sequence should be equal for all samples processed using the same capturing protocol and sequencing technology. Regarding the last two factors, they can be easily normalized using a similar solution to that used for RNAseq experiments [36]. Thus, for any given probe the number of reads mapped can be expressed as RPKMs (Reads mapped Per Kilobase of probe and per Million of reads mapped).

Taking into account these considerations, and in order to determine whether exome sequencing data could be used to identify copy number changes between different samples, we first checked whether read coverage in the X chromosome was different between normal samples derived from males (one copy) and females (two copies). We counted all sequence reads mapped in the target regions ±50 bp with a mapping quality of more than 30. Following this scheme, we compared the average RPKMs for 55 males and 31 females. We found that the coverage (RPKMs) in the X chromosome (excluding the pseudoautosomal regions) was 12.11±0.19 for males and 23.4±0.57 for females, very close to the 1∶2 ratio expected. In contrast, the coverage in autosomes was almost identical between both groups (20.80±0.12 *vs.* 20.39±0.11). These results confirm previous studies [28]–[32] showing that the analysis of exome data can be used to detect chromosomal deletions in heterozygosity with high accuracy.

**Figure 1:**
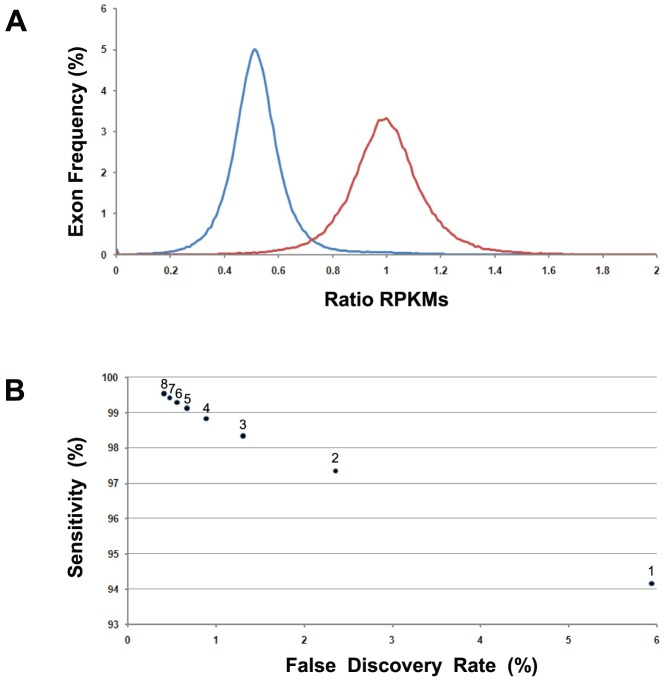
Estimation of copy number from exome sequencing data in the X chromosome of males and females. For each individual exon we calculated the coverage ratio as the ratio of RPKMs for that exon divided by the average RPKMs for that particular exon in females. (**A**) Distribution of ratios for more than 566,000 individual exons from either males (blue) or females (red). Effect of the number of consecutive exons considered for copy number estimation in sensitivity and false discovery rate. (**B**) For each different number of exons (numbers close to dots), the sensitivity is expressed as the fraction of male regions detected as copy number one when compared to the female average for the same regions. False discovery rate was calculated as the fraction of female regions detected as copy number one when compared to the female average for the same regions.

To investigate the minimum number of probes necessary to detect chromosomal deletions using exome sequencing data, and due to the variability of the exon capture procedure during sample preparation, we empirically determined this parameter by comparing real data from males and females. We selected a total of 6,588 exons that were located in the X chromosome (excluding the pseudoautosomal regions) and were efficiently captured by this technology, as they had at least a minimum of two RPKMs in females. For each individual exon, we calculated the average RPKMs obtained in females, and used this value as the reference RPKMs for that particular exon in individuals with two copies of the X chromosome. Then, for each of the 566,568 exons analyzed in 86 individuals we calculated the ratio of RPKMs for each individual exon *versus* the reference value calculated before. There was a marked difference in this ratio between males and females for most individual exons (Figure 1A). In fact, 94% of exons from males had RPKM ratios which were at less than −1.5 SDs from the female average for that particular exon, while only 6% of exons from females were at less than −1.5 SDs. The sensitivity to detect this change in copy number increased as a larger number of consecutive exons were used, while the percentage of false positives decreased (Figure 1B). In this sense, we determined that by using a minimum of six exons, 99.29% of male *loci* were at less than −1.5 SDs from the female average, and 99.43% of female *loci* were at more than −1.5 SDs, suggesting that these parameters could be used to detect copy number changes with high sensitivity and a low false discovery rate.

### Identification of tumor-specific CNAs using exome sequencing data

**Figure 2:**
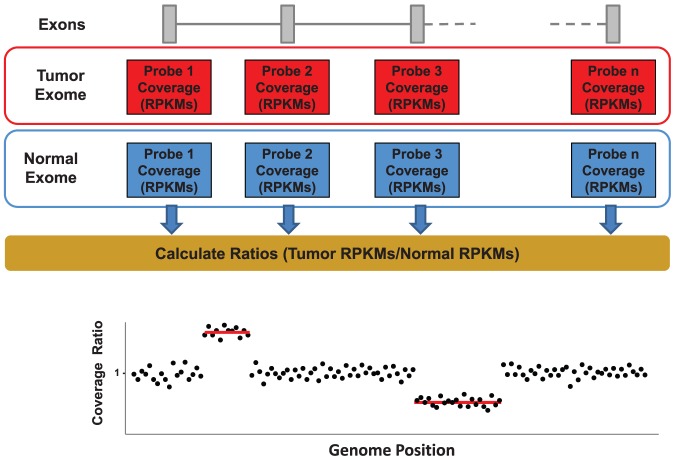
Scheme depicting the strategy used by *exome2cnv* for detecting CNAs using exome coverage data for a tumor sample and a normal sample from the same patient. Normalized coverage (RPKMs) is determined for each individual capturing exon, and the ratio tumor/normal is calculated for each probe. Genome-wide analysis of ratios allows the identification of regions having somatic copy number alterations in the tumor (red lines).

An important field for the application of technologies allowing the identification of CNAs is cancer genomics, as a large number of somatic mutations affecting oncogenes or tumor suppressor genes involve either amplification or deletion of the corresponding *loci*. To determine whether chromosomal gains or losses as well as smaller CNAs could be detected in tumor samples using exome sequencing data, we studied 86 CLL samples known to have changes in copy number by aCGH [8], [37]. CLL represents an interesting model because this tumor type usually has very few CNAs [8], what allows an accurate estimation of the number of false positive calls by novel approaches as the one described in this study. For this aim, we developed a strategy to identify CNAs using exome data that we called *exome2cnv* (Figure 2). Thus, for each single capturing exon we compared the log2 ratio of the RPKMs obtained from the tumor sample to the RPKMs obtained from the normal sample, and applied a circular binary segmentation algorithm (DNAcopy) to identify regions potentially lost or gained in the tumor sample [35]. To reduce the noise introduced due to exons with poor capturing efficiency, we selected only those exons having at least two RPKMs in the normal sample from the same patient (>89% of the exons). For all those cases, in addition to exome data we also had available aCGH data for tumor and normal samples (see Material and Methods), what allowed us to compare the results of the *exome2cnv* approach in terms of sensitivity and false positives.

**Figure 3:**
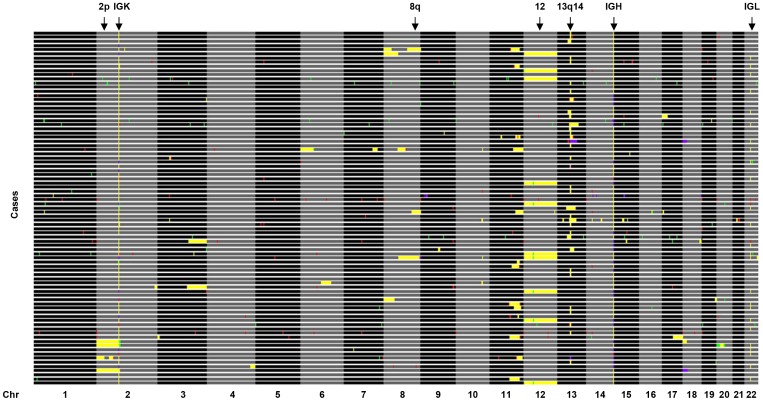
Comparison of CNAs obtained by *exome2cnv* and aCGH in 86 CLL cases. CNAs were classified in four different classes represented by different colors in the figure. CNAs detected by both *exome2cnv* and aCGH approaches are labeled in yellow; those detected specifically by *exome2cnv* are shown in green; CNAs only detected by aCGH are shown in red; and CNAs detected by *exome2cnv* and considered as subclones by aCGH or corresponding to immunoglobulin regions are shown in purple. Regions recurrently altered in CLL are indicated on top.

**Figure 4:**
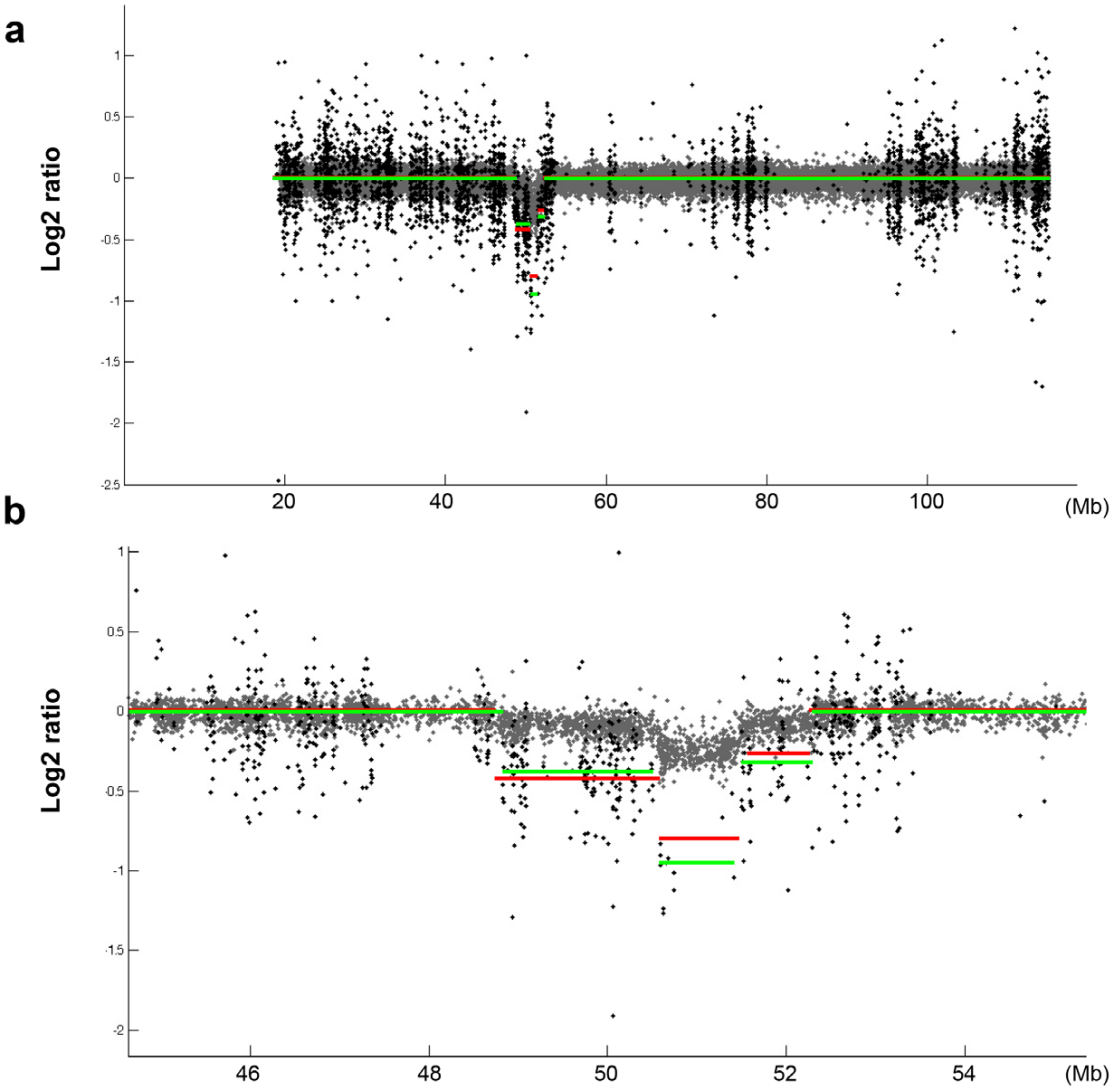
Comparison between CNAs detected by *exome2cnv* and aCGH. Grey dots represent log2 ratios of tumor/normal probe intensities from aCGH, while black dots show log2 ratios of tumor/normal from exome sequencing data. The local averages determined for exome data (red lines) and aCGH data (green lines) are shown. (**a**) Homozygous deletion of a small region of chromosome 13 detected by both approaches. (**b**) Detailed view of the same chromosomal region shown in (a).

By comparing exome data from the tumor sample with its matched non-tumor cells we were able to detect several somatic CNAs affecting autosomes which were also found using aCGH data (Figure 3 and Tables S1 and S2). They involved homozygous or heterozygous deletion of large chromosomal regions (in chromosomes 6, 11, 13, 17 or 20), gains of whole chromosomes (chromosome 12) or large chromosomal regions (in chromosomes 2, 3, and 4), as well as other smaller regions including deletion of the *RFX7* gene or deletion of six exons of *SLC9A9*. In addition, we detected both homozygous and heterozygous deletions of a small fragment of chromosome 13q14 frequently deleted in CLL tumors [8], [38], [39] (Figure 3 and Figure 4) and resulting in the deletion of two microRNAs (*miR15a* and *miR16-1*) frequently lost in this pathology [38]. Trisomy of chromosome 12, a frequent alteration present in CLL tumors, was also identified using *exome2cnv*. Together, these results show that this procedure allows the identification of most types of CNAs that might be present in cancer samples, including heterozygous and homozygous deletions as well as amplified genomic regions.

### Comparison between exome2cnv and aCGH data

To estimate the sensitivity and the number of false positives of this approach, we compared the data obtained by *exome2cnv* with that obtained by aCGH. Thus, using the exome data we identified 387 CNAs out of the 549 detected by aCGH (70%). However, as previously shown in Figure 1B and due to the variability of exon capture, it would be necessary to combine at least six exons to make a reliable CNA call. Using this cutoff for aCGH regions, only 44 out of the 162 aCGH regions not detected by *exome2cnv* fulfilled this criterion, suggesting that the *exome2cnv* sensitivity to detect tumor-specific CNAs is more than 89%. In agreement with these results, analysis of these 86 tumor-normal pairs with a previously described method [28] resulted in the identification of 22,423 potential CNAs, with more than 18,000 of them corresponding to single-exon CNAs which were not supported by aCGH data. These calls likely constitute false positives due to differences in the capturing efficiency or to a batch effect. In fact, the introduction of a minimum number of six consecutive exons to make a call still resulted in more than 80% of calls not being supported by aCGH data. In this regard, a detailed examination of these calls revealed than in most cases they were supported by consecutive exons showing either small RPKMs, high GC content and high variability between different samples, suggesting poor reproducibility in the capture efficiency. Calls reporting a putative gain, an infrequent event in CLL with the exception of chromosome 12 trisomy, were particularly sensitive to this variability issue, with more than 95% of the calls not being supported by aCGH data. Together, these results reinforce the importance of establishing specific parameters affecting the performance of exome sequencing-based CNA estimation, such as minimum number of exons, and to take into account the RPKM variance across normal samples, which are used by the *exome2cnv* method described herein.

It is interesting to notice that as CLL cells derive from B-lymphocytes, which had undergone rearrangement of immunoglobulin genes during B-cell maturation, we were able to detect in almost all analyzed cases focal homozygous or heterozygous deletions in chromosomes 2p11, 14q32.33 and 22q11.22, where immunoglobulin genes are located (Figure 3). However, when these regions were omitted from the analysis, we obtained the same sensitivity, indicating that *exome2cnv* has enough sensitivity to detect most oncogenic CNAs. Furthermore, when we compared the number of exons included in CNAs affecting subchromosomal regions between the *exome2cnv* approach and aCGH data, we obtained a high correlation between both approaches (r^2^ = 0.99) (Figure S1), suggesting that CNA boundaries detected by both methods are highly similar in terms of exons involved in the copy number change.

On the other hand, we found that more than 86% of the CNAs detected by *exome2cnv* overlapped with CNAs detected by aCGH (819/947). Although it is possible that some of the 128 regions detected specifically by the *exome2cnv* approach might constitute false positives, manual inspection of the aCGH data for these regions revealed that at least 16 of them could be considered CNAs present in a subpopulation of CLL cells (Figure 3 and Table S2 and Figure S2). Furthermore, another 37 of them were located in *loci* containing immunoglobulin genes and putatively representing real CNAs. Together, these data show that using the minimum threshold of six exons empirically determined before, more than 92% of identified regions might constitute *bona fide* somatic CNAs.

## Discussion

Exome sequencing, using target capture strategies followed by NGS, is becoming a routine technique for the study of somatic mutations in tumor samples as well as for the identification of the genetic alterations responsible for numerous hereditary diseases [9], [14], [15], [18], [40]. Due to the target capture approach, exome sequencing analysis has limitations to uncover the different types of variations present in a cancer genome, and its use is mostly limited to the study of substitutions and small indels. The results presented herein demonstrate that exome sequencing data can be also used to estimate copy number alterations with high accuracy, allowing the identification of somatic CNAs in tumor/normal samples from cancer patients.

The ability to detect CNAs using exome sequencing data has been recently proposed [28]–[32]. However, the real utility of this approach for the study of cancer exomes requires the analysis of a larger number of primary tumors. In this study, we have used a liquid capture technique and the target regions which were initially designed by the International Cancer Genome Consortium (ICGC) [41] to analyze CNAs in a total of 86 primary CLL cases using exome sequencing data. We performed a side by side comparison with traditional CGH technologies in order to determine the sensitivity of this novel approach. The results obtained in this initial approach demonstrate that *exome2cnv* has the potential to identify most CNAs affecting exon-containing regions. Based on our data, the *exome2cnv* approach would allow the identification of either long CNAs containing hundreds of genes, or small regions affecting up to a single gene or a single exon. In addition, we have determined the proportion of false positives of this approach, as most researchers would like to know how many regions identified by *exome2cnv* are *bona fide* CNAs, as well as the parameters affecting the number of false positives. We have shown that similar to other methods, there is a balance between sensitivity and false discovery rate, and by increasing the number of exons to call a CNA it is possible to reduce the number of false positives. Our procedure differs from other previously described methods in two basic aspects. First, we empirically determine a minimum number of exons to make a CNA call and only exons with a minimum coverage in the normal sample are used in the analysis. All described methods for the identification of somatic CNAs from exome-sequencing data, including the one described here, are able to detect CNAs affecting a single exon. However, due to the different efficiency in capturing of specific probes or sequencing of certain genomic regions, side by side comparison of two samples processed independently might lead to the erroneous classification of numerous exons as CNAs. In fact, a direct comparison of exon coverage using previously reported methods results in the identification of more than 200 single-exon CNAs per case not supported by aCGH data, suggesting that they constitute false positives. In our method this effect can be substantially reduced by using only exons with a good capture efficiency, defined here as more than 2 RPKMs, and by requiring at least a minimum number of consecutive exons to make a call, established here as 6 or more consecutive exons. The second difference with previously reported methods is the use of coverage data from normal samples. In fact, a common problem during the analysis of samples captured or sequenced at different times or in different centers is the inter-sample variability in capture efficiency in specific regions/probes. Although this variability has a minor effect in the identification of point-mutations and/or indels, it represents a common problem for the identification of CNAs, resulting in the identification of many false positives [28]. In order to reduce the false discovery rate of this method, we have introduced a step that takes into account not only the different coverage between tumor and normal samples, but also the coverage distribution along all non-tumor samples available. Only those regions supported by exons whose coverage differ more than 1.5 SDs from the average coverage of those exons in non-tumor samples are considered as CNAs. Using this approach, we could confirm that more than 92% of the CNA regions identified using *exome2cnv* were also detected using CGH arrays, strongly supporting the utility of this method for the analysis of CNA in cancer exomes. Together, these filtering steps are able to reduce the number of false positives CNA regions identified by *exome2cnv*, while maintaining a sensibility of more than 89% to detect CNAs involving at least six exons. Although a lower number of exons could be used, this would likely increase the number of false positives. This approach can benefit the identification of somatic CNAs in tumor/normal samples from cancer patients, as they usually involve amplification or deletion of chromosomal regions containing several genes, facilitating their detection by *exome2cnv* approach. In fact, the sensibility to detect CNAs involving whole chromosomes or chromosome arms is almost 100%.

Despite the overall performance of the *exome2cnv* approach for the identification of tumor-specific CNAs, an inherent limitation of this approach when compared to aCGH data is the accurate determination of the CNA boundaries. Thus, the distribution of aCGH probes in high resolution arrays allows a precise estimation of CNA boundaries within kilobase resolution. However, the uneven distribution of exons throughout the genome results in less accurate boundaries in terms of genomic distances, but in a highly accurate determination of genes and exons involved in the copy number alteration. In this regard, an alternative approach that might be considered by the manufacturers of target-capture reagents is the introduction of additional probes in exon-poor regions. These extra probes would not help in the identification of substitutions in coding regions, but would improve the estimation of CNA length and boundaries.

Another aspect to take into consideration when using primary tumors is the presence of normal cell contamination in the tumor sample. Although the tumor samples used in this study had more than 95% tumor cell content, some of the CNAs detected by our method appeared to be present in a subpopulation of tumor cells. These data indicates that *exome2cnv* is suitable for the detection of CNAs in complex populations, as those present in most solid tumors, in which stromal cell contamination is usually present. Moreover, it is important to point out that copy number changes in the tumor also complicate the identification of somatic mutations, and a precise estimation of tumor copy number is necessary in order to adjust mutation calling algorithms [42].

In summary, we show that copy number changes can be accurately determined using exome sequencing, extending the application of this widely used technique for the study of human disease, and allowing the identification of variations outside of target regions used for capture. This application is of particular interest to the field of cancer genomics, as CNAs represent an important mechanism of mutation in most cancer types. The analysis of a large number of primary tumor exomes and aCGH data has allowed the first determination of the sensitivity and false discovery rate of this approach. Together, the procedure outlined here can be used to rapidly analyze existing datasets without additional experimental work, what will facilitate the identification of novel CNAs implicated in cancer.

## Supporting Information

Table S1 Comparison of somatic CNAs detected by aCGH vs. exome2cnv to determine sensitivity.
(PDF)

Table S2 Comparison of somatic CNAs detected by exome2cnv vs. aCGH to determine false discovery rate.
(PDF)

Figure S1 **Comparison of the number of exons involved in CNAs detected by exome2cnv and aCGH.**
(PDF)

Figure S2 **aCGH data showing two cases (A and B) with a deletion affecting the short arm of chromosome 18 which was detected by exome2cnv but not by aCGH because it is present in a subpopulation of tumor cells.** For comparison, a case (C) with a deletion affecting all tumor cells and detected by both methods is shown.
(PDF)
