# Supplementary material for: Estimation of Copy Number Alterations from Exome Sequencing Data
Source: PLoS One. 2012 Dec 19;7(12):e51422. doi: 10.1371/journal.pone.0051422 (PMC3526607; doi:10.1371/journal.pone.0051422)
Supplement: Table S1 — Comparison of somatic CNAs detected by aCGH vs. exome2cnv to determine sensitivity. (PDF) [file pone.0051422.s001.pdf]

aCGH							EXOME					
CASE	CHROM	START	END	LOG2RATIO	# EXONS	FOUND	CHROM	START	END	LOG2RATIO	# EXONS	StdDev
CLL270TD	chr1	15929364	15943278	-0.7922	0	NO						
CLL027TD	chr1	21681195	21725823	-0.7741	0	NO						
CLL145TD	chr1	41572891	43287342	-0.7145	110	YES	chr1	41578866	43282098	-0.8159	110	-3.3368
CLL152TD	chr1	48823119	48879073	-0.9446	8	NO						
CLL155TD	chr1	78433832	78452941	-0.8181	4	YES	chr1	78432316	78444463	-0.8221	8	-3.1091
CLL152TD	chr1	113461274	113580282	-0.8756	2	NO						
CLL276TD	chr1	153967305	154126545	-0.2614	36	NO						
CLL117TD	chr1	197743592	197752665	0.9784	0	NO						
CLL178TD	chr1	202322285	202538415	0.2841	29	NO						
CLL100TD	chr1	231918632	231957257	-0.4835	5	NO						
CLL152TD	chr1	249197503	249220091	-0.6401	2	NO						
CLL027TD	chr10	503201	569067	-0.5479	3	NO						
CLL049TD	chr10	102641828	106419703	-0.7237	638	YES	chr10	102675717	106214143	-0.8608	638	-3.2902
CLL141TD	chr10	103634312	104177223	-1.0034	133	YES	chr10	103649117	104176108	-1.0251	133	-3.9548
CLL178TD	chr10	104159664	104853373	-0.7501	130	YES	chr10	104160862	104852860	-0.8987	126	-2.9443
CLL064TD	chr10	104161255	104192055	-0.7374	22	YES	chr10	104160694	104196244	-0.6812	25	-1.9207
CLL157TD	chr10	104368438	104419240	-0.3115	12	NO						
CLL178TD	chr10	105007763	105100437	-0.8027	7	YES	chr10	105036937	105093635	-0.8543	7	-3.1110
CLL017TD	chr11	33880105	33895469	-1.1002	2	NO						
CLL184TD	chr11	42655597	45369571	-0.7617	140	YES	chr11	43333651	45277209	-0.9153	140	-3.4682
CLL042TD	chr11	76513928	117661843	-0.4171	2028	YES	chr11	76956316	116656464	-0.4984	1805	-2.9969
CLL023TD	chr11	77179449	117609214	-0.8262	1961	YES	chr11	77300986	77924655	-1.0005	80	-3.5747
CLL006TD	chr11	78240712	116777756	-0.9000	1734	YES	chr11	78270534	89449046	-0.9866	331	-3.9747
CLL038TD	chr11	78764165	80319824	-0.5023	4	NO						
CLL290TD	chr11	79590720	116062438	-0.8491	1643	YES	chr11	82533422	89608133	-0.9468	311	-3.8275
CLL155TD	chr11	85264544	85274227	-2.6815	0	NO						
CLL054TD	chr11	86053929	114937176	-0.7975	1462	YES	chr11	86055583	114578256	-0.9208	1462	-3.8902
CLL117TD	chr11	88542659	88551726	0.9070	0	NO						
CLL178TD	chr11	92513723	133254506	-0.8701	2738	YES	chr11	92523066	102398480	-0.8893	400	-4.3660
CLL041TD	chr11	94769441	123056760	-0.6585	1930	YES	chr11	94800334	111941112	-0.6348	834	-3.0826
CLL278TD	chr11	96159104	116753164	-0.8110	1063	YES	chr11	99690199	116747578	-0.9151	1061	-4.0853
CLL184TD	chr11	101194338	117964476	-0.5119	1171	YES	chr11	101324358	103027067	-0.8296	178	-4.1564
CLL145TD	chr11	101389939	134945306	-0.7980	2517	YES	chr11	101761989	134320539	-0.8880	2517	-3.7222
CLL017TD	chr11	105820665	128506612	-0.8830	1913	YES	chr11	105836610	128564063	-0.9707	1914	-3.7459
CLL063TD	chr11	106420522	108122884	-0.8464	128	YES	chr11	106558228	108122540	-1.0001	128	-4.2278
CLL038TD	chr11	109139541	113781239	-0.6921	338	YES	chr11	109294356	113779976	-0.7561	338	-3.2607
CLL063TD	chr11	110211047	116153447	-0.8626	388	YES	chr11	110306499	115110946	-0.9450	388	-3.9442
CLL100TD	chr11	134890599	134945306	-0.5205	0	NO						
CLL027TD	chr11	134901640	134945306	-0.6690	0	NO						
CLL152TD	chr11	134908232	134945306	-0.9068	0	NO						
CLL275TD	chr12	163393	34756350	0.5169	2808	YES	chr12	148430	346239	0.7021	36	3.3612
CLL005TD	chr12	163393	34533264	0.5302	2821	YES	chr12	148430	48191104	0.5231	3471	3.5899
CLL277TD	chr12	163393	34756350	0.4735	2822	YES	chr12	73256	6438371	0.4235	590	2.5946
CLL148TD	chr12	189361	34756350	0.4976	2776	YES	chr12	278134	347073	0.8518	26	3.1842
CLL064TD	chr12	189361	34417592	0.5136	2804	YES	chr12	148430	645362	0.4080	81	3.0082
CLL045TD	chr12	189361	34756350	0.4822	2805	YES	chr12	148999	3149497	0.4568	352	2.6347
CLL082TD	chr12	191809	34756350	0.5293	2769	YES	chr12	332288	6861035	0.4501	740	2.5129
CLL032TD	chr12	191809	34756350	0.4431	2789	YES	chr12	87509	7343411	0.5589	965	3.0818
CLL282TD	chr12	194049	34756350	0.5133	2789	YES	chr12	148430	6125206	0.5052	525	3.1675
CLL166TD	chr12	250702	34360180	0.4152	2810	YES	chr12	148430	9596082	0.5140	1354	3.2107
CLL282TD	chr12	37957940	133779217	0.4997	8113	YES	chr12	8329466	48119078	0.5237	2283	3.7832
CLL275TD	chr12	37957940	133779217	0.5145	8151	YES	chr12	31265139	48104523	0.4897	745	2.9494
CLL277TD	chr12	37957940	133779217	0.4656	8159	YES	chr12	9726035	50474278	0.4501	2606	2.8220
CLL082TD	chr12	38448467	133779217	0.5185	8096	YES	chr12	9747823	48096426	0.5740	2020	4.4115
CLL064TD	chr12	38448467	133779217	0.4811	8132	YES	chr12	33529740	48372310	0.5683	688	4.1040
CLL032TD	chr12	38453410	133779217	0.4287	8061	YES	chr12	31242290	48096426	0.4137	758	4.1407
CLL005TD	chr12	38453410	133779217	0.5251	8176	YES	chr12	148430	48191104	0.5231	3471	3.5899
CLL148TD	chr12	38503256	133779217	0.4875	8100	YES	chr12	31265139	48104523	0.4001	744	3.3445
CLL045TD	chr12	38503256	133779217	0.4753	8112	YES	chr12	3310330	57596111	0.4915	5249	3.0283
CLL166TD	chr12	38572477	133779217	0.3933	8121	YES	chr12	9747823	120802447	0.5088	8316	3.3625
CLL192TD	chr12	57562548	57636170	0.2970	80	NO						
CLL023TD	chr12	96282247	96330768	-0.4998	9	NO						
CLL027TD	chr12	96284782	96330768	-0.5886	7	NO						
CLL145TD	chr12	121860338	123047826	-0.7281	222	YES	chr12	121861133	123048222	-0.8112	223	-3.0364
CLL141TD	chr13	20906308	22405579	-0.9435	111	YES	chr13	20978234	21729228	-0.9476	72	-3.7309
CLL146TD	chr13	34752795	70922816	-0.7916	1217	YES	chr13	34837007	70681587	-0.9006	1217	-3.7898
CLL110TD	chr13	38733308	53483162	-0.7936	919	YES	chr13	38923997	52293288	-0.7827	764	-3.3982
CLL194TD	chr13	41434188	52354921	-0.9053	667	YES	chr13	41485871	44476730	-0.9826	204	-3.6015
CLL182TD	chr13	41469090	42757672	-0.3043	123	NO						
CLL321TD	chr13	41561808	52237129	-0.8764	652	YES	chr13	41635750	50510258	-0.8777	585	-3.9145

CLL189TD	chr13	46334152	82152329	-0.4679	931	YES	chr13	46357452	80910886	-0.5062	931	-2.0621
CLL192TD	chr13	46913252	51522316	-0.7334	254	YES	chr13	46917419	51522103	-0.7799	254	-3.9047
CLL141TD	chr13	46915859	73435221	-0.8720	596	YES	chr13	46917419	73428183	-0.9020	596	-3.6167
CLL188TD	chr13	47295273	53161169	-0.9120	395	YES	chr13	47297320	50505091	-1.0137	196	-3.8394
CLL267TD	chr13	47507142	62915894	-0.7481	462	YES	chr13	48517452	62334304	-0.8706	462	-3.6216
CLL030TD	chr13	47563657	53040052	-0.8205	363	YES	chr13	48517452	53016485	-0.8124	355	-3.4252
CLL083TD	chr13	47946084	65226370	-0.8482	470	YES	chr13	48517452	64417580	-0.9101	470	-3.6942
CLL027TD	chr13	48501167	51571802	-0.8240	202	YES	chr13	48517452	51527990	-0.8010	201	-3.5821
CLL272TD	chr13	48569200	51600231	-0.6386	204	YES	chr13	48570947	49822963	-0.9082	88	-3.8393
CLL172TD	chr13	48629192	50438731	-0.7113	163	YES	chr13	48651240	50366552	-0.7516	163	-3.3188
CLL279TD	chr13	48703533	52885510	-0.8388	318	YES	chr13	48708461	52863912	-0.8923	318	-3.8150
CLL181TD	chr13	48740315	52314639	-0.4214	230	YES	chr13	48827887	50505091	-0.3781	159	-1.6611
CLL186TD	chr13	48740315	52707274	-0.5630	293	YES	chr13	48827887	52701409	-0.5998	293	-2.8210
CLL184TD	chr13	48858287	58200326	-0.7885	391	YES	chr13	48881358	57747585	-0.8533	391	-3.6616
CLL117TD	chr13	48909694	51700648	-0.5751	185	YES	chr13	48916672	50495604	-0.4543	149	-2.1713
CLL053TD	chr13	48919079	51700648	-0.6254	186	YES	chr13	48941624	51603719	-0.5964	179	-2.8316
CLL013TD	chr13	49759884	51581416	-0.7134	121	YES	chr13	49760048	50123538	-0.6573	62	-2.9704
CLL157TD	chr13	49813764	51593526	-0.8986	113	YES	chr13	49822963	51594513	-0.9695	114	-4.0961
CLL144TD	chr13	50389525	51568793	-0.7145	27	YES	chr13	50366552	51508957	-0.7432	22	-2.7499
CLL280TD	chr13	50432170	51645041	-0.8038	39	YES	chr13	50489187	51397432	-1.0093	15	-3.9806
CLL172TD	chr13	50506691	51479294	-1.7397	13	YES	chr13	50510258	50747127	-1.9621	10	-6.6197
CLL274TD	chr13	50516370	50568119	-0.6611	0	NO						
CLL136TD	chr13	50516370	51526121	-0.7490	20	YES	chr13	50586025	51504796	-0.8482	15	-3.0379
CLL174TD	chr13	50520557	51527535	-2.6065	21	YES	chr13	50586025	51417260	-3.6344	13	-7.3596
CLL159TD	chr13	50557545	51375978	-1.2829	10	YES	chr13	50586025	51287304	-1.5641	10	-5.6372
CLL040TD	chr13	50557545	51372504	-2.1297	10	YES	chr13	50586025	51287304	-4.5222	10	-8.5208
CLL052TD	chr13	50559222	51551357	-0.4880	22	YES	chr13	50586025	51523559	-0.4579	20	-1.9180
CLL274TD	chr13	50569953	51469532	-1.6291	12	YES	chr13	50586025	51416933	-2.2354	12	-6.4344
CLL323TD	chr13	50569953	51483819	-0.6924	13	YES	chr13	50495604	51417260	-0.6220	16	-3.3454
CLL041TD	chr13	50608201	51501750	-0.6374	11	YES	chr13	50623062	51501518	-0.6784	11	-2.7956
CLL322TD	chr13	50616393	51553735	-2.4118	20	YES	chr13	50623062	51530431	-3.5042	20	-6.8704
CLL040TD	chr13	51377832	51540139	-0.3716	12	YES	chr13	51397432	51530431	-0.9708	12	-3.9321
CLL323TD	chr13	51490556	51626656	-0.2578	22	NO						
CLL280TD	chr13	51646339	52126200	-0.2566	30	NO						
CLL322TD	chr13	57759170	57787540	0.7874	0	NO						
CLL184TD	chr13	61436425	61921871	-0.8754	0	NO						
CLL027TD	chr13	115079411	115105438	-0.7692	1	NO						
CLL152TD	chr13	115086654	115105438	-0.8995	1	NO						
CLL141TD	chr14	20760146	20765549	-0.9383	4	YES	chr14	20697540	20764486	-0.7731	7	-2.4436
CLL027TD	chr14	20964303	20983196	-0.7496	2	NO						
CLL027TD	chr14	21155125	21201487	-0.6704	2	NO						
CLL277TD	chr14	22965689	22994535	0.5488	24	NO						
CLL141TD	chr14	58585617	58808196	-0.9673	37	YES	chr14	58598184	58796653	-0.9766	37	-3.7360
CLL054TD	chr14	106168179	106330568	-0.3498	31	NO						
CLL159TD	chr14	106168179	106410678	-0.7433	62	YES	chr14	106130944	106329377	-1.3216	37	-2.2826
CLL144TD	chr14	106168179	106777490	-0.9957	96	YES	chr14	106174088	106209080	-1.2127	10	-2.5395
CLL186TD	chr14	106168179	106811048	-1.0292	109	YES	chr14	106204080	106237415	-5.5469	15	-4.5364
CLL052TD	chr14	106168179	107015590	-1.4376	124	YES	chr14	106130944	106330387	-1.7185	36	-3.0434
CLL145TD	chr14	106168179	107083152	-0.6834	141	YES	chr14	106204080	106209080	-2.6671	6	-4.4185
CLL174TD	chr14	106243009	106322375	-0.7534	9	YES	chr14	106232232	106329987	-0.8755	20	-2.0385
CLL039TD	chr14	106243009	106538621	-1.0335	17	NO						
CLL290TD	chr14	106243009	106561323	-0.8239	55	YES	chr14	106304633	106329987	-1.0331	13	-2.3069
CLL178TD	chr14	106243009	106691076	-0.9101	68	YES	chr14	106232232	106321000	-1.2260	17	-2.4237
CLL009TD	chr14	106243009	106734526	-0.7843	79	YES	chr14	106304633	106330387	-0.8045	12	-1.7481
CLL017TD	chr14	106243009	106827263	-1.0022	82	YES	chr14	106232232	106329377	-0.9118	19	-2.3284
CLL040TD	chr14	106243009	106827263	-0.3900	88	YES	chr14	106232232	106330760	-1.2225	22	-2.2656
CLL110TD	chr14	106243009	107094934	-0.7319	126	YES	chr14	106232232	106330387	-1.1768	21	-2.5712
CLL008TD	chr14	106243009	107167303	-0.8026	131	YES	chr14	106304633	106329987	-0.7630	12	-2.1526
CLL045TD	chr14	106243009	107179117	-0.7934	132	YES	chr14	106232232	106330760	-3.0340	24	-3.6521
CLL136TD	chr14	106252596	106303121	-0.7909	0	YES	chr14	106237415	106329987	-0.6444	11	-2.3135
CLL276TD	chr14	106252596	106303121	-0.8381	0	YES	chr14	106188116	106329377	-1.0992	28	-1.9216
CLL280TD	chr14	106252596	106322375	-0.7332	9	YES	chr14	106232232	106329987	-1.0074	20	-2.3658
CLL172TD	chr14	106252596	106322375	-0.7866	10	YES	chr14	106304633	106321986	-1.0642	10	-2.3240
CLL322TD	chr14	106252596	106411088	-0.9570	45	YES	chr14	106204080	106329987	-1.0533	28	-1.6795
CLL048TD	chr14	106252596	106561323	-1.9852	51	YES	chr14	106188116	106366443	-3.4155	37	-3.7502
CLL278TD	chr14	106252596	106691076	-0.6082	70	YES	chr14	106304633	106329987	-0.8399	12	-1.6766
CLL064TD	chr14	106252596	106779188	-0.8215	83	YES	chr14	106232232	106329377	-0.9631	21	-2.2028
CLL197TD	chr14	106252596	106829408	-0.7154	87	YES	chr14	106232232	106330387	-1.0805	21	-1.9559
CLL043TD	chr14	106252596	106829408	-1.7132	88	YES	chr14	106232232	106307218	-2.8044	11	-4.2632
CLL173TD	chr14	106252596	106831538	-1.9681	92	YES	chr14	106304633	106405971	-9.9367	45	-3.6496
CLL191TD	chr14	106252596	107012899	-0.8324	107	YES	chr14	106235554	106329987	-0.6049	18	-1.7845
CLL192TD	chr14	106252596	107208787	-0.6230	137	YES	chr14	106188116	106346841	-4.7516	37	-3.9438
CLL184TD	chr14	106257663	107048607	-0.8605	120	YES	chr14	106237415	106330387	-1.0780	15	-1.6901

CLL282TD	chr14	106263491	106322375	-0.8652	11	YES	chr14	106304633	106329987	-0.7720	13	-1.9926
CLL023TD	chr14	106320095	107169498	-0.7311	122	YES	chr14	106329987	106379028	-7.5893	25	-2.8851
CLL027TD	chr14	106320095	107182803	-0.8139	131	YES	chr14	106330387	106356996	-8.9239	11	-2.8816
CLL157TD	chr14	106326556	106526710	-0.9814	43	YES	chr14	106330760	106363769	-9.9654	14	-3.0537
CLL141TD	chr14	106330168	106345551	-4.2148	3	YES	chr14	106329987	106375713	-9.9491	20	-3.1210
CLL032TD	chr14	106330168	106345551	-3.2441	3	YES	chr14	106330387	106379028	-6.9021	21	-2.9824
CLL018TD	chr14	106330168	106345551	-4.5924	3	NO						
CLL053TD	chr14	106330168	106345551	-3.5836	3	YES	chr14	106330760	106379028	-7.8069	23	-2.9223
CLL277TD	chr14	106330168	106345551	-3.9939	4	YES	chr14	106329987	106378065	-9.7456	25	-2.9875
CLL279TD	chr14	106330168	106345551	-3.1367	4	YES	chr14	106329377	106359349	-3.3636	15	-2.5594
CLL266TD	chr14	106330168	106345551	-3.2449	5	YES	chr14	106330387	106370493	-7.8500	21	-2.9798
CLL083TD	chr14	106330168	106345551	-3.4146	5	YES	chr14	106330387	106357506	-8.6562	12	-2.8731
CLL100TD	chr14	106330168	106345551	-3.3787	5	YES	chr14	106329377	106346841	-9.0224	8	-2.8042
CLL022TD	chr14	106330168	106345551	-3.2690	5	NO						
CLL276TD	chr14	106330168	106345551	-2.8459	5	NO						
CLL282TD	chr14	106330168	106411088	-2.6317	27	YES	chr14	106349710	106405971	-9.7542	21	-3.6324
CLL051TD	chr14	106330168	106517682	-0.6045	35	YES	chr14	106382639	106518370	-0.6511	15	-2.2766
CLL007TD	chr14	106330168	106517682	-2.0845	37	YES	chr14	106330387	106494494	-3.3737	37	-3.1163
CLL148TD	chr14	106330168	106526710	-0.9501	39	YES	chr14	106330760	106369421	-6.0154	17	-2.9558
CLL041TD	chr14	106330168	106524457	-0.7176	39	YES	chr14	106329987	106370493	-1.7754	20	-2.1101
CLL172TD	chr14	106330168	106538621	-2.1448	40	YES	chr14	106329377	106379028	-9.7387	26	-2.9754
CLL038TD	chr14	106330168	106561323	-1.6025	40	YES	chr14	106330760	106369421	-2.6953	15	-2.5613
CLL006TD	chr14	106330168	106526710	-1.0101	40	YES	chr14	106346841	106356996	-4.5195	6	-2.8648
CLL274TD	chr14	106330168	106610162	-1.1555	46	YES	chr14	106329377	106405971	-9.7964	28	-3.4616
CLL181TD	chr14	106330168	106610162	-0.9352	50	YES	chr14	106330387	106380172	-10.0000	24	-3.0424
CLL275TD	chr14	106330168	106610162	-0.8714	51	YES	chr14	106330760	106351843	-10.0000	8	-2.9762
CLL188TD	chr14	106330168	106723316	-0.7276	59	YES	chr14	106349710	106714578	-0.9977	54	-2.2479
CLL019TD	chr14	106330168	106724962	-1.7993	61	YES	chr14	106330387	106579220	-9.5583	46	-3.9811
CLL124TD	chr14	106330168	106724962	-0.5153	61	YES	chr14	106329377	106714578	-0.7779	63	-1.8724
CLL090TD	chr14	106330168	106724962	-1.5966	62	YES	chr14	106346841	106370309	-3.8011	14	-2.9557
CLL323TD	chr14	106330168	106691076	-0.8079	62	YES	chr14	106330760	106471216	-8.5546	34	-3.5535
CLL174TD	chr14	106330168	106724962	-2.2069	66	YES	chr14	106346841	106386891	-9.9147	25	-3.0002
CLL020TD	chr14	106330168	106786127	-0.7892	67	YES	chr14	106331373	106351843	-9.4122	7	-2.8086
CLL042TD	chr14	106330168	106852841	-0.5597	74	YES	chr14	106330760	106478072	-1.5875	27	-3.1102
CLL189TD	chr14	106330168	106918315	-1.3986	83	YES	chr14	106330387	106369421	-8.2337	16	-2.9375
CLL273TD	chr14	106330168	106877502	-0.7216	84	YES	chr14	106330387	106373023	-4.3847	20	-2.5511
CLL013TD	chr14	106330168	106877502	-0.8658	85	YES	chr14	106330760	106453023	-9.2941	31	-3.5583
CLL280TD	chr14	106330168	107012899	-2.3117	97	YES	chr14	106330387	106388010	-9.9417	26	-3.1735
CLL049TD	chr14	106330168	106993731	-0.8750	98	YES	chr14	106330760	106552646	-4.2708	43	-3.4485
CLL029TD	chr14	106330168	107048607	-1.0459	109	YES	chr14	106331581	106386891	-9.9249	27	-2.9546
CLL171TD	chr14	106330168	107083152	-0.7604	109	YES	chr14	106330387	106363769	-3.8134	12	-2.9225
CLL175TD	chr14	106330168	107083152	-2.5047	110	YES	chr14	106329377	106478456	-9.7055	38	-3.5220
CLL272TD	chr14	106330168	107081716	-0.9841	111	YES	chr14	106331581	106380172	-9.9266	21	-2.9896
CLL321TD	chr14	106330168	107169498	-0.7248	117	YES	chr14	106354362	106518770	-1.4273	32	-1.9369
CLL005TD	chr14	106330168	107182803	-0.7506	120	YES	chr14	106331373	106370309	-9.2759	18	-3.0124
CLL182TD	chr14	106330168	107218278	-0.8158	125	YES	chr14	106331373	106380172	-9.8129	23	-2.9438
CLL170TD	chr14	106330168	107210901	-0.7360	125	YES	chr14	106330760	106370309	-9.9163	18	-3.1611
CLL152TD	chr14	106330168	107287646	-0.6983	134	YES	chr14	106330760	106369421	-5.9502	18	-2.8870
CLL194TD	chr14	106331756	106345551	-4.2298	0	NO						
CLL117TD	chr14	106331756	106345551	-3.5884	0	YES	chr14	106330760	106386891	-8.6995	26	-2.9833
CLL146TD	chr14	106331756	106345551	-3.4795	0	NO						
CLL063TD	chr14	106331756	106345551	-3.8480	0	YES	chr14	106330760	106360313	-6.0694	12	-2.8341
CLL082TD	chr14	106331756	106345551	-3.3155	0	YES	chr14	106330387	106816090	-8.1022	71	-4.2468
CLL054TD	chr14	106331756	106345551	-3.7211	0	YES	chr14	106330760	106351843	-7.6209	7	-3.0294
CLL166TD	chr14	106331756	106345551	-2.2126	0	YES	chr14	106330760	106380172	-9.9538	24	-3.0116
CLL270TD	chr14	106331756	106345551	-4.5472	0	YES	chr14	106331373	106370493	-9.9659	16	-3.0874
CLL030TD	chr14	106331756	106345551	-3.1174	0	YES	chr14	106331373	106376217	-3.5610	18	-2.7810
CLL319TD	chr14	106331756	106345551	-2.6057	1	YES	chr14	106331373	106361449	-10.0000	12	-3.0189
CLL155TD	chr14	106331756	106345551	-3.7543	1	YES	chr14	106330760	106360313	-10.0000	13	-3.0219
CLL044TD	chr14	106331756	106345551	-4.4615	1	YES	chr14	106330760	106369421	-7.1372	17	-2.9033
CLL267TD	chr14	106331756	106345551	-2.5360	1	YES	chr14	106330387	106357506	-4.1548	12	-2.6611
CLL168TD	chr14	106331756	106481450	-0.6559	33	NO						
CLL136TD	chr14	106331756	106826216	-1.8593	66	YES	chr14	106330387	106357506	-4.4873	8	-2.6532
CLL194TD	chr14	106354195	106453838	-0.6072	18	YES	chr14	106349710	106366443	-7.1575	8	-3.2879
CLL141TD	chr14	106354195	106398480	-1.1591	19	YES	chr14	106329987	106375713	-9.9491	20	-3.1210
CLL319TD	chr14	106354195	106405903	-0.4703	21	YES	chr14	106331373	106361449	-10.0000	12	-3.0189
CLL117TD	chr14	106354195	106398480	-1.2201	21	YES	chr14	106330760	106386891	-8.6995	26	-2.9833
CLL155TD	chr14	106354195	106411088	-0.5768	21	YES	chr14	106330760	106360313	-10.0000	13	-3.0219
CLL054TD	chr14	106354195	106411088	-0.5840	23	YES	chr14	106354362	106405971	-1.2861	23	-1.9737
CLL277TD	chr14	106354195	106410678	-0.9011	23	YES	chr14	106329987	106378065	-9.7456	25	-2.9875
CLL018TD	chr14	106354195	106453838	-0.6492	23	NO						
CLL082TD	chr14	106354195	106481450	-1.1005	27	YES	chr14	106330387	106816090	-8.1022	71	-4.2468
CLL032TD	chr14	106354195	106516175	-0.6502	28	YES	chr14	106330387	106379028	-6.9021	21	-2.9824

CLL146TD	chr14	106354195	106517682	-0.5970	28	YES	chr14	106346841	106357506	-9.1811	7	-3.0148
CLL276TD	chr14	106370629	106453838	-0.4948	13	NO						
CLL100TD	chr14	106483522	106786127	-2.4305	35	YES	chr14	106368457	106780862	-4.2407	57	-4.3246
CLL117TD	chr14	106485479	106497345	-3.6183	2	YES	chr14	106388010	106539432	-4.9691	15	-5.3148
CLL082TD	chr14	106485479	106498648	-4.1903	2	YES	chr14	106330387	106816090	-8.1022	71	-4.2468
CLL141TD	chr14	106485479	106979576	-3.0341	62	YES	chr14	106376217	107042562	-7.7008	84	-5.1246
CLL007TD	chr14	106519396	106724962	-0.6403	23	YES	chr14	106518370	106714578	-0.5846	25	-1.6552
CLL266TD	chr14	106531357	106552225	-2.3623	2	NO						
CLL053TD	chr14	106531357	106552225	-2.3849	2	NO						
CLL044TD	chr14	106531357	106552225	-2.2968	2	NO						
CLL083TD	chr14	106531357	106554165	-2.1660	4	NO						
CLL172TD	chr14	106540426	106561323	-0.7022	2	NO						
CLL006TD	chr14	106601912	106691076	-0.8057	10	YES	chr14	106373023	106667950	-0.7320	41	-1.9870
CLL038TD	chr14	106632143	106779188	-0.5882	15	YES	chr14	106586106	106791373	-0.6606	25	-1.9891
CLL082TD	chr14	106636501	106697473	-2.5987	8	YES	chr14	106330387	106816090	-8.1022	71	-4.2468
CLL082TD	chr14	106712288	106723316	-3.7927	1	YES	chr14	106330387	106816090	-8.1022	71	-4.2468
CLL090TD	chr14	106726871	106829408	-0.5597	11	YES	chr14	106725171	106805561	-0.9174	12	-1.6604
CLL174TD	chr14	106726871	107012899	-0.7352	35	YES	chr14	106733114	107034699	-0.9403	38	-2.8728
CLL082TD	chr14	106785737	106826216	-4.4127	6	YES	chr14	106330387	106816090	-8.1022	71	-4.2468
CLL100TD	chr14	106825816	107150851	-0.7333	43	YES	chr14	106790974	107169901	-0.9732	48	-3.0571
CLL136TD	chr14	106829008	107041398	-0.7250	28	YES	chr14	106829563	107034699	-0.9639	27	-3.6669
CLL082TD	chr14	106833210	106877502	-0.7007	7	YES	chr14	106829563	106866773	-1.1196	9	-3.5838
CLL173TD	chr14	106835238	107060144	-0.5668	29	YES	chr14	106829563	107042562	-1.0421	29	-3.4730
CLL189TD	chr14	106927167	107169498	-0.3894	30	YES	chr14	106926160	107013314	-0.4216	15	-2.6262
CLL141TD	chr14	107010741	107044950	-3.1320	5	YES	chr14	106376217	107042562	-7.7008	84	-5.1246
CLL280TD	chr14	107013844	107081716	-0.8741	9	YES	chr14	107012911	107283143	-0.3559	31	-1.6800
CLL141TD	chr14	107046530	107128311	-1.0109	12	YES	chr14	107048641	107283143	-1.1519	27	-2.0172
CLL172TD	chr14	107050074	107083152	-0.7786	4	YES	chr14	107048641	107083225	-0.8724	7	-3.0084
CLL175TD	chr14	107086213	107128311	-0.8322	4	NO						
CLL053TD	chr14	107137818	107169498	-0.6778	0	NO						
CLL141TD	chr14	107137818	107147214	-5.1626	0	YES	chr14	107048641	107283143	-1.1519	27	-2.0172
CLL044TD	chr14	107151892	107169498	-1.4230	0	YES	chr14	106573203	107178794	-0.8343	79	-2.1425
CLL083TD	chr14	107159767	107181281	-1.4358	4	YES	chr14	107113711	107283143	-0.8322	17	-2.9388
CLL100TD	chr14	107240610	107287646	-0.6596	2	NO						
CLL141TD	chr15	34202339	37462956	-0.5534	158	YES	chr15	34295201	35376969	-0.9415	120	-3.8542
CLL141TD	chr15	40253746	41576166	-1.0220	310	YES	chr15	40253922	41571500	-1.0610	310	-3.5989
CLL279TD	chr15	40530636	40678892	0.2926	59	NO						
CLL192TD	chr15	42110484	42187101	0.2769	101	NO						
CLL100TD	chr15	50651053	51058342	-0.9131	83	YES	chr15	50731262	51204250	-0.8320	84	-3.5269
CLL117TD	chr15	51781915	51791893	0.7697	2	NO						
CLL141TD	chr15	56332487	56599346	-0.9313	9	YES	chr15	56385501	56436579	-1.0077	8	-7.3797
CLL175TD	chr15	65368825	65478380	-0.8670	20	YES	chr15	65410996	65471184	-0.9515	19	-4.4094
CLL279TD	chr15	74460995	74737733	0.3025	63	NO						
CLL145TD	chr16	49962692	50875648	-0.7276	107	YES	chr16	50059526	51098366	-0.7230	108	-3.0494
CLL192TD	chr17	72804	18537304	-0.6050	3102	YES	chr17	5967	2584999	-1.2569	376	-3.2789
CLL145TD	chr17	4579986	4912490	-0.6841	188	YES	chr17	4585772	4910709	-0.7493	187	-2.6673
CLL145TD	chr17	7186117	7258392	-0.6833	76	YES	chr17	7186501	7256220	-0.8010	76	-2.5775
CLL192TD	chr17	18752453	22028853	0.4425	292	YES	chr17	19400745	21821981	0.4045	212	2.5914
CLL048TD	chr17	42214792	42531465	-0.3634	116	NO						
CLL023TD	chr17	44165526	81027191	0.3915	4296	YES	chr17	44336902	45699089	0.5388	135	3.4832
CLL022TD	chr18	14116	14978275	-0.8543	826	YES	chr18	47434	3277211	-0.8853	235	-4.0345
CLL027TD	chr18	485853	542045	-0.5756	0	NO						
CLL027TD	chr18	47072311	47143476	-0.6025	10	NO						
CLL100TD	chr18	68818906	78015254	-0.8682	218	YES	chr18	67872788	78005155	-1.0679	220	-3.6304
CLL152TD	chr18	77981438	78015254	-0.8789	1	NO						
CLL027TD	chr18	77983285	78015254	-0.8006	1	NO						
CLL290TD	chr18	77987347	78015254	-0.6080	1	NO						
CLL124TD	chr19	644208	653049	-0.9377	1	NO						
CLL279TD	chr19	22691038	22700504	0.6273	0	NO						
CLL191TD	chr19	37294161	37493102	-0.9718	19	YES	chr19	37309275	37487180	-1.1080	19	-4.6655
CLL275TD	chr19	42216080	42257386	0.4840	7	NO						
CLL043TD	chr19	51882938	59097086	0.4277	1235	YES	chr19	51883675	52364144	0.3666	71	2.6405
CLL152TD	chr19	59069004	59097086	-0.7228	6	NO						
CLL023TD	chr2	16819	48155	-0.5747	1	NO						
CLL152TD	chr2	16819	54684	-0.8368	2	NO						
CLL100TD	chr2	16819	55994	-0.7164	2	NO						
CLL027TD	chr2	16819	48155	-0.8119	2	NO						
CLL017TD	chr2	16819	30432982	0.5429	1891	YES	chr2	41527	30381476	0.5549	1891	3.8323
CLL008TD	chr2	16819	89160333	0.4635	5257	YES	chr2	41527	89160356	0.4833	5258	3.2568
CLL020TD	chr2	16819	89126843	0.5388	5306	YES	chr2	41527	89102306	0.5442	5306	3.6805
CLL022TD	chr2	50461	89160333	0.4951	5272	YES	chr2	41527	25061688	0.5351	1033	3.5597
CLL017TD	chr2	52017346	66917238	0.3384	752	YES	chr2	50883447	66798339	0.3224	765	2.3601
CLL166TD	chr2	89126443	89480685	-0.5831	39	YES	chr2	89156854	89544238	-1.1298	44	-3.4770

CLL159TD	chr2	89129332	89156875	-0.7316	0	NO						
CLL006TD	chr2	89129332	89158640	-0.9009	1	NO						
CLL052TD	chr2	89129332	89160333	-0.5147	2	NO						
CLL041TD	chr2	89129332	89160333	-0.5989	2	NO						
CLL005TD	chr2	89129332	89480685	-0.7712	40	YES	chr2	89160356	89290378	-0.9660	15	-4.2719
CLL029TD	chr2	89132766	89141808	-5.2084	0	NO						
CLL182TD	chr2	89132766	89141808	-4.7602	0	NO						
CLL082TD	chr2	89132766	89156875	-0.8236	0	NO						
CLL178TD	chr2	89132766	89141808	-4.7301	0	NO						
CLL090TD	chr2	89132766	89158640	-2.1664	1	YES	chr2	89156854	89320097	-0.9598	25	-3.6516
CLL173TD	chr2	89132766	89158640	-2.9058	1	YES	chr2	89156854	89161356	-2.5486	6	-5.9544
CLL020TD	chr2	89132766	89158640	-0.9047	1	NO						
CLL045TD	chr2	89132766	89158640	-2.4828	1	NO						
CLL051TD	chr2	89132766	89158640	-0.7133	1	NO						
CLL148TD	chr2	89132766	89158640	-3.2332	1	NO						
CLL019TD	chr2	89132766	89158640	-2.7531	1	NO						
CLL027TD	chr2	89132766	89158640	-0.8243	1	NO						
CLL171TD	chr2	89132766	89158640	-0.7974	1	NO						
CLL157TD	chr2	89132766	89158640	-0.9371	1	NO						
CLL174TD	chr2	89132766	89158640	-0.8230	1	YES	chr2	89156854	89161356	-1.1072	6	-3.2831
CLL023TD	chr2	89132766	89160333	-0.8755	2	YES	chr2	89156854	89161356	-1.1652	6	-3.5646
CLL189TD	chr2	89132766	89160333	-1.5694	2	YES	chr2	89156854	90249067	-0.9460	77	-3.3042
CLL064TD	chr2	89132766	89160333	-3.3915	2	NO						
CLL272TD	chr2	89132766	89160333	-3.9312	2	YES	chr2	89156854	89196972	-3.6236	10	-6.1602
CLL038TD	chr2	89132766	89160333	-0.4787	2	NO						
CLL184TD	chr2	89132766	89160333	-0.6856	2	NO						
CLL276TD	chr2	89132766	89241426	-2.6169	10	NO						
CLL168TD	chr2	89132766	89276358	-1.9190	14	YES	chr2	89156854	89196972	-5.0728	10	-7.0221
CLL009TD	chr2	89132766	89276358	-2.8620	14	YES	chr2	89156854	89290378	-3.2773	17	-7.0406
CLL277TD	chr2	89132766	89276358	-0.8001	14	YES	chr2	89161356	89293214	-0.9857	16	-3.5029
CLL152TD	chr2	89132766	89312731	-0.7458	23	YES	chr2	89160995	89385101	-0.8927	27	-2.9719
CLL280TD	chr2	89132766	89312731	-2.7372	23	YES	chr2	89161356	89327096	-8.2141	22	-7.1460
CLL270TD	chr2	89132766	89312731	-0.8242	23	YES	chr2	89160995	89327096	-1.1610	23	-3.8059
CLL275TD	chr2	89132766	89312731	-0.7727	23	YES	chr2	89161356	89320097	-0.9910	20	-3.6162
CLL146TD	chr2	89132766	89312731	-0.6997	23	YES	chr2	89185051	89320097	-0.7593	19	-3.4230
CLL048TD	chr2	89132766	89441299	-0.7572	33	YES	chr2	89156854	89417220	-1.0894	33	-3.4909
CLL100TD	chr2	89132766	89450569	-2.3855	35	YES	chr2	89160356	89475786	-4.1794	36	-6.4438
CLL170TD	chr2	89132766	89488503	-0.7020	38	YES	chr2	89156854	89512875	-0.9394	39	-3.3298
CLL191TD	chr2	89132766	89475572	-0.7996	38	YES	chr2	89161356	89459703	-1.0099	33	-3.2878
CLL194TD	chr2	89132766	89508208	-2.5227	39	YES	chr2	89160691	89513294	-5.9925	38	-6.6104
CLL278TD	chr2	89132766	89508208	-1.9548	39	YES	chr2	89156854	89327096	-4.2794	27	-6.8116
CLL136TD	chr2	89132766	90265260	-0.5676	83	YES	chr2	89160995	89513294	-0.9438	37	-3.1657
CLL029TD	chr2	89145568	89156875	-4.8194	0	NO						
CLL178TD	chr2	89147191	89156875	-4.7350	0	NO						
CLL182TD	chr2	89147191	89160333	-4.3593	2	NO						
CLL159TD	chr2	89158240	89539015	-1.7080	42	YES	chr2	89160995	89534308	-4.4536	39	-6.3688
CLL082TD	chr2	89158240	90226606	-1.7380	72	YES	chr2	89160691	89568144	-8.8327	43	-6.4135
CLL013TD	chr2	89159933	89234800	-0.9188	9	YES	chr2	89160038	89196972	-0.9390	9	-2.9641
CLL171TD	chr2	89159933	89203473	-2.9553	9	YES	chr2	89160038	89290378	-4.2233	16	-7.4763
CLL186TD	chr2	89159933	89276358	-0.8164	13	YES	chr2	89160038	89309446	-1.0574	21	-3.6263
CLL090TD	chr2	89159933	89312731	-0.5271	22	YES	chr2	89156854	89320097	-0.9598	25	-3.6516
CLL032TD	chr2	89159933	89312731	-0.7068	22	YES	chr2	89160038	89399739	-0.9336	31	-3.1934
CLL321TD	chr2	89159933	89441299	-0.7253	32	YES	chr2	89160356	89319463	-0.9034	21	-3.5256
CLL273TD	chr2	89159933	89508208	-0.7447	38	YES	chr2	89161356	89512875	-1.0439	35	-2.7556
CLL266TD	chr2	89159933	89539015	-0.6806	42	YES	chr2	89160038	89544964	-0.7795	44	-2.7875
CLL272TD	chr2	89163662	89185502	-0.6032	1	YES	chr2	89156854	89196972	-3.6236	10	-6.1602
CLL178TD	chr2	89163662	89186090	-4.2350	2	YES	chr2	89160691	89568144	-9.5138	43	-6.4361
CLL322TD	chr2	89163662	89203473	-3.1443	4	YES	chr2	89161356	89247203	-6.4075	7	-7.8980
CLL020TD	chr2	89163662	89241426	-3.6293	4	YES	chr2	89160995	89196972	-6.9250	6	-7.4900
CLL052TD	chr2	89163662	89232525	-1.9294	4	YES	chr2	89160691	89513294	-2.2623	39	-5.1414
CLL083TD	chr2	89163662	89203473	-3.4564	4	YES	chr2	89160995	89533640	-5.1079	38	-6.3510
CLL175TD	chr2	89163662	89234800	-0.7846	4	YES	chr2	89160038	89246786	-0.9414	10	-3.6095
CLL023TD	chr2	89163662	89214260	-3.2341	4	NO						
CLL279TD	chr2	89163662	89241426	-0.7602	4	YES	chr2	89160995	89196972	-0.6769	6	-3.0212
CLL006TD	chr2	89163662	89203473	-4.0448	4	YES	chr2	89160995	89544964	-5.2305	41	-6.2867
CLL157TD	chr2	89163662	89203473	-3.8841	4	YES	chr2	89161356	89385101	-8.3899	26	-6.6945
CLL184TD	chr2	89163662	89244018	-2.6475	4	YES	chr2	89160356	89196972	-4.7055	8	-7.1251
CLL124TD	chr2	89163662	89276358	-0.7008	8	YES	chr2	89160356	89290378	-0.9793	15	-3.8947
CLL038TD	chr2	89163662	89276358	-1.8013	8	YES	chr2	89160995	89309446	-2.2245	18	-6.0223
CLL192TD	chr2	89163662	89276358	-0.6073	8	YES	chr2	89160356	89292312	-0.7631	17	-3.7334
CLL323TD	chr2	89163662	89276358	-0.6652	8	YES	chr2	89161356	89290378	-0.8738	12	-3.6078
CLL145TD	chr2	89163662	89312731	-0.6859	16	YES	chr2	89160038	89309863	-0.8463	21	-3.1907
CLL022TD	chr2	89163662	89312731	-0.6908	17	YES	chr2	89160995	89320097	-0.9468	21	-3.1892

CLL188TD	chr2	89163662	89312731	-0.8007	17	YES	chr2	89161356	89399319	-0.8845	26	-3.0600
CLL049TD	chr2	89163662	89312731	-2.3126	17	YES	chr2	89161356	89399739	-3.5881	28	-6.0743
CLL054TD	chr2	89163662	89312731	-0.7494	17	YES	chr2	89160995	89345875	-0.9161	26	-3.1440
CLL008TD	chr2	89163662	89312731	-0.6907	17	YES	chr2	89160691	89975667	-0.6300	47	-2.3127
CLL027TD	chr2	89163662	89312731	-2.9040	17	YES	chr2	89161356	89544964	-5.0820	40	-6.2860
CLL282TD	chr2	89163662	89312731	-3.4583	17	YES	chr2	89160691	89417220	-7.7098	32	-6.4613
CLL017TD	chr2	89163662	89430043	-2.9179	27	YES	chr2	89161356	89544964	-5.9335	40	-6.4746
CLL172TD	chr2	89163662	89441299	-0.7525	27	YES	chr2	89160691	89442506	-0.7657	32	-3.0839
CLL042TD	chr2	89163662	89430043	-1.3733	28	YES	chr2	89160356	89417220	-1.9590	32	-5.2045
CLL051TD	chr2	89163662	89430043	-2.3841	28	YES	chr2	89160691	89417220	-3.3980	31	-5.8378
CLL007TD	chr2	89163662	89430043	-0.7962	28	YES	chr2	89160356	89534308	-0.8856	41	-3.0161
CLL053TD	chr2	89163662	89430043	-0.7718	28	YES	chr2	89160995	89417220	-1.0671	30	-3.1783
CLL041TD	chr2	89163662	89450569	-1.6698	30	YES	chr2	89160356	89513294	-2.1924	39	-5.1665
CLL174TD	chr2	89163662	89450569	-2.3986	30	YES	chr2	89185051	89327096	-5.9416	21	-7.0279
CLL144TD	chr2	89163662	89487166	-0.7306	31	YES	chr2	89160356	89533640	-0.9031	38	-3.5036
CLL141TD	chr2	89163662	89450569	-0.8406	31	YES	chr2	89160995	89568144	-0.9846	44	-3.0220
CLL197TD	chr2	89163662	89534288	-0.6896	36	YES	chr2	89160995	89534308	-0.7321	39	-2.9673
CLL290TD	chr2	89163662	89534288	-0.6866	36	YES	chr2	89185051	89533640	-0.8235	36	-3.0235
CLL181TD	chr2	89163662	89539015	-0.7093	36	YES	chr2	89161356	89544238	-0.8486	38	-3.0251
CLL267TD	chr2	89163662	89539015	-0.6202	37	YES	chr2	89160691	89544964	-0.8717	42	-2.8987
CLL155TD	chr2	89163662	89596790	-0.6940	40	YES	chr2	89161356	89568144	-1.2157	41	-3.5311
CLL117TD	chr2	89163662	89602064	-0.6157	41	YES	chr2	89160356	89568144	-0.9329	45	-3.0710
CLL063TD	chr2	89163662	89617070	-0.6622	41	YES	chr2	89160691	89533640	-0.7950	39	-3.0358
CLL043TD	chr2	89167033	89241426	-0.6358	4	NO						
CLL040TD	chr2	89167033	89312731	-0.2702	16	YES	chr2	89160995	89345875	-0.7401	24	-2.7909
CLL274TD	chr2	89185102	89196925	-0.7634	3	YES	chr2	89160356	89196972	-0.8589	8	-2.5221
CLL029TD	chr2	89185690	89196925	-4.0093	2	YES	chr2	89160356	89196972	-2.9727	8	-5.7343
CLL018TD	chr2	89185690	89196925	-0.8194	2	NO						
CLL039TD	chr2	89185690	89196925	-0.6426	2	NO						
CLL030TD	chr2	89185690	89196925	-0.6861	2	NO						
CLL029TD	chr2	89198090	89430043	-0.7328	23	YES	chr2	89246786	89544964	-0.7752	34	-2.5370
CLL272TD	chr2	89199456	89508208	-0.9253	28	YES	chr2	89246786	89513294	-1.1561	30	-3.7656
CLL023TD	chr2	89216786	89480685	-0.6437	28	YES	chr2	89246786	89544964	-1.1567	34	-3.4086
CLL157TD	chr2	89226231	89232525	-4.9603	0	YES	chr2	89161356	89385101	-8.3899	26	-6.6945
CLL322TD	chr2	89234400	89255624	-1.1290	2	YES	chr2	89161356	89247203	-6.4075	7	-7.8980
CLL178TD	chr2	89234400	89255624	-1.8923	2	YES	chr2	89160691	89568144	-9.5138	43	-6.4361
CLL171TD	chr2	89234400	89270536	-1.3903	4	YES	chr2	89160038	89290378	-4.2233	16	-7.4763
CLL020TD	chr2	89243618	89312731	-0.7356	13	YES	chr2	89246786	89544964	-0.8032	35	-2.5707
CLL276TD	chr2	89243618	89312731	-0.5947	13	YES	chr2	89246786	89345454	-1.1372	19	-3.2373
CLL184TD	chr2	89247673	89508208	-0.7063	27	YES	chr2	89246786	89986315	-0.9825	40	-2.7978
CLL322TD	chr2	89266299	89508208	-0.4556	24	YES	chr2	89265748	89513294	-0.7624	28	-2.7642
CLL168TD	chr2	89312331	90265260	-0.2797	59	YES	chr2	89277955	89475786	-0.7927	26	-2.2758
CLL052TD	chr2	89427165	89508208	-0.8408	5	YES	chr2	89160691	89513294	-2.2623	39	-5.1414
CLL027TD	chr2	89427165	89596790	-0.9703	13	YES	chr2	89161356	89544964	-5.0820	40	-6.2860
CLL022TD	chr2	89427165	90253749	0.6324	43	YES	chr2	89326635	90414113	0.6507	58	2.8512
CLL049TD	chr2	89427166	90093173	-0.2987	26	YES	chr2	89416800	89952847	-0.6986	16	-2.0256
CLL157TD	chr2	89427166	90093173	-0.4054	26	YES	chr2	89399319	89568144	-1.3725	17	-2.7942
CLL051TD	chr2	89440899	89456979	-0.6971	2	NO						
CLL017TD	chr2	89440899	89534288	-0.9678	9	YES	chr2	89161356	89544964	-5.9335	40	-6.4746
CLL083TD	chr2	89440899	89598774	-1.0350	13	YES	chr2	89160995	89533640	-5.1079	38	-6.3510
CLL006TD	chr2	89441648	90105711	-1.1940	25	YES	chr2	89160995	89544964	-5.2305	41	-6.2867
CLL182TD	chr2	89450314	89508208	-0.4770	3	YES	chr2	89160691	89513294	-0.9851	39	-3.3092
CLL100TD	chr2	89450569	89508208	-1.0351	3	YES	chr2	89160356	89475786	-4.1794	36	-6.4438
CLL174TD	chr2	89450569	89508208	-1.0538	3	YES	chr2	89399319	89475786	-7.3360	9	-6.3936
CLL041TD	chr2	89450569	89534288	-0.7081	6	YES	chr2	89160356	89513294	-2.1924	39	-5.1665
CLL279TD	chr2	89456579	89539015	-0.6307	7	NO						
CLL005TD	chr2	89485104	90106037	-0.2521	22	YES	chr2	89385101	89568144	-0.9056	18	-2.5695
CLL194TD	chr2	89533889	90093173	-0.6219	17	YES	chr2	89533640	89568144	-0.7773	6	-2.3608
CLL008TD	chr2	89533889	91815879	0.2988	51	YES	chr2	89976153	91963931	0.4251	66	1.6177
CLL159TD	chr2	89596391	90093173	-0.4502	13	NO						
CLL178TD	chr2	89596391	90093173	-0.7364	13	NO						
CLL083TD	chr2	89601665	90093173	-0.3850	13	NO						
CLL275TD	chr2	89987440	90032877	-0.6127	2	NO						
CLL173TD	chr2	89987440	90045019	-0.7312	3	NO						
CLL017TD	chr2	89987440	90093173	-0.5027	7	NO						
CLL174TD	chr2	89987440	90093173	-0.4979	7	NO						
CLL278TD	chr2	90012137	90265260	-0.5540	29	YES	chr2	90192914	90261664	-1.2169	17	-2.3904
CLL052TD	chr2	90013183	90093173	-0.4301	5	NO						
CLL100TD	chr2	90013183	90093173	-0.4963	5	NO						
CLL041TD	chr2	90013183	90093173	-0.4987	5	NO						
CLL189TD	chr2	90044619	90090683	-0.6538	4	YES	chr2	89156854	90249067	-0.9460	77	-3.3042
CLL189TD	chr2	90152212	90265260	-1.0145	16	YES	chr2	89156854	90249067	-0.9460	77	-3.3042
CLL146TD	chr2	90226206	90240890	-1.0300	2	YES	chr2	90218695	90259920	-1.2370	11	-2.6152

CLL273TD	chr2	90226206	90253749	-0.8810	4	YES	chr2	90153708	90259920	-0.9064	17	-1.6899
CLL148TD	chr2	90226206	90253749	-0.7617	4	YES	chr2	90211703	90259920	-0.9871	13	-2.3221
CLL082TD	chr2	90230242	90265260	-0.6953	7	YES	chr2	89952847	90249067	-1.1368	31	-2.4598
CLL290TD	chr2	111391997	112490498	-0.6445	52	YES	chr2	111395518	112252405	-0.6467	52	-3.1365
CLL082TD	chr2	144137253	144323942	-0.8296	5	NO						
CLL279TD	chr2	219313919	219394922	-0.2666	19	NO						
CLL048TD	chr2	232954062	243041505	-0.5994	961	YES	chr2	233410189	240002779	-0.6051	548	-2.9720
CLL038TD	chr20	3987416	4047854	-0.3313	0	NO						
CLL322TD	chr20	6207743	6241610	0.5850	0	NO						
CLL020TD	chr20	14781213	14826157	-1.3738	0	YES	chr20	68319	17596034	-0.6355	976	-2.5639
CLL020TD	chr20	17666114	26193955	-0.4327	471	YES	chr20	17596522	29612290	-0.4681	493	-2.0619
CLL141TD	chr21	15711781	16958298	-0.7604	25	YES	chr21	15745911	16336974	-0.8867	25	-3.6095
CLL141TD	chr21	17168229	17199741	-0.9189	9	NO						
CLL141TD	chr21	24455305	25158740	-0.8499	0	NO						
CLL280TD	chr22	22381553	22722107	-0.8807	22	YES	chr22	22385540	22724128	-0.8774	23	-3.1085
CLL168TD	chr22	22385947	22748867	-0.6599	25	YES	chr22	22385540	22735388	-0.6581	26	-2.4848
CLL173TD	chr22	22517433	22522289	-0.7565	0	NO						
CLL182TD	chr22	22551038	23241689	-0.8108	70	YES	chr22	22556219	22657565	-1.8207	7	-3.2332
CLL278TD	chr22	22713221	23244777	-0.7171	51	YES	chr22	22724128	22975934	-0.8894	22	-3.8973
CLL173TD	chr22	22724404	22763326	-0.7364	4	NO						
CLL280TD	chr22	22724404	23247059	-2.8660	47	YES	chr22	22730538	23241756	-9.1392	46	-7.9127
CLL136TD	chr22	22733651	23234829	-0.6759	42	YES	chr22	22735388	23223249	-0.8792	42	-4.0428
CLL045TD	chr22	22736444	23234829	-0.7498	42	YES	chr22	22735388	23235817	-0.9112	44	-3.6092
CLL064TD	chr22	22736444	23247059	-0.8703	47	YES	chr22	22735388	23247126	-1.0508	49	-3.6034
CLL090TD	chr22	22736444	23247059	-0.8489	50	YES	chr22	22735388	23165257	-1.0235	44	-3.6300
CLL168TD	chr22	22749794	23247059	-1.8130	47	YES	chr22	22749500	23247126	-2.6228	48	-6.2688
CLL173TD	chr22	22765216	23247059	-2.4999	45	YES	chr22	22782035	22906026	-9.0236	12	-10.2824
CLL189TD	chr22	22825827	23247059	-0.5367	43	YES	chr22	22735388	23114750	-0.4651	36	-2.2611
CLL042TD	chr22	23026882	23246029	-0.3974	23	YES	chr22	22984803	23264746	-0.3468	34	-2.0362
CLL006TD	chr22	23041645	23054698	-0.6992	1	NO						
CLL020TD	chr22	23041645	23241689	-0.8852	19	YES	chr22	23046981	23241756	-0.9664	20	-3.1815
CLL029TD	chr22	23056362	23093788	-0.8552	3	NO						
CLL100TD	chr22	23056362	23234829	-0.7863	15	NO						
CLL019TD	chr22	23056362	23240427	-0.7890	17	YES	chr22	23055479	23241756	-0.6726	19	-3.1363
CLL009TD	chr22	23056362	23247059	-0.8457	18	YES	chr22	23063315	23247126	-0.8632	19	-2.7997
CLL006TD	chr22	23056362	23247059	-3.0147	18	YES	chr22	23063315	23247126	-5.7736	19	-6.7538
CLL178TD	chr22	23062667	23092724	-0.8668	3	NO						
CLL082TD	chr22	23064017	23239061	-0.7084	16	YES	chr22	23077248	23241756	-1.0199	17	-3.8283
CLL272TD	chr22	23085408	23241689	-0.7889	15	YES	chr22	23077248	23241756	-0.9569	17	-3.3931
CLL029TD	chr22	23094250	23234829	-3.2471	12	YES	chr22	23101171	23223249	-7.0442	12	-6.7989
CLL178TD	chr22	23094250	23241689	-2.7407	14	YES	chr22	23154453	23223249	-10.0000	7	-6.8542
CLL018TD	chr22	23103564	23247059	-0.8098	13	YES	chr22	23101171	23237534	-0.9201	14	-3.0871
CLL041TD	chr22	23103564	23244777	-1.7194	13	YES	chr22	23114750	23241756	-2.5279	13	-5.7025
CLL146TD	chr22	23108423	23234829	-2.4126	10	YES	chr22	23114750	23235817	-6.8565	11	-7.2343
CLL276TD	chr22	23138710	23247059	-0.6702	10	YES	chr22	23135163	23247126	-0.8349	12	-2.6336
CLL032TD	chr22	23138710	23258429	-0.6904	13	YES	chr22	23135163	23256401	-0.6361	14	-2.8456
CLL194TD	chr22	23165575	23241689	-0.7516	6	YES	chr22	23165454	23241756	-1.0530	7	-3.7821
CLL152TD	chr22	23165575	23247059	-0.8326	7	NO						
CLL148TD	chr22	23224126	23234829	-0.9807	0	NO						
CLL146TD	chr22	23238661	23247059	-0.6000	1	NO						
CLL152TD	chr22	42294616	42371108	-0.5282	16	NO						
CLL064TD	chr22	47218653	51224402	-0.9280	383	YES	chr22	47243690	51237147	-1.1043	384	-2.4403
CLL023TD	chr3	61875	5848545	-0.7837	205	YES	chr3	361444	4403822	-0.7572	110	-3.5738
CLL141TD	chr3	47031601	48917211	-1.0435	496	YES	chr3	47030690	48921373	-1.1265	499	-3.4846
CLL174TD	chr3	47416540	47522517	-0.8446	32	YES	chr3	47437579	47476412	-1.0411	31	-2.9290
CLL155TD	chr3	47635955	47644751	-0.7684	0	NO						
CLL174TD	chr3	47851098	49978951	-0.8177	692	YES	chr3	47852127	48450938	-0.9519	92	-4.1059
CLL174TD	chr3	52966182	53102112	-0.8415	5	NO						
CLL275TD	chr3	60240257	60279230	0.3902	0	NO						
CLL039TD	chr3	60870865	60939318	-0.8583	1	NO						
CLL048TD	chr3	118985758	197840480	0.4404	4761	YES	chr3	118945621	122591232	0.3961	401	3.3587
CLL100TD	chr3	122827975	197840480	0.5264	4435	YES	chr3	123213715	125735525	0.4992	245	3.5786
CLL082TD	chr3	143106361	143252946	-0.8052	6	YES	chr3	143185835	143236876	-0.8840	6	-3.7155
CLL027TD	chr3	150511474	150567846	-0.5595	0	NO						
CLL045TD	chr3	196504448	196551297	-0.3620	12	NO						
CLL267TD	chr4	421075	479412	-0.7301	2	YES	chr4	420216	514821	-0.3361	9	-2.2863
CLL178TD	chr4	29141262	29784750	-0.8663	1	NO						
CLL100TD	chr4	39322462	39377923	-0.6240	7	NO						
CLL038TD	chr4	151564016	151597599	-0.3597	0	NO						
CLL027TD	chr4	151570424	151597599	-0.5820	0	NO						
CLL009TD	chr4	171423540	190916819	-0.7784	566	YES	chr4	171525921	190882911	-0.8190	562	-3.7661
CLL136TD	chr5	20695683	20939003	-0.3266	0	NO						
CLL136TD	chr5	34069555	34402627	-0.3432	9	NO						

CLL168TD	chr5	34069555	34402627	-0.4189	9	NO						
CLL279TD	chr5	34069555	34480071	-0.4293	9	NO						
CLL152TD	chr5	78450787	78508370	-0.8234	0	NO						
CLL064TD	chr5	78464596	78502685	-0.6047	0	NO						
CLL027TD	chr5	108643441	108698690	-0.4339	4	NO						
CLL023TD	chr5	133913776	133982166	-0.6022	7	NO						
CLL173TD	chr6	296474	381523	-0.3413	7	NO						
CLL178TD	chr6	387408	50073545	0.4160	4414	YES	chr6	395830	32427550	0.4234	2299	2.8874
CLL152TD	chr6	18288883	18359933	-0.4571	0	NO						
CLL027TD	chr6	51827829	51865122	-0.5130	0	NO						
CLL048TD	chr6	62414996	62479221	0.6760	1	NO						
CLL082TD	chr6	62745557	62873715	-0.8453	1	NO						
CLL049TD	chr6	79892342	120465184	-0.7504	1725	YES	chr6	79911333	90305623	-0.8027	471	-3.7545
CLL152TD	chr6	101171890	101212177	-0.4114	1	NO						
CLL019TD	chr7	40062122	40144404	-0.8148	10	YES	chr7	40085409	40133673	-0.9796	10	-4.1262
CLL152TD	chr7	71559725	71589033	-0.7524	1	NO						
CLL027TD	chr7	71559725	71597824	-0.6152	1	NO						
CLL100TD	chr7	71568404	71585893	-0.6700	1	NO						
CLL144TD	chr7	85594936	85741498	0.5414	0	NO						
CLL178TD	chr7	114334126	133589746	-0.8731	981	YES	chr7	114563472	133580299	-0.9634	981	-3.7746
CLL152TD	chr7	131843507	131899241	-0.5483	16	NO						
CLL017TD	chr7	156792750	156803343	-0.7515	3	NO						
CLL152TD	chr7	159097982	159128663	-1.0208	0	NO						
CLL290TD	chr8	161272	29030957	-0.7051	1532	YES	chr8	142120	7056066	-0.7127	230	-3.3682
CLL043TD	chr8	176293	41305627	-0.6845	2125	YES	chr8	142120	9634112	-0.8545	303	-3.3608
CLL282TD	chr8	176654	34630057	-0.8628	1705	YES	chr8	163525	7005679	-0.9576	227	-3.9727
CLL282TD	chr8	34638882	43388433	0.4766	637	YES	chr8	35383169	43415755	0.4476	638	3.1944
CLL009TD	chr8	39274937	39381262	-0.6073	0	NO						
CLL282TD	chr8	47777490	56161593	-0.8713	277	YES	chr8	47886143	56015292	-0.9481	277	-4.5790
CLL178TD	chr8	56882676	86550838	0.5108	930	YES	chr8	56879244	67062402	0.4892	261	3.5577
CLL064TD	chr8	59529447	144292596	0.5558	3155	YES	chr8	59515736	67341285	0.5497	191	4.2217
CLL290TD	chr8	92935342	146239365	0.4850	2470	YES	chr8	92972427	97172447	0.4605	220	3.4571
CLL145TD	chr8	110274049	146294242	0.4730	1670	YES	chr8	110283176	143559513	0.5518	1217	4.0530
CLL009TD	chr8	128208374	128247684	0.7057	0	NO						
CLL152TD	chr8	146261357	146294242	-0.8639	5	NO						
CLL083TD	chr9	70984281	77075175	-0.7101	274	YES	chr9	70484362	75906141	-0.7681	278	-3.4753
CLL279TD	chr9	74571577	74591097	-0.7701	2	NO						
CLL186TD	chr9	95142442	95204491	-0.8971	9	YES	chr9	95147866	95178890	-0.8864	9	-4.2291
CLL146TD	chr9	129604944	129644649	-0.4413	1	NO						
CLL048TD	chr9	129620665	132552492	-0.2879	643	NO						
CLL100TD	chr9	129623945	129657519	-0.6836	1	NO						
