# Supplementary material for: Estimation of Copy Number Alterations from Exome Sequencing Data
Source: PLoS One. 2012 Dec 19;7(12):e51422. doi: 10.1371/journal.pone.0051422 (PMC3526607; doi:10.1371/journal.pone.0051422)
Supplement: Table S2 — Comparison of somatic CNAs detected by exome2cnv vs. aCGH to determine false discovery rate. (PDF) [file pone.0051422.s002.pdf]

EXOME							aCGH							
CASE	CHROM	START	END	LOG2RATIO	# EXONS	StdDev	FOUND	CHROM	START	END	LOG2RATIO	# EXONS	COMMENTS	
CLL274TD	chr1	7700386	7792463	0.9364	6	1.7122	NO							
CLL006TD	chr1	12081780	12091731	0.7815	8	1.7661	NO							
CLL082TD	chr1	22150070	22154323	-1.2911	6	-1.7311	NO							
CLL191TD	chr1	40041376	40205825	0.4663	15	1.6521	NO							
CLL191TD	chr1	40773800	40778203	0.5203	8	2.3475	NO							
CLL145TD	chr1	41578866	43282098	-0.8159	110	-3.3368	YES	chr1	41572891	43287342	-0.7145	110		
CLL148TD	chr1	46086934	46088404	0.5899	6	1.6053	NO							
CLL155TD	chr1	78432316	78444463	-0.8221	8	-3.1091	YES	chr1	78433832	78452941	-0.8181	4		
CLL189TD	chr1	109808298	109814813	-0.3676	11	-1.7640	NO							
CLL157TD	chr1	150676571	150684258	-0.4427	7	-1.8885	NO							
CLL082TD	chr1	227171233	227172907	-0.7919	6	-2.2318	NO							
CLL170TD	chr1	230410130	230492613	-0.5019	8	-2.1555	NO							
CLL049TD	chr10	102675717	106214143	-0.8608	638	-3.2902	YES	chr10	102641828	106419703	-0.7237	638		
CLL141TD	chr10	103649117	104176108	-1.0251	133	-3.9548	YES	chr10	103634312	104177223	-1.0034	133		
CLL064TD	chr10	104160694	104196244	-0.6812	25	-1.9207	YES	chr10	104161255	104192055	-0.7374	22		
CLL178TD	chr10	104160862	104852860	-0.8987	126	-2.9443	YES	chr10	104159664	104853373	-0.7501	130		
CLL178TD	chr10	105036937	105093635	-0.8543	7	-3.1110	YES	chr10	105007763	105100437	-0.8027	7		
CLL184TD	chr11	43333651	45277209	-0.9153	140	-3.4682	YES	chr11	42655597	45369571	-0.7617	140		
CLL110TD	chr11	61551299	61557929	-0.8842	6	-1.8015	NO							
CLL275TD	chr11	67262471	67265981	0.9377	6	2.0489	NO							
CLL275TD	chr11	72423162	72469536	0.7485	6	1.7023	NO							
CLL042TD	chr11	76956316	116656464	-0.4984	1805	-2.9969	YES	chr11	76513928	117661843	-0.4171	2028		
CLL023TD	chr11	77300986	77924655	-1.0005	80	-3.5747	YES	chr11	77179449	117609214	-0.8262	1961		
CLL023TD	chr11	77930269	102953959	-0.8191	893	-3.8962	YES	chr11	77179449	117609214	-0.8262	1961		
CLL006TD	chr11	78270534	89449046	-0.9866	331	-3.9747	YES	chr11	78240712	116777756	-0.9000	1734		
CLL290TD	chr11	82533422	89608133	-0.9468	311	-3.8275	YES	chr11	79590720	116062438	-0.8491	1643		
CLL054TD	chr11	86055583	114578256	-0.9208	1462	-3.8902	YES	chr11	86053929	114937176	-0.7975	1462		
CLL006TD	chr11	89595601	89774166	-1.2697	10	-1.8505	YES	chr11	78240712	116777756	-0.9000	1734		
CLL290TD	chr11	89719169	115110946	-0.9575	1328	-4.2622	YES	chr11	79590720	116062438	-0.8491	1643		
CLL006TD	chr11	89867907	103022819	-0.9991	542	-4.5365	YES	chr11	78240712	116777756	-0.9000	1734		
CLL178TD	chr11	92523066	102398480	-0.8893	400	-4.3660	YES	chr11	92513723	133254506	-0.8701	2738		
CLL041TD	chr11	94800334	111941112	-0.6348	834	-3.0826	YES	chr11	94769441	123056760	-0.6585	1930		
CLL278TD	chr11	99690199	116747578	-0.9151	1061	-4.0853	YES	chr11	96159104	116753164	-0.8110	1063		
CLL184TD	chr11	101324358	103027067	-0.8296	178	-4.1564	YES	chr11	101194338	117964476	-0.5119	1171		
CLL145TD	chr11	101761989	134320539	-0.8880	2517	-3.7222	YES	chr11	101389939	134945306	-0.7980	2517		
CLL178TD	chr11	102449761	104896973	-0.8676	202	-4.0423	YES	chr11	92513723	133254506	-0.8701	2738		
CLL023TD	chr11	102959911	103156929	-0.6066	76	-3.5964	YES	chr11	77179449	117609214	-0.8262	1961		
CLL006TD	chr11	103026024	116767850	-0.9676	833	-4.0744	YES	chr11	78240712	116777756	-0.9000	1734		
CLL184TD	chr11	103033756	106558228	-0.8164	141	-4.1246	YES	chr11	101194338	117964476	-0.5119	1171		
CLL023TD	chr11	103158237	110501282	-0.7949	352	-3.8702	YES	chr11	77179449	117609214	-0.8262	1961		
CLL178TD	chr11	104903761	107686483	-0.9149	107	-4.1996	YES	chr11	92513723	133254506	-0.8701	2738		
CLL017TD	chr11	105836610	128564063	-0.9707	1914	-3.7459	YES	chr11	105820665	128506612	-0.8830	1913		
CLL063TD	chr11	106558228	108122540	-1.0001	128	-4.2278	YES	chr11	106420522	108122884	-0.8464	128		
CLL178TD	chr11	107832644	110482011	-0.9038	186	-3.9846	YES	chr11	92513723	133254506	-0.8701	2738		
CLL038TD	chr11	109294356	113779976	-0.7561	338	-3.2607	YES	chr11	109139541	113781239	-0.6921	338		
CLL063TD	chr11	110306499	115110946	-0.9450	388	-3.9442	YES	chr11	110211047	116153447	-0.8626	388		
CLL178TD	chr11	110485266	124543510	-0.9933	1347	-3.7972	YES	chr11	92513723	133254506	-0.8701	2738		
CLL023TD	chr11	110561220	116658158	-0.9118	395	-3.7956	YES	chr11	77179449	117609214	-0.8262	1961		
CLL041TD	chr11	111941704	117089142	-0.7522	331	-3.1063	YES	chr11	94769441	123056760	-0.6585	1930		
CLL184TD	chr11	112832058	117947958	-0.9237	408	-3.1459	YES	chr11	101194338	117964476	-0.5119	1171		
CLL042TD	chr11	116657165	117306287	-0.3421	141	-2.5903	YES	chr11	76513928	117661843	-0.4171	2028		
CLL023TD	chr11	116660833	117403013	-1.1238	165	-3.1420	YES	chr11	77179449	117609214	-0.8262	1961		
CLL041TD	chr11	117094742	117691501	-0.8020	89	-2.6719	YES	chr11	94769441	123056760	-0.6585	1930		
CLL041TD	chr11	117695320	123065544	-0.7651	671	-3.0469	YES	chr11	94769441	123056760	-0.6585	1930		
CLL178TD	chr11	124545087	132812783	-0.9204	487	-3.5714	YES	chr11	92513723	133254506	-0.8701	2738		
CLL277TD	chr12	73256	6438371	0.4235	590	2.5946	YES	chr12	163393	34756350	0.4735	2822		
CLL032TD	chr12	87509	7343411	0.5589	965	3.0818	YES	chr12	191809	34756350	0.4431	2789		
CLL275TD	chr12	148430	346239	0.7021	36	3.3612	YES	chr12	163393	34756350	0.5169	2808		
CLL064TD	chr12	148430	645362	0.4080	81	3.0082	YES	chr12	189361	34417592	0.5136	2804		
CLL282TD	chr12	148430	6125206	0.5052	525	3.1675	YES	chr12	194049	34756350	0.5133	2789		
CLL166TD	chr12	148430	9596082	0.5140	1354	3.2107	YES	chr12	250702	34360180	0.4152	2810		
CLL005TD	chr12	148430	48191104	0.5231	3471	3.5899	YES	chr12	163393	34533264	0.5302	2821		
CLL045TD	chr12	148999	3149497	0.4568	352	2.6347	YES	chr12	189361	34756350	0.4822	2805		
CLL148TD	chr12	278134	347073	0.8518	26	3.1842	YES	chr12	189361	34756350	0.4976	2776		
CLL082TD	chr12	332288	6861035	0.4501	740	2.5129	YES	chr12	191809	34756350	0.5293	2769		
CLL275TD	chr12	347073	1599207	0.4713	134	3.1065	YES	chr12	163393	34756350	0.5169	2808		
CLL148TD	chr12	351729	550892	0.3805	42	3.3197	YES	chr12	189361	34756350	0.4976	2776		
CLL148TD	chr12	644293	772458	0.7710	27	3.0650	YES	chr12	189361	34756350	0.4976	2776		
CLL064TD	chr12	653492	2921914	0.5311	219	3.2189	YES	chr12	189361	34417592	0.5136	2804		
CLL148TD	chr12	862660	1023557	0.4565	34	3.7551	YES	chr12	189361	34756350	0.4976	2776		
CLL148TD	chr12	1025775	4397961	0.6344	260	3.3222	YES	chr12	189361	34756350	0.4976	2776		
CLL275TD	chr12	1675858	7344110	0.6354	807	3.9726	YES	chr12	163393	34756350	0.5169	2808		
CLL064TD	chr12	2926376	3149228	0.3363	53	2.3844	YES	chr12	189361	34417592	0.5136	2804		
CLL064TD	chr12	3149497	6130983	0.5811	180	3.8526	YES	chr12	189361	34417592	0.5136	2804		
CLL045TD	chr12	3310330	57596111	0.4915	5249	3.0283	YES	chr12	189361	34756350	0.4822	2805		
CLL148TD	chr12	4408980	4835636	0.4218	63	2.9891	YES	chr12	189361	34756350	0.4976	2776		

CLL148TD	chr12	4848316	6090904	0.6120	54	3.9517	YES	chr12	189361	34756350	0.4976	2776	
CLL148TD	chr12	6103018	6928389	0.6494	269	3.3788	YES	chr12	189361	34756350	0.4976	2776	
CLL282TD	chr12	6125668	8328392	0.4419	584	3.0589	YES	chr12	194049	34756350	0.5133	2789	
CLL064TD	chr12	6132754	9010499	0.4223	676	2.8543	YES	chr12	189361	34417592	0.5136	2804	
CLL277TD	chr12	6439204	9586653	0.4334	776	2.5196	YES	chr12	163393	34756350	0.4735	2822	
CLL148TD	chr12	6932861	6952921	1.0007	27	3.4516	YES	chr12	189361	34756350	0.4976	2776	
CLL148TD	chr12	6954707	7031182	0.6965	49	3.6571	YES	chr12	189361	34756350	0.4976	2776	
CLL148TD	chr12	7031475	7075016	1.0131	30	3.4866	YES	chr12	189361	34756350	0.4976	2776	
CLL148TD	chr12	7075563	7479511	0.6209	99	3.7228	YES	chr12	189361	34756350	0.4976	2776	
CLL082TD	chr12	7342896	9392014	0.564	346	3.7164	YES	chr12	191809	34756350	0.529285	2769	
CLL032TD	chr12	7343767	9554990	0.4331	360	3.7079	YES	chr12	191809	34756350	0.443094	2789	
CLL275TD	chr12	7351567	30906217	0.4951	1652	2.9918	YES	chr12	163393	34756350	0.51692	2808	
CLL148TD	chr12	7480865	9980122	0.4811	377	3.1391	YES	chr12	189361	34756350	0.497552	2776	
CLL282TD	chr12	8329466	48119078	0.5237	2283	3.7832	YES	chr12	194049	34756350	0.5133	2789	
CLL064TD	chr12	9013453	9596082	0.5870	139	4.1324	YES	chr12	189361	34417592	0.5136	2804	
CLL032TD	chr12	9571220	9728204	0.7315	45	1.8387	YES	chr12	191809	34756350	0.4431	2789	
CLL277TD	chr12	9726035	50474278	0.4501	2606	2.8220	YES	chr12	163393	34756350	0.4735	2822	
CLL064TD	chr12	9728204	19444544	0.5654	570	3.9933	YES	chr12	189361	34417592	0.5136	2804	
CLL032TD	chr12	9747823	31106907	0.4180	1248	4.0678	YES	chr12	191809	34756350	0.4431	2789	
CLL082TD	chr12	9747823	48096426	0.5740	2020	4.4115	YES	chr12	191809	34756350	0.5293	2769	
CLL166TD	chr12	9747823	120802447	0.5088	8316	3.3625	YES	chr12	250702	34360180	0.4152	2810	
CLL148TD	chr12	9984900	10762424	0.3385	116	2.9185	YES	chr12	189361	34756350	0.4976	2776	
CLL148TD	chr12	10763200	15834088	0.4721	330	3.5020	YES	chr12	189361	34756350	0.4976	2776	
CLL148TD	chr12	15835795	27923965	0.3829	644	3.1409	YES	chr12	189361	34756350	0.4976	2776	
CLL064TD	chr12	19593220	31246158	0.5534	683	3.9949	YES	chr12	189361	34417592	0.5136	2804	
CLL148TD	chr12	27933219	31106907	0.4467	137	3.6008	YES	chr12	189361	34756350	0.4976	2776	
CLL275TD	chr12	31106907	31256437	0.8972	31	3.6639	YES	chr12	163393	34756350	0.5169	2808	
CLL032TD	chr12	31116706	31241911	0.8810	12	2.3319	YES	chr12	191809	34756350	0.4431	2789	
CLL148TD	chr12	31116706	31256437	0.8566	29	2.2649	YES	chr12	189361	34756350	0.4976	2776	
CLL032TD	chr12	31242290	48096426	0.4137	758	4.1407	YES	chr12	191809	34756350	0.4431	2789	
CLL064TD	chr12	31247485	33031849	0.4241	150	3.1594	YES	chr12	189361	34417592	0.5136	2804	
CLL148TD	chr12	31265139	48104523	0.4001	744	3.3445	YES	chr12	189361	34756350	0.4976	2776	
CLL275TD	chr12	31265139	48104523	0.4897	745	2.9494	YES	chr12	163393	34756350	0.5169	2808	
CLL064TD	chr12	33529740	48372310	0.5683	688	4.1040	YES	chr12	189361	34417592	0.5136	2804	
CLL032TD	chr12	48104523	48376807	0.6836	87	2.4104	YES	chr12	38453410	133779217	0.4287	8061	
CLL148TD	chr12	48105366	48391439	0.8047	109	3.0607	YES	chr12	38503256	133779217	0.4875	8100	
CLL275TD	chr12	48105366	48391439	0.7325	110	3.8808	YES	chr12	37957940	133779217	0.5145	8151	
CLL282TD	chr12	48131258	65612305	0.4616	2778	3.1073	YES	chr12	37957940	133779217	0.4997	8113	
CLL005TD	chr12	48191856	58194900	0.5623	2470	3.3912	YES	chr12	38453410	133779217	0.5251	8176	
CLL064TD	chr12	48373230	52822344	0.4414	874	3.0000	YES	chr12	38448467	133779217	0.4811	8132	
CLL032TD	chr12	48377134	49314638	0.4524	180	3.4196	YES	chr12	38453410	133779217	0.4287	8061	
CLL082TD	chr12	48379499	49169039	0.4936	125	3.5663	YES	chr12	38448467	133779217	0.5185	8096	
CLL148TD	chr12	48391907	49110227	0.4799	98	3.6482	YES	chr12	38503256	133779217	0.4875	8100	
CLL275TD	chr12	48391907	49317932	0.5068	158	3.6103	YES	chr12	37957940	133779217	0.5145	8151	
CLL148TD	chr12	49162316	50369200	0.7089	339	3.3504	YES	chr12	38503256	133779217	0.4875	8100	
CLL082TD	chr12	49218895	49665982	0.4522	159	2.0693	YES	chr12	38448467	133779217	0.5185	8096	
CLL032TD	chr12	49315772	50513793	0.5960	322	2.8769	YES	chr12	38453410	133779217	0.4287	8061	
CLL275TD	chr12	49318327	50483954	0.6417	312	3.7852	YES	chr12	37957940	133779217	0.5145	8151	
CLL030TD	chr12	50039551	50041935	-0.8900	6	-1.7050	NO						
CLL082TD	chr12	50367685	57492999	0.4694	1582	2.5333	YES	chr12	38448467	133779217	0.5185	8096	
CLL148TD	chr12	50383998	52188109	0.4853	285	3.2868	YES	chr12	38503256	133779217	0.4875	8100	
CLL277TD	chr12	50475289	59267764	0.4264	1965	2.5149	YES	chr12	37957940	133779217	0.4656	8159	
CLL275TD	chr12	50484264	51565994	0.3888	154	2.5723	YES	chr12	37957940	133779217	0.5145	8151	
CLL032TD	chr12	50524252	51585312	0.3929	140	3.4631	YES	chr12	38453410	133779217	0.4287	8061	
CLL275TD	chr12	51584022	55038442	0.6070	787	3.8457	YES	chr12	37957940	133779217	0.5145	8151	
CLL032TD	chr12	51586033	54796889	0.5670	693	2.9012	YES	chr12	38453410	133779217	0.4287	8061	
CLL148TD	chr12	52200039	53708003	0.6961	448	3.1990	YES	chr12	38503256	133779217	0.4875	8100	
CLL064TD	chr12	52824286	53085195	0.6478	100	3.7913	YES	chr12	38448467	133779217	0.4811	8132	
CLL064TD	chr12	53085648	54891534	0.4279	411	2.7799	YES	chr12	38448467	133779217	0.4811	8132	
CLL148TD	chr12	53708493	56620081	0.5901	545	3.6207	YES	chr12	38503256	133779217	0.4875	8100	
CLL032TD	chr12	54797335	54801806	0.3076	11	2.8505	YES	chr12	38453410	133779217	0.4287	8061	
CLL032TD	chr12	54805562	55025974	0.4058	61	3.2768	YES	chr12	38453410	133779217	0.4287	8061	
CLL064TD	chr12	54893133	56090617	0.5704	116	4.2759	YES	chr12	38448467	133779217	0.4811	8132	
CLL032TD	chr12	55038911	56030666	0.3931	43	5.0782	YES	chr12	38453410	133779217	0.4287	8061	
CLL275TD	chr12	55038911	57144780	0.5143	544	3.6654	YES	chr12	37957940	133779217	0.5145	8151	
CLL032TD	chr12	56075487	56086862	0.6792	9	2.5356	YES	chr12	38453410	133779217	0.4287	8061	
CLL032TD	chr12	56089243	56115391	0.8501	17	2.3346	YES	chr12	38453410	133779217	0.4287	8061	
CLL064TD	chr12	56090970	58204765	0.4209	942	2.9698	YES	chr12	38448467	133779217	0.4811	8132	
CLL032TD	chr12	56117631	57537401	0.4676	549	3.2736	YES	chr12	38453410	133779217	0.4287	8061	
CLL148TD	chr12	56622777	56663234	0.7942	41	3.7803	YES	chr12	38503256	133779217	0.4875	8100	
CLL148TD	chr12	56663897	56716831	0.3915	26	2.4518	YES	chr12	38503256	133779217	0.4875	8100	
CLL148TD	chr12	56717071	57037158	0.6182	141	3.9448	YES	chr12	38503256	133779217	0.4875	8100	
CLL148TD	chr12	57037440	57317575	0.3784	36	2.8113	YES	chr12	38503256	133779217	0.4875	8100	
CLL275TD	chr12	57145910	57435188	0.6657	36	4.6738	YES	chr12	37957940	133779217	0.5145	8151	
CLL148TD	chr12	57323105	57432572	0.7522	27	4.2480	YES	chr12	38503256	133779217	0.4875	8100	
CLL148TD	chr12	57432966	57466512	0.4508	22	2.8803	YES	chr12	38503256	133779217	0.4875	8100	
CLL275TD	chr12	57436808	57535105	0.4981	42	3.6044	YES	chr12	37957940	133779217	0.5145	8151	
CLL148TD	chr12	57472314	57543427	0.7364	27	3.1541	YES	chr12	38503256	133779217	0.4875	8100	

CLL275TD	chr12	57537401	57593583	0.6493	55	3.7761	YES	chr12	37957940	133779217	0.5145	8151	
CLL032TD	chr12	57538698	57637819	0.7392	94	2.1750	YES	chr12	38453410	133779217	0.4287	8061	
CLL148TD	chr12	57547951	57630749	0.9000	88	3.0793	YES	chr12	38503256	133779217	0.4875	8100	
CLL275TD	chr12	57594181	57625243	0.8744	28	4.1649	YES	chr12	37957940	133779217	0.5145	8151	
CLL045TD	chr12	57598102	110815189	0.5003	3148	3.2116	YES	chr12	38503256	133779217	0.4753	8112	
CLL275TD	chr12	57625445	58335599	0.5804	308	3.8402	YES	chr12	37957940	133779217	0.5145	8151	
CLL148TD	chr12	57637553	59271167	0.6453	303	3.5370	YES	chr12	38503256	133779217	0.4875	8100	
CLL032TD	chr12	57638272	58335599	0.5338	291	3.1233	YES	chr12	38453410	133779217	0.4287	8061	
CLL082TD	chr12	57651726	58209730	0.4542	270	2.3052	YES	chr12	38448467	133779217	0.5185	8096	
CLL005TD	chr12	58195886	123466341	0.5144	4295	3.5227	YES	chr12	38453410	133779217	0.5251	8176	
CLL064TD	chr12	58206995	68946715	0.5191	401	3.7617	YES	chr12	38448467	133779217	0.4811	8132	
CLL082TD	chr12	58223154	108589530	0.5711	2472	4.3933	YES	chr12	38448467	133779217	0.5185	8096	
CLL032TD	chr12	58339373	106852981	0.4025	2367	3.9625	YES	chr12	38453410	133779217	0.4287	8061	
CLL275TD	chr12	58339373	107002572	0.4796	2388	2.8367	YES	chr12	37957940	133779217	0.5145	8151	
CLL277TD	chr12	59268163	85421618	0.4686	1100	2.9491	YES	chr12	37957940	133779217	0.4656	8159	
CLL148TD	chr12	59272566	102590729	0.3855	2031	3.2140	YES	chr12	38503256	133779217	0.4875	8100	
CLL282TD	chr12	65632261	110023788	0.5019	2451	3.5810	YES	chr12	37957940	133779217	0.4997	8113	
CLL064TD	chr12	68947195	69250157	0.3382	52	2.8942	YES	chr12	38448467	133779217	0.4811	8132	
CLL064TD	chr12	69252656	70206718	0.5333	76	3.7798	YES	chr12	38448467	133779217	0.4811	8132	
CLL064TD	chr12	70209124	93100330	0.5969	796	4.2691	YES	chr12	38448467	133779217	0.4811	8132	
CLL277TD	chr12	85431870	103795358	0.4786	967	2.7710	YES	chr12	37957940	133779217	0.4656	8159	
CLL064TD	chr12	93101398	110353126	0.4844	1423	3.5024	YES	chr12	38448467	133779217	0.4811	8132	
CLL148TD	chr12	102591243	104190620	0.5601	105	3.5181	YES	chr12	38503256	133779217	0.4875	8100	
CLL277TD	chr12	103872105	133778670	0.4300	2953	2.3327	YES	chr12	37957940	133779217	0.4656	8159	
CLL148TD	chr12	104192319	107486510	0.4155	278	3.2793	YES	chr12	38503256	133779217	0.4875	8100	
CLL032TD	chr12	106857206	110353126	0.5424	369	3.3523	YES	chr12	38453410	133779217	0.4287	8061	
CLL275TD	chr12	107033079	122861921	0.5655	1720	3.3084	YES	chr12	37957940	133779217	0.5145	8151	
CLL148TD	chr12	107713729	110028530	0.5415	285	3.2609	YES	chr12	38503256	133779217	0.4875	8100	
CLL082TD	chr12	108600002	110296426	0.4317	259	2.4837	YES	chr12	38448467	133779217	0.5185	8096	
CLL282TD	chr12	110024521	132393420	0.4293	2038	2.8457	YES	chr12	37957940	133779217	0.4997	8113	
CLL148TD	chr12	110028983	110240721	0.8914	17	2.8876	YES	chr12	38503256	133779217	0.4875	8100	
CLL148TD	chr12	110246036	110399071	0.5541	30	3.0812	YES	chr12	38503256	133779217	0.4875	8100	
CLL082TD	chr12	110342509	113531304	0.5028	494	3.2910	YES	chr12	38448467	133779217	0.5185	8096	
CLL032TD	chr12	110354321	112585872	0.3912	338	3.0093	YES	chr12	38453410	133779217	0.4287	8061	
CLL064TD	chr12	110354321	113313454	0.3866	441	2.9193	YES	chr12	38448467	133779217	0.4811	8132	
CLL148TD	chr12	110399393	111070260	0.3941	126	2.8791	YES	chr12	38503256	133779217	0.4875	8100	
CLL045TD	chr12	110819504	133778670	0.4448	2168	2.5612	YES	chr12	38503256	133779217	0.4753	8112	
CLL148TD	chr12	111072078	111734249	0.5702	55	3.0231	YES	chr12	38503256	133779217	0.4875	8100	
CLL189TD	chr12	111731189	111749864	-0.6868	9	-2.0280	NO						
CLL148TD	chr12	111742005	111885425	1.0203	18	3.3952	YES	chr12	38503256	133779217	0.4875	8100	
CLL148TD	chr12	111885766	112223020	0.5195	60	3.2327	YES	chr12	38503256	133779217	0.4875	8100	
CLL148TD	chr12	112235843	112568289	0.3750	56	2.6303	YES	chr12	38503256	133779217	0.4875	8100	
CLL148TD	chr12	112572489	112621925	0.6489	23	3.7049	YES	chr12	38503256	133779217	0.4875	8100	
CLL032TD	chr12	112587494	112600776	0.5154	6	3.0994	YES	chr12	38453410	133779217	0.4287	8061	
CLL032TD	chr12	112601875	112608873	1.0082	6	2.0653	YES	chr12	38453410	133779217	0.4287	8061	
CLL032TD	chr12	112610449	112919823	0.4221	78	4.2081	YES	chr12	38453410	133779217	0.4287	8061	
CLL148TD	chr12	112630809	112681119	0.5656	28	4.4077	YES	chr12	38503256	133779217	0.4875	8100	
CLL148TD	chr12	112681374	113332363	0.4260	60	3.7194	YES	chr12	38503256	133779217	0.4875	8100	
CLL032TD	chr12	112924235	122692905	0.5547	860	2.8171	YES	chr12	38453410	133779217	0.4287	8061	
CLL064TD	chr12	113314438	113444191	0.6062	39	3.7692	YES	chr12	38448467	133779217	0.4811	8132	
CLL148TD	chr12	113333569	113836431	0.7490	138	3.1809	YES	chr12	38503256	133779217	0.4875	8100	
CLL064TD	chr12	113445418	113744249	0.3388	83	1.9352	YES	chr12	38448467	133779217	0.4811	8132	
CLL082TD	chr12	113640726	123342878	0.4468	934	2.5156	YES	chr12	38448467	133779217	0.5185	8096	
CLL064TD	chr12	113745422	120599280	0.4941	397	3.0316	YES	chr12	38448467	133779217	0.4811	8132	
CLL148TD	chr12	113836858	120518635	0.5528	329	3.3770	YES	chr12	38503256	133779217	0.4875	8100	
CLL148TD	chr12	120527694	122692905	0.6787	389	3.0497	YES	chr12	38503256	133779217	0.4875	8100	
CLL064TD	chr12	120599622	133778670	0.3937	1306	2.5881	YES	chr12	38448467	133779217	0.4811	8132	
CLL166TD	chr12	120875920	133778670	0.5172	1237	2.9137	YES	chr12	38572477	133779217	0.3933	8121	
CLL145TD	chr12	121861133	123048222	-0.8112	223	-3.0364	YES	chr12	121860338	123047826	-0.7281	222	
CLL148TD	chr12	122693224	123334540	0.4153	156	3.3940	YES	chr12	38503256	133779217	0.4875	8100	
CLL032TD	chr12	122693224	123343348	0.3493	169	3.3913	YES	chr12	38453410	133779217	0.4287	8061	
CLL275TD	chr12	122864866	123307879	0.3691	108	2.5509	YES	chr12	37957940	133779217	0.5145	8151	
CLL275TD	chr12	123310932	123519038	0.7735	66	3.3556	YES	chr12	37957940	133779217	0.5145	8151	
CLL148TD	chr12	123335274	123489730	0.8520	52	2.5528	YES	chr12	38503256	133779217	0.4875	8100	
CLL032TD	chr12	123343937	123498331	0.8713	44	1.9784	YES	chr12	38453410	133779217	0.4287	8061	
CLL082TD	chr12	123351608	133778670	0.4326	766	2.3501	YES	chr12	38448467	133779217	0.5185	8096	
CLL005TD	chr12	123467064	123519038	0.9198	21	3.0206	YES	chr12	38453410	133779217	0.5251	8176	
CLL148TD	chr12	123494360	124416451	0.5013	230	3.5119	YES	chr12	38503256	133779217	0.4875	8100	
CLL032TD	chr12	123519038	124411155	0.4264	219	3.5414	YES	chr12	38453410	133779217	0.4287	8061	
CLL275TD	chr12	123637065	124333232	0.4476	185	2.7007	YES	chr12	37957940	133779217	0.5145	8151	
CLL005TD	chr12	123637065	133778670	0.5207	744	3.1279	YES	chr12	38453410	133779217	0.5251	8176	
CLL275TD	chr12	124335408	132627197	0.6826	393	3.8918	YES	chr12	37957940	133779217	0.5145	8151	
CLL032TD	chr12	124412913	132853802	0.6546	363	2.7594	YES	chr12	38453410	133779217	0.4287	8061	
CLL148TD	chr12	124417847	125621158	0.8143	94	3.0156	YES	chr12	38503256	133779217	0.4875	8100	
CLL148TD	chr12	125626556	131484883	0.5369	112	3.4582	YES	chr12	38503256	133779217	0.4875	8100	
CLL148TD	chr12	131487280	133245211	0.8207	192	3.2388	YES	chr12	38503256	133779217	0.4875	8100	
CLL282TD	chr12	132394763	133778670	0.3822	244	2.3364	YES	chr12	37957940	133779217	0.4997	8113	
CLL275TD	chr12	132630096	133196573	1.0447	27	3.4510	YES	chr12	37957940	133779217	0.5145	8151	

CLL032TD	chr12	133196802	133423582	0.5995	98	2.3829	YES	chr12	38453410	133779217	0.4287	8061	
CLL275TD	chr12	133196802	133778670	0.5861	137	3.5875	YES	chr12	37957940	133779217	0.5145	8151	
CLL148TD	chr12	133245340	133454130	0.6819	72	3.1765	YES	chr12	38503256	133779217	0.4875	8100	
CLL032TD	chr12	133424615	133778670	0.3800	37	3.4107	YES	chr12	38453410	133779217	0.4287	8061	
CLL148TD	chr12	133501953	133778670	0.3676	28	3.2662	YES	chr12	38503256	133779217	0.4875	8100	
CLL141TD	chr13	20978234	21729228	-0.9476	72	-3.7309	YES	chr13	20906308	22405579	-0.9435	111	
CLL141TD	chr13	21746410	22275313	-0.9187	34	-3.4073	YES	chr13	20906308	22405579	-0.9435	111	
CLL146TD	chr13	34837007	70681587	-0.9006	1217	-3.7898	YES	chr13	34752795	70922816	-0.7916	1217	
CLL110TD	chr13	38923997	52293288	-0.7827	764	-3.3982	YES	chr13	38733308	53483162	-0.7936	919	
CLL194TD	chr13	41485871	44476730	-0.9826	204	-3.6015	YES	chr13	41434188	52354921	-0.9053	667	
CLL321TD	chr13	41635750	50510258	-0.8777	585	-3.9145	YES	chr13	41561808	52237129	-0.8764	652	
CLL194TD	chr13	44734557	52351166	-0.9436	461	-3.6848	YES	chr13	41434188	52354921	-0.9053	667	
CLL189TD	chr13	46357452	80910886	-0.5062	931	-2.0621	YES	chr13	46334152	82152329	-0.4679	931	
CLL192TD	chr13	46917419	51522103	-0.7799	254	-3.9047	YES	chr13	46913252	51522316	-0.7334	254	
CLL141TD	chr13	46917419	73428183	-0.9020	596	-3.6167	YES	chr13	46915859	73435221	-0.8720	596	
CLL188TD	chr13	47297320	50505091	-1.0137	196	-3.8394	YES	chr13	47295273	53161169	-0.9120	395	
CLL182TD	chr13	48517452	50502017	-0.4449	181	-2.1704	NO						SUBCLONE
CLL027TD	chr13	48517452	51527990	-0.8010	201	-3.5821	YES	chr13	48501167	51571802	-0.8240	202	
CLL030TD	chr13	48517452	53016485	-0.8124	355	-3.4252	YES	chr13	47563657	53040052	-0.8205	363	
CLL267TD	chr13	48517452	62334304	-0.8706	462	-3.6216	YES	chr13	47507142	62915894	-0.7481	462	
CLL083TD	chr13	48517452	64417580	-0.9101	470	-3.6942	YES	chr13	47946084	65226370	-0.8482	470	
CLL272TD	chr13	48570947	49822963	-0.9082	88	-3.8393	YES	chr13	48569200	51600231	-0.6386	204	
CLL172TD	chr13	48651240	50366552	-0.7516	163	-3.3188	YES	chr13	48629192	50438731	-0.7113	163	
CLL279TD	chr13	48708461	52863912	-0.8923	318	-3.8150	YES	chr13	48703533	52885510	-0.8388	318	
CLL181TD	chr13	48827887	50505091	-0.3781	159	-1.6611	YES	chr13	48740315	52314639	-0.4214	230	
CLL186TD	chr13	48827887	52701409	-0.5998	293	-2.8210	YES	chr13	48740315	52707274	-0.5630	293	
CLL184TD	chr13	48881358	57747585	-0.8533	391	-3.6616	YES	chr13	48858287	58200326	-0.7885	391	
CLL117TD	chr13	48916672	50495604	-0.4543	149	-2.1713	YES	chr13	48909694	51700648	-0.5751	185	
CLL053TD	chr13	48941624	51603719	-0.5964	179	-2.8316	YES	chr13	48919079	51700648	-0.6254	186	
CLL013TD	chr13	49760048	50123538	-0.6573	62	-2.9704	YES	chr13	49759884	51581416	-0.7134	121	
CLL157TD	chr13	49822963	51594513	-0.9695	114	-4.0961	YES	chr13	49813764	51593526	-0.8986	113	
CLL013TD	chr13	50125412	50366552	-0.9114	30	-3.3388	YES	chr13	49759884	51581416	-0.7134	121	
CLL144TD	chr13	50366552	51508957	-0.7432	22	-2.7499	YES	chr13	50389525	51568793	-0.7145	27	
CLL272TD	chr13	50366552	51517433	-0.9001	25	-3.8312	YES	chr13	48569200	51600231	-0.6386	204	
CLL013TD	chr13	50465395	51581081	-0.6070	29	-2.9127	YES	chr13	49759884	51581416	-0.7134	121	
CLL280TD	chr13	50489187	51397432	-1.0093	15	-3.9806	YES	chr13	50432170	51645041	-0.8038	39	
CLL323TD	chr13	50495604	51417260	-0.6220	16	-3.3454	YES	chr13	50569953	51483819	-0.6924	13	
CLL117TD	chr13	50502017	51608069	-0.7927	36	-4.1792	YES	chr13	48909694	51700648	-0.5751	185	
CLL182TD	chr13	50505091	51527990	-1.0074	24	-3.9775	NO						SUBCLONE
CLL172TD	chr13	50510258	50747127	-1.9621	10	-6.6197	YES	chr13	50506691	51479294	-1.7397	13	
CLL040TD	chr13	50586025	51287304	-4.5222	10	-8.5208	YES	chr13	50557545	51372504	-2.1297	10	
CLL159TD	chr13	50586025	51287304	-1.5641	10	-5.6372	YES	chr13	50557545	51375978	-1.2829	10	
CLL181TD	chr13	50586025	51416933	-0.9525	12	-3.8231	YES	chr13	48740315	52314639	-0.4214	230	
CLL188TD	chr13	50586025	51416933	-1.4858	12	-5.3196	YES	chr13	47295273	53161169	-0.9120	395	
CLL274TD	chr13	50586025	51416933	-2.2354	12	-6.4344	YES	chr13	50569953	51469532	-1.6291	12	
CLL174TD	chr13	50586025	51417260	-3.6344	13	-7.3596	YES	chr13	50520557	51527535	-2.6065	21	
CLL017TD	chr13	50586025	51504796	-0.8498	15	-3.8226	NO						SUBCLONE
CLL136TD	chr13	50586025	51504796	-0.8482	15	-3.0379	YES	chr13	50516370	51526121	-0.7490	20	
CLL052TD	chr13	50586025	51523559	-0.4579	20	-1.9180	YES	chr13	50559222	51551357	-0.4880	22	
CLL321TD	chr13	50586025	51530431	-4.9730	23	-7.7786	YES	chr13	41561808	52237129	-0.8764	652	
CLL041TD	chr13	50623062	51501518	-0.6784	11	-2.7956	YES	chr13	50608201	51501750	-0.6374	11	
CLL322TD	chr13	50623062	51530431	-3.5042	20	-6.8704	YES	chr13	50616393	51553735	-2.4118	20	
CLL172TD	chr13	51287304	51527990	-1.3256	12	-4.5739	YES	chr13	50506691	51479294	-1.7397	13	
CLL040TD	chr13	51397432	51530431	-0.9708	12	-3.9321	YES	chr13	51377832	51540139	-0.3716	12	
CLL280TD	chr13	51416933	51608069	-0.5143	23	-2.2064	YES	chr13	50432170	51645041	-0.8038	39	
CLL174TD	chr13	51501518	51527990	-1.4807	9	-5.5631	YES	chr13	50520557	51527535	-2.6065	21	
CLL181TD	chr13	51501518	52293288	-0.3178	57	-1.7038	YES	chr13	48740315	52314639	-0.4214	230	
CLL188TD	chr13	51501518	53096849	-0.9868	175	-3.9234	YES	chr13	47295273	53161169	-0.9120	395	
CLL172TD	chr13	51530431	51805409	-1.0700	14	-6.0542	NO						SUBCLONE
CLL182TD	chr13	51530431	76444992	-0.4358	437	-2.0838	NO						SUBCLONE
CLL272TD	chr13	51580936	51599401	-0.8796	8	-5.9200	YES	chr13	48569200	51600231	-0.6386	204	
CLL321TD	chr13	51580936	52234705	-0.9581	44	-4.0771	YES	chr13	41561808	52237129	-0.8764	652	
CLL110TD	chr13	52301809	52538968	-1.1243	33	-3.7414	YES	chr13	38733308	53483162	-0.7936	919	
CLL110TD	chr13	52542541	53419543	-0.7604	122	-3.0683	YES	chr13	38733308	53483162	-0.7936	919	
CLL030TD	chr13	53030639	53039808	-0.8560	6	-3.4674	YES	chr13	47563657	53040052	-0.8205	363	
CLL188TD	chr13	53103339	53153470	-3.9766	9	-1.7531	YES	chr13	47295273	53161169	-0.9120	395	
CLL110TD	chr13	101294454	101712124	-0.4274	9	-1.6181	NO						
CLL274TD	chr13	101308588	101717714	0.5984	10	1.6318	NO						
CLL023TD	chr14	19558957	19585260	0.6538	8	1.6166	NO						
CLL141TD	chr14	20697540	20764486	-0.7731	7	-2.4436	YES	chr14	20760146	20765549	-0.9383	4	
CLL155TD	chr14	23991120	25901187	-0.4691	417	-1.6420	NO						SUBCLONE
CLL155TD	chr14	38306651	39839695	-0.3130	86	-1.8063	NO						SUBCLONE
CLL141TD	chr14	58598184	58796653	-0.9766	37	-3.7360	YES	chr14	58585617	58808196	-0.9673	37	
CLL191TD	chr14	73719292	73733424	0.4290	16	1.6628	NO						
CLL274TD	chr14	77718066	77745021	0.9966	8	2.0994	NO						
CLL191TD	chr14	105617586	105676846	0.9094	9	1.9495	NO						IGH
CLL048TD	chr14	106130944	106174668	-0.3578	8	-1.8234	NO						IGH
CLL052TD	chr14	106130944	106330387	-1.7185	36	-3.0434	YES	chr14	106168179	107015590	-1.4376	124	

CLL159TD	chr14	106130944	106329377	-1.3216	37	-2.2826	YES	chr14	106168179	106410678	-0.7433	62	
CLL173TD	chr14	106133121	106237415	-1.3629	24	-2.6304	NO						IGH
CLL144TD	chr14	106174088	106209080	-1.2127	10	-2.5395	YES	chr14	106168179	106777490	-0.9957	96	
CLL032TD	chr14	106188116	106209080	-0.7952	7	-2.5989	NO						IGH
CLL045TD	chr14	106188116	106209080	-0.9708	8	-2.1875	NO						IGH
CLL276TD	chr14	106188116	106329377	-1.0992	28	-1.9216	YES	chr14	106252596	106303121	-0.8381	0	
CLL048TD	chr14	106188116	106366443	-3.4155	37	-3.7502	YES	chr14	106252596	106561323	-1.9852	51	
CLL192TD	chr14	106188116	106346841	-4.7516	37	-3.9438	YES	chr14	106252596	107208787	-0.6230	137	
CLL145TD	chr14	106204080	106209080	-2.6671	6	-4.4185	YES	chr14	106168179	107083152	-0.6834	141	
CLL186TD	chr14	106204080	106237415	-5.5469	15	-4.5364	YES	chr14	106168179	106811048	-1.0292	109	
CLL322TD	chr14	106204080	106329987	-1.0533	28	-1.6795	YES	chr14	106252596	106411088	-0.9570	45	
CLL009TD	chr14	106232232	106237415	-3.0135	9	-4.1107	NO						IGH
CLL090TD	chr14	106232232	106237415	-1.0598	9	-2.1099	NO						IGH
CLL043TD	chr14	106232232	106307218	-2.8044	11	-4.2632	YES	chr14	106252596	106829408	-1.7132	88	
CLL178TD	chr14	106232232	106321000	-1.2260	17	-2.4237	YES	chr14	106243009	106691076	-0.9101	68	
CLL017TD	chr14	106232232	106329377	-0.9118	19	-2.3284	YES	chr14	106243009	106827263	-1.0022	82	
CLL174TD	chr14	106232232	106329987	-0.8755	20	-2.0385	YES	chr14	106243009	106322375	-0.7534	9	
CLL189TD	chr14	106232232	106329987	-0.8668	20	-2.0127	NO						IGH
CLL280TD	chr14	106232232	106329987	-1.0074	20	-2.3658	YES	chr14	106252596	106322375	-0.7332	9	
CLL064TD	chr14	106232232	106329377	-0.9631	21	-2.2028	YES	chr14	106252596	106779188	-0.8215	83	
CLL110TD	chr14	106232232	106330387	-1.1768	21	-2.5712	YES	chr14	106243009	107094934	-0.7319	126	
CLL197TD	chr14	106232232	106330387	-1.0805	21	-1.9559	YES	chr14	106252596	106829408	-0.7154	87	
CLL040TD	chr14	106232232	106330760	-1.2225	22	-2.2656	YES	chr14	106243009	106827263	-0.3900	88	
CLL145TD	chr14	106232232	106330387	-0.6515	22	-1.9498	YES	chr14	106168179	107083152	-0.6834	141	
CLL045TD	chr14	106232232	106330760	-3.0340	24	-3.6521	YES	chr14	106243009	107179117	-0.7934	132	
CLL144TD	chr14	106232232	106369421	-6.6731	36	-3.8056	YES	chr14	106168179	106777490	-0.9957	96	
CLL191TD	chr14	106235554	106329987	-0.6049	18	-1.7845	YES	chr14	106252596	107012899	-0.8324	107	
CLL136TD	chr14	106237415	106329987	-0.6444	11	-2.3135	YES	chr14	106252596	106303121	-0.7909	0	
CLL184TD	chr14	106237415	106330387	-1.0780	15	-1.6901	YES	chr14	106257663	107048607	-0.8605	120	
CLL172TD	chr14	106304633	106321986	-1.0642	10	-2.3240	YES	chr14	106252596	106322375	-0.7866	10	
CLL008TD	chr14	106304633	106329987	-0.7630	12	-2.1526	YES	chr14	106243009	107167303	-0.8026	131	
CLL009TD	chr14	106304633	106330387	-0.8045	12	-1.7481	YES	chr14	106243009	106734526	-0.7843	79	
CLL278TD	chr14	106304633	106329987	-0.8399	12	-1.6766	YES	chr14	106252596	106691076	-0.6082	70	
CLL282TD	chr14	106304633	106329987	-0.7720	13	-1.9926	YES	chr14	106263491	106322375	-0.8652	11	
CLL290TD	chr14	106304633	106329987	-1.0331	13	-2.3069	YES	chr14	106243009	106561323	-0.8239	55	
CLL186TD	chr14	106304633	106370309	-9.9055	33	-3.6032	YES	chr14	106168179	106811048	-1.0292	109	
CLL173TD	chr14	106304633	106405971	-9.9367	45	-3.6496	YES	chr14	106252596	106831538	-1.9681	92	
CLL043TD	chr14	106308206	106405971	-3.4445	41	-3.2766	YES	chr14	106252596	106829408	-1.7132	88	
CLL100TD	chr14	106329377	106346841	-9.0224	8	-2.8042	YES	chr14	106330168	106345551	-3.3787	5	
CLL279TD	chr14	106329377	106359349	-3.3636	15	-2.5594	YES	chr14	106330168	106345551	-3.1367	4	
CLL172TD	chr14	106329377	106379028	-9.7387	26	-2.9754	YES	chr14	106330168	106538621	-2.1448	40	
CLL274TD	chr14	106329377	106405971	-9.7964	28	-3.4616	YES	chr14	106330168	106610162	-1.1555	46	
CLL175TD	chr14	106329377	106478456	-9.7055	38	-3.5220	YES	chr14	106330168	107083152	-2.5047	110	
CLL124TD	chr14	106329377	106714578	-0.7779	63	-1.8724	YES	chr14	106330168	106724962	-0.5153	61	
CLL064TD	chr14	106329987	106356996	-7.6821	12	-2.8722	YES	chr14	106252596	106779188	-0.8215	83	
CLL159TD	chr14	106329987	106360313	-8.1293	12	-3.0692	YES	chr14	106168179	106410678	-0.7433	62	
CLL041TD	chr14	106329987	106370493	-1.7754	20	-2.1101	YES	chr14	106330168	106524457	-0.7176	39	
CLL141TD	chr14	106329987	106375713	-9.9491	20	-3.1210	YES	chr14	106330168	106345551	-4.2148	3	
CLL023TD	chr14	106329987	106379028	-7.5893	25	-2.8851	YES	chr14	106320095	107169498	-0.7311	122	
CLL277TD	chr14	106329987	106378065	-9.7456	25	-2.9875	YES	chr14	106330168	106345551	-3.9939	4	
CLL136TD	chr14	106330387	106357506	-4.4873	8	-2.6532	YES	chr14	106331756	106826216	-1.8593	66	
CLL027TD	chr14	106330387	106356996	-8.9239	11	-2.8816	YES	chr14	106320095	107182803	-0.8139	131	
CLL083TD	chr14	106330387	106357506	-8.6562	12	-2.8731	YES	chr14	106330168	106345551	-3.4146	5	
CLL171TD	chr14	106330387	106363769	-3.8134	12	-2.9225	YES	chr14	106330168	107083152	-0.7604	109	
CLL178TD	chr14	106330387	106363769	-9.5637	12	-3.1116	YES	chr14	106243009	106691076	-0.9101	68	
CLL267TD	chr14	106330387	106357506	-4.1548	12	-2.6611	YES	chr14	106331756	106345551	-2.5360	1	
CLL278TD	chr14	106330387	106366443	-5.0056	14	-2.8969	YES	chr14	106252596	106691076	-0.6082	70	
CLL189TD	chr14	106330387	106369421	-8.2337	16	-2.9375	YES	chr14	106330168	106918315	-1.3986	83	
CLL273TD	chr14	106330387	106373023	-4.3847	20	-2.5511	YES	chr14	106330168	106877502	-0.7216	84	
CLL290TD	chr14	106330387	106376217	-9.1349	20	-3.0638	YES	chr14	106243009	106561323	-0.8239	55	
CLL032TD	chr14	106330387	106379028	-6.9021	21	-2.9824	YES	chr14	106330168	106345551	-3.2441	3	
CLL266TD	chr14	106330387	106370493	-7.8500	21	-2.9798	YES	chr14	106330168	106345551	-3.2449	5	
CLL181TD	chr14	106330387	106380172	-10.0000	24	-3.0424	YES	chr14	106330168	106610162	-0.9352	50	
CLL280TD	chr14	106330387	106388010	-9.9417	26	-3.1735	YES	chr14	106330168	107012899	-2.3117	97	
CLL017TD	chr14	106330387	106405971	-8.3142	27	-3.4088	YES	chr14	106243009	106827263	-1.0022	82	
CLL322TD	chr14	106330387	106405586	-9.6251	31	-3.1455	YES	chr14	106252596	106411088	-0.9570	45	
CLL007TD	chr14	106330387	106494494	-3.3737	37	-3.1163	YES	chr14	106330168	106517682	-2.0845	37	
CLL019TD	chr14	106330387	106579220	-9.5583	46	-3.9811	YES	chr14	106330168	106724962	-1.7993	61	
CLL082TD	chr14	106330387	106816090	-8.1022	71	-4.2468	YES	chr14	106331756	106345551	-3.3155	0	
CLL054TD	chr14	106330760	106351843	-7.6209	7	-3.0294	YES	chr14	106331756	106345551	-3.7211	0	
CLL275TD	chr14	106330760	106351843	-10.0000	8	-2.9762	YES	chr14	106330168	106610162	-0.8714	51	
CLL009TD	chr14	106330760	106357506	-3.3954	11	-2.6653	YES	chr14	106243009	106734526	-0.7843	79	
CLL063TD	chr14	106330760	106360313	-6.0694	12	-2.8341	YES	chr14	106331756	106345551	-3.8480	0	
CLL155TD	chr14	106330760	106360313	-10.0000	13	-3.0219	YES	chr14	106331756	106345551	-3.7543	1	
CLL197TD	chr14	106330760	106360313	-8.3290	13	-2.9741	YES	chr14	106252596	106829408	-0.7154	87	
CLL157TD	chr14	106330760	106363769	-9.9654	14	-3.0537	YES	chr14	106326556	106526710	-0.9814	43	
CLL038TD	chr14	106330760	106369421	-2.6953	15	-2.5613	YES	chr14	106330168	106561323	-1.6025	40	
CLL044TD	chr14	106330760	106369421	-7.1372	17	-2.9033	YES	chr14	106331756	106345551	-4.4615	1	

CLL148TD	chr14	106330760	106369421	-6.0154	17	-2.9558	YES	chr14	106330168	106526710	-0.9501	39	
CLL152TD	chr14	106330760	106369421	-5.9502	18	-2.8870	YES	chr14	106330168	107287646	-0.6983	134	
CLL170TD	chr14	106330760	106370309	-9.9163	18	-3.1611	YES	chr14	106330168	107210901	-0.7360	125	
CLL145TD	chr14	106330760	106379028	-5.2664	20	-2.9188	YES	chr14	106168179	107083152	-0.6834	141	
CLL053TD	chr14	106330760	106379028	-7.8069	23	-2.9223	YES	chr14	106330168	106345551	-3.5836	3	
CLL166TD	chr14	106330760	106380172	-9.9538	24	-3.0116	YES	chr14	106331756	106345551	-2.2126	0	
CLL117TD	chr14	106330760	106386891	-8.6995	26	-2.9833	YES	chr14	106331756	106345551	-3.5884	0	
CLL042TD	chr14	106330760	106478072	-1.5875	27	-3.1102	YES	chr14	106330168	106852841	-0.5597	74	
CLL013TD	chr14	106330760	106453023	-9.2941	31	-3.5583	YES	chr14	106330168	106877502	-0.8658	85	
CLL184TD	chr14	106330760	106405971	-6.9407	31	-3.2062	YES	chr14	106257663	107048607	-0.8605	120	
CLL323TD	chr14	106330760	106471216	-8.5546	34	-3.5535	YES	chr14	106330168	106691076	-0.8079	62	
CLL052TD	chr14	106330760	106586504	-3.5947	42	-3.8748	YES	chr14	106168179	107015590	-1.4376	124	
CLL049TD	chr14	106330760	106552646	-4.2708	43	-3.4485	YES	chr14	106330168	106993731	-0.8750	98	
CLL020TD	chr14	106331373	106351843	-9.4122	7	-2.8086	YES	chr14	106330168	106786127	-0.7892	67	
CLL319TD	chr14	106331373	106361449	-10.0000	12	-3.0189	YES	chr14	106331756	106345551	-2.6057	1	
CLL270TD	chr14	106331373	106370493	-9.9659	16	-3.0874	YES	chr14	106331756	106345551	-4.5472	0	
CLL005TD	chr14	106331373	106370309	-9.2759	18	-3.0124	YES	chr14	106330168	107182803	-0.7506	120	
CLL030TD	chr14	106331373	106376217	-3.5610	18	-2.7810	YES	chr14	106331756	106345551	-3.1174	0	
CLL182TD	chr14	106331373	106380172	-9.8129	23	-2.9438	YES	chr14	106330168	107218278	-0.8158	125	
CLL272TD	chr14	106331581	106380172	-9.9266	21	-2.9896	YES	chr14	106330168	107081716	-0.9841	111	
CLL029TD	chr14	106331581	106386891	-9.9249	27	-2.9546	YES	chr14	106330168	107048607	-1.0459	109	
CLL006TD	chr14	106346841	106356996	-4.5195	6	-2.8648	YES	chr14	106330168	106526710	-1.0101	40	
CLL146TD	chr14	106346841	106357506	-9.1811	7	-3.0148	YES	chr14	106354195	106517682	-0.5970	28	
CLL090TD	chr14	106346841	106370309	-3.8011	14	-2.9557	YES	chr14	106330168	106724962	-1.5966	62	
CLL110TD	chr14	106346841	106379028	-3.7387	21	-2.7510	YES	chr14	106243009	107094934	-0.7319	126	
CLL174TD	chr14	106346841	106386891	-9.9147	25	-3.0002	YES	chr14	106330168	106724962	-2.2069	66	
CLL045TD	chr14	106347345	106357506	-3.1267	6	-2.6902	YES	chr14	106243009	107179117	-0.7934	132	
CLL100TD	chr14	106347345	106357506	-3.3141	6	-2.6621	NO						IGH
CLL040TD	chr14	106347345	106361449	-8.7600	10	-3.1893	YES	chr14	106243009	106827263	-0.3900	88	
CLL022TD	chr14	106347345	106375713	-4.1559	17	-2.9214	NO						IGH
CLL192TD	chr14	106347345	107283143	-0.9134	123	-2.2682	YES	chr14	106252596	107208787	-0.6230	137	
CLL194TD	chr14	106349710	106366443	-7.1575	8	-3.2879	YES	chr14	106354195	106453838	-0.6072	18	
CLL282TD	chr14	106349710	106405971	-9.7542	21	-3.6324	YES	chr14	106330168	106411088	-2.6317	27	
CLL008TD	chr14	106349710	106518770	-0.9451	35	-1.7780	YES	chr14	106243009	107167303	-0.8026	131	
CLL188TD	chr14	106349710	106714578	-0.9977	54	-2.2479	YES	chr14	106330168	106723316	-0.7276	59	
CLL054TD	chr14	106354362	106405971	-1.2861	23	-1.9737	YES	chr14	106354195	106411088	-0.5840	23	
CLL321TD	chr14	106354362	106518770	-1.4273	32	-1.9369	YES	chr14	106330168	107169498	-0.7248	117	
CLL020TD	chr14	106354362	106780862	-1.0163	58	-2.0027	YES	chr14	106330168	106786127	-0.7892	67	
CLL006TD	chr14	106357506	106368457	-9.8312	6	-3.1710	YES	chr14	106330168	106526710	-1.0101	40	
CLL027TD	chr14	106357506	106518770	-1.3217	30	-2.1360	YES	chr14	106320095	107182803	-0.8139	131	
CLL064TD	chr14	106357506	106518770	-0.7827	30	-2.0012	YES	chr14	106252596	106779188	-0.8215	83	
CLL045TD	chr14	106360313	106369421	-4.3540	6	-2.7719	YES	chr14	106243009	107179117	-0.7934	132	
CLL136TD	chr14	106360313	106388010	-9.8824	13	-3.3466	YES	chr14	106331756	106826216	-1.8593	66	
CLL146TD	chr14	106360313	106518370	-0.8627	26	-1.9291	YES	chr14	106354195	106517682	-0.5970	28	
CLL083TD	chr14	106360313	106518770	-1.0274	29	-2.2072	NO						IGH
CLL009TD	chr14	106360313	106714578	-0.7942	52	-1.7129	YES	chr14	106243009	106734526	-0.7843	79	
CLL267TD	chr14	106360313	107035079	-0.7080	88	-2.3445	NO						IGH
CLL155TD	chr14	106363769	106373023	-2.2913	8	-2.0558	YES	chr14	106354195	106411088	-0.5768	21	
CLL159TD	chr14	106363769	106471216	-1.3557	21	-1.9185	YES	chr14	106168179	106410678	-0.7433	62	
CLL040TD	chr14	106363769	106518770	-0.9156	28	-1.6188	YES	chr14	106243009	106827263	-0.3900	88	
CLL197TD	chr14	106363769	106829941	-0.9973	64	-1.8048	YES	chr14	106252596	106829408	-0.7154	87	
CLL171TD	chr14	106366443	107078725	-1.0207	97	-2.6198	YES	chr14	106330168	107083152	-0.7604	109	
CLL194TD	chr14	106366949	106453023	-0.8559	13	-2.6472	YES	chr14	106354195	106453838	-0.6072	18	
CLL278TD	chr14	106366949	106518770	-1.0271	24	-1.7990	YES	chr14	106252596	106691076	-0.6082	70	
CLL100TD	chr14	106368457	106780862	-4.2407	57	-4.3246	YES	chr14	106483522	106786127	-2.4305	35	
CLL045TD	chr14	106370309	106385308	-2.7030	8	-2.5518	YES	chr14	106243009	107179117	-0.7934	132	
CLL152TD	chr14	106370309	106518770	-0.9440	23	-2.0359	YES	chr14	106330168	107287646	-0.6983	134	
CLL005TD	chr14	106370493	106379028	-3.6154	6	-2.3208	YES	chr14	106330168	107182803	-0.7506	120	
CLL189TD	chr14	106370493	106382639	-3.3170	6	-2.5182	YES	chr14	106330168	106918315	-1.3986	83	
CLL186TD	chr14	106373023	106380172	-6.5615	6	-2.7390	YES	chr14	106168179	106811048	-1.0292	109	
CLL178TD	chr14	106373023	106453023	-0.9767	11	-2.3714	YES	chr14	106243009	106691076	-0.9101	68	
CLL144TD	chr14	106373023	106518770	-0.8355	20	-2.3186	YES	chr14	106168179	106777490	-0.9957	96	
CLL044TD	chr14	106373023	106518770	-0.8064	21	-1.9947	NO						IGH
CLL148TD	chr14	106373023	106518770	-0.9540	21	-2.3956	YES	chr14	106330168	106526710	-0.9501	39	
CLL266TD	chr14	106373023	106518770	-1.1219	22	-1.9808	NO						IGH
CLL048TD	chr14	106373023	106579220	-3.9707	28	-4.1558	YES	chr14	106252596	106561323	-1.9852	51	
CLL041TD	chr14	106373023	106586504	-0.5401	30	-1.7873	YES	chr14	106330168	106524457	-0.7176	39	
CLL006TD	chr14	106373023	106667950	-0.7320	41	-1.9870	YES	chr14	106330168	106526710	-1.0101	40	
CLL270TD	chr14	106373023	106974717	-1.1603	74	-2.5990	NO						IGH
CLL170TD	chr14	106373023	107283143	-0.9525	112	-2.8358	YES	chr14	106330168	107210901	-0.7360	125	
CLL155TD	chr14	106375713	106405971	-0.8926	9	-2.1024	YES	chr14	106354195	106411088	-0.5768	21	
CLL273TD	chr14	106375713	106518770	-0.7708	20	-1.8361	YES	chr14	106330168	106877502	-0.7216	84	
CLL038TD	chr14	106376217	106579220	-2.3488	26	-3.6327	YES	chr14	106330168	106561323	-1.6025	40	
CLL022TD	chr14	106376217	106780862	-0.8165	46	-2.3377	NO						IGH
CLL141TD	chr14	106376217	107042562	-7.7008	84	-5.1246	YES	chr14	106354195	106398480	-1.1591	19	
CLL290TD	chr14	106378065	106552646	-1.1611	22	-2.3808	YES	chr14	106243009	106561323	-0.8239	55	
CLL030TD	chr14	106378065	106790974	-1.1029	46	-2.3458	NO						IGH
CLL277TD	chr14	106379028	106453023	-1.6442	9	-3.0226	YES	chr14	106354195	106410678	-0.9011	23	

CLL090TD	chr14	106379028	106714578	-3.9606	40	-4.5511	YES	chr14	106330168	106724962	-1.5966	62	
CLL145TD	chr14	106380172	107078725	-0.7959	88	-2.4810	YES	chr14	106168179	107083152	-0.6834	141	
CLL005TD	chr14	106382639	106471216	-0.9334	9	-2.4464	YES	chr14	106330168	107182803	-0.7506	120	
CLL166TD	chr14	106382639	106471598	-1.1413	10	-2.8512	NO						IGH
CLL032TD	chr14	106382639	106518370	-0.8272	15	-2.3765	YES	chr14	106354195	106516175	-0.6502	28	
CLL051TD	chr14	106382639	106518370	-0.6511	15	-2.2766	YES	chr14	106330168	106517682	-0.6045	35	
CLL053TD	chr14	106382639	106518770	-1.1087	16	-2.2663	NO						IGH
CLL157TD	chr14	106382639	106518770	-1.0294	16	-2.2734	YES	chr14	106326556	106526710	-0.9814	43	
CLL172TD	chr14	106382639	106518770	-6.5509	16	-4.9992	YES	chr14	106330168	106538621	-2.1448	40	
CLL181TD	chr14	106382639	106518770	-1.2394	16	-2.6055	YES	chr14	106330168	106610162	-0.9352	50	
CLL186TD	chr14	106382639	106518770	-0.9747	16	-2.0680	YES	chr14	106168179	106811048	-1.0292	109	
CLL191TD	chr14	106382639	106518770	-0.7661	16	-1.9551	YES	chr14	106252596	107012899	-0.8324	107	
CLL272TD	chr14	106382639	106518770	-1.4227	16	-2.6390	YES	chr14	106330168	107081716	-0.9841	111	
CLL110TD	chr14	106382639	106780862	-0.9399	47	-2.4535	YES	chr14	106243009	107094934	-0.7319	126	
CLL182TD	chr14	106382639	107283143	-0.9601	104	-2.2631	YES	chr14	106330168	107218278	-0.8158	125	
CLL189TD	chr14	106385308	106518770	-1.9812	15	-4.0744	YES	chr14	106330168	106918315	-1.3986	83	
CLL063TD	chr14	106385308	106539432	-0.9603	17	-2.5101	NO						IGH
CLL045TD	chr14	106386891	106518770	-0.8168	14	-2.2994	YES	chr14	106243009	107179117	-0.7934	132	
CLL029TD	chr14	106388010	106539432	-7.4365	15	-5.4218	YES	chr14	106330168	107048607	-1.0459	109	
CLL117TD	chr14	106388010	106539432	-4.9691	15	-5.3148	YES	chr14	106354195	106398480	-1.2201	21	
CLL174TD	chr14	106388010	106714578	-5.9935	36	-4.9610	YES	chr14	106330168	106724962	-2.2069	66	
CLL136TD	chr14	106405586	106815691	-5.3440	47	-4.5943	YES	chr14	106331756	106826216	-1.8593	66	
CLL280TD	chr14	106405586	106994184	-8.2232	71	-5.2026	YES	chr14	106330168	107012899	-2.3117	97	
CLL017TD	chr14	106452641	106518770	-0.6930	10	-2.4315	YES	chr14	106243009	106827263	-1.0022	82	
CLL184TD	chr14	106452641	106573203	-0.7088	15	-1.6962	YES	chr14	106257663	107048607	-0.8605	120	
CLL043TD	chr14	106452641	106815691	-2.7739	45	-4.0323	YES	chr14	106252596	106829408	-1.7132	88	
CLL173TD	chr14	106452641	106815691	-7.8921	45	-4.5566	YES	chr14	106252596	106831538	-1.9681	92	
CLL013TD	chr14	106471216	106518770	-1.2356	8	-2.3213	YES	chr14	106330168	106877502	-0.8658	85	
CLL274TD	chr14	106471598	106518770	-1.1222	7	-2.5065	YES	chr14	106330168	106610162	-1.1555	46	
CLL323TD	chr14	106471598	106667950	-1.0314	27	-2.2843	YES	chr14	106330168	106691076	-0.8079	62	
CLL042TD	chr14	106478456	106780862	-0.5910	36	-2.0657	YES	chr14	106330168	106852841	-0.5597	74	
CLL178TD	chr14	106494114	106667950	-1.0761	24	-2.4408	YES	chr14	106243009	106691076	-0.9101	68	
CLL005TD	chr14	106494114	107179179	-0.8698	82	-2.6884	YES	chr14	106330168	107182803	-0.7506	120	
CLL007TD	chr14	106518370	106714578	-0.5846	25	-1.6552	YES	chr14	106519396	106724962	-0.6403	23	
CLL175TD	chr14	106539432	106579220	-9.9261	7	-3.2213	YES	chr14	106330168	107083152	-2.5047	110	
CLL117TD	chr14	106552257	106641533	-0.7576	13	-2.4142	NO						IGH
CLL029TD	chr14	106552257	106791373	-1.0915	31	-2.0325	YES	chr14	106330168	107048607	-1.0459	109	
CLL275TD	chr14	106573203	106622262	-1.0206	10	-2.7397	YES	chr14	106330168	106610162	-0.8714	51	
CLL274TD	chr14	106573203	106641533	-0.8573	11	-2.6522	YES	chr14	106330168	106610162	-1.1555	46	
CLL017TD	chr14	106573203	106780482	-0.8731	26	-2.2794	YES	chr14	106243009	106827263	-1.0022	82	
CLL049TD	chr14	106573203	106780482	-0.7634	26	-1.9603	YES	chr14	106330168	106993731	-0.8750	98	
CLL064TD	chr14	106573203	106780482	-0.8926	26	-2.3684	YES	chr14	106252596	106779188	-0.8215	83	
CLL189TD	chr14	106573203	106780862	-2.1427	27	-4.1281	YES	chr14	106330168	106918315	-1.3986	83	
CLL191TD	chr14	106573203	106780862	-0.9367	27	-2.5173	YES	chr14	106252596	107012899	-0.8324	107	
CLL321TD	chr14	106573203	106790974	-1.1435	28	-2.4434	YES	chr14	106330168	107169498	-0.7248	117	
CLL083TD	chr14	106573203	106791373	-0.7816	29	-2.5324	NO						IGH
CLL144TD	chr14	106573203	106791373	-0.8039	29	-2.3375	YES	chr14	106168179	106777490	-0.9957	96	
CLL186TD	chr14	106573203	106815691	-0.9825	31	-2.2968	YES	chr14	106168179	106811048	-1.0292	109	
CLL040TD	chr14	106573203	106829563	-0.9668	32	-2.1573	YES	chr14	106243009	106827263	-0.3900	88	
CLL266TD	chr14	106573203	106829563	-0.9135	32	-2.2213	NO						IGH
CLL013TD	chr14	106573203	106866773	-1.0982	41	-2.7118	YES	chr14	106330168	106877502	-0.8658	85	
CLL053TD	chr14	106573203	107042562	-1.0808	61	-2.8464	NO						IGH
CLL045TD	chr14	106573203	107083602	-0.8701	69	-2.6099	YES	chr14	106243009	107179117	-0.7934	132	
CLL027TD	chr14	106573203	107083602	-0.9766	70	-2.4843	YES	chr14	106320095	107182803	-0.8139	131	
CLL008TD	chr14	106573203	107131398	-0.9589	75	-2.6535	YES	chr14	106243009	107167303	-0.8026	131	
CLL044TD	chr14	106573203	107178794	-0.8343	79	-2.1425	YES	chr14	107151892	107169498	-1.4230	0	
CLL152TD	chr14	106573203	107283143	-0.7168	88	-2.3787	YES	chr14	106330168	107287646	-0.6983	134	
CLL278TD	chr14	106573597	106667950	-0.9226	15	-2.4059	YES	chr14	106252596	106691076	-0.6082	70	
CLL184TD	chr14	106573597	106829563	-1.4963	32	-2.1603	YES	chr14	106257663	107048607	-0.8605	120	
CLL273TD	chr14	106573597	106877972	-1.0273	41	-2.4129	YES	chr14	106330168	106877502	-0.7216	84	
CLL048TD	chr14	106586106	106622262	-0.7856	6	-2.8996	NO						IGH
CLL019TD	chr14	106586106	106653461	-7.0818	9	-5.5147	YES	chr14	106330168	106724962	-1.7993	61	
CLL038TD	chr14	106586106	106791373	-0.6606	25	-1.9891	YES	chr14	106632143	106779188	-0.5882	15	
CLL175TD	chr14	106586106	107078725	-7.2572	62	-5.6676	YES	chr14	106330168	107083152	-2.5047	110	
CLL052TD	chr14	106610286	106856254	-2.3445	30	-3.8999	YES	chr14	106168179	107015590	-1.4376	124	
CLL019TD	chr14	106663366	106714578	-9.1864	6	-5.3595	YES	chr14	106330168	106724962	-1.7993	61	
CLL090TD	chr14	106725171	106805561	-0.9174	12	-1.6604	YES	chr14	106726871	106829408	-0.5597	11	
CLL174TD	chr14	106733114	107034699	-0.9403	38	-2.8728	YES	chr14	106726871	107012899	-0.7352	35	
CLL019TD	chr14	106733114	107083602	-0.7270	47	-2.6257	NO						IGH
CLL272TD	chr14	106780862	107083225	-1.0860	42	-3.0058	YES	chr14	106330168	107081716	-0.9841	111	
CLL100TD	chr14	106790974	107169901	-0.9732	48	-3.0571	YES	chr14	106825816	107150851	-0.7333	43	
CLL049TD	chr14	106805181	106974717	-0.7949	25	-2.4744	YES	chr14	106330168	106993731	-0.8750	98	
CLL042TD	chr14	106815691	106858875	-0.5635	8	-2.9601	YES	chr14	106330168	106852841	-0.5597	74	
CLL082TD	chr14	106829563	106866773	-1.1196	9	-3.5838	YES	chr14	106833210	106877502	-0.7007	7	
CLL189TD	chr14	106829563	106877972	-1.3856	11	-3.7524	YES	chr14	106330168	106918315	-1.3986	83	
CLL191TD	chr14	106829563	107012911	-0.8653	25	-3.0239	YES	chr14	106252596	107012899	-0.8324	107	
CLL136TD	chr14	106829563	107034699	-0.9639	27	-3.6669	YES	chr14	106829008	107041398	-0.7250	28	
CLL173TD	chr14	106829563	107042562	-1.0421	29	-3.4730	YES	chr14	106252596	106831538	-1.9681	92	

CLL029TD	chr14	106829563	107048641	-0.9554	30	-2.8497	YES	chr14	106330168	107048607	-1.0459	109	
CLL083TD	chr14	106829563	107083602	-0.7144	37	-3.0324	NO						IGH
CLL110TD	chr14	106829563	107095088	-0.8444	38	-3.3085	YES	chr14	106243009	107094934	-0.7319	126	
CLL321TD	chr14	106829563	107131398	-1.0596	43	-3.0164	YES	chr14	106330168	107169498	-0.7248	117	
CLL023TD	chr14	106829563	107169901	-0.8562	44	-2.6618	YES	chr14	106320095	107169498	-0.7311	122	
CLL184TD	chr14	106829941	107042562	-0.8345	28	-2.6315	YES	chr14	106257663	107048607	-0.8605	120	
CLL052TD	chr14	106858875	107013314	-1.8241	21	-4.4795	YES	chr14	106168179	107015590	-1.4376	124	
CLL189TD	chr14	106926160	107013314	-0.4216	15	-2.6262	YES	chr14	106927167	107169498	-0.3894	30	
CLL280TD	chr14	107012911	107283143	-0.3559	31	-1.6800	YES	chr14	107013844	107081716	-0.8741	9	
CLL189TD	chr14	107034699	107131398	-0.7188	17	-2.8055	YES	chr14	106927167	107169498	-0.3894	30	
CLL172TD	chr14	107048641	107083225	-0.8724	7	-3.0084	YES	chr14	107050074	107083152	-0.7786	4	
CLL141TD	chr14	107048641	107283143	-1.1519	27	-2.0172	YES	chr14	107046530	107128311	-1.0109	12	
CLL045TD	chr14	107095088	107169901	-0.4504	7	-2.0548	YES	chr14	106243009	107179117	-0.7934	132	
CLL027TD	chr14	107095088	107179179	-1.3809	10	-2.2975	YES	chr14	106320095	107182803	-0.8139	131	
CLL083TD	chr14	107113711	107283143	-0.8322	17	-2.9388	YES	chr14	107159767	107181281	-1.4358	4	
CLL141TD	chr15	34295201	35376969	-0.9415	120	-3.8542	YES	chr15	34202339	37462956	-0.5534	158	
CLL141TD	chr15	36872051	37402382	-1.0103	27	-3.5577	YES	chr15	34202339	37462956	-0.5534	158	
CLL155TD	chr15	40056008	42492042	-0.5280	637	-1.9846	NO						SUBCLONE
CLL141TD	chr15	40253922	41571500	-1.0610	310	-3.5989	YES	chr15	40253746	41576166	-1.0220	310	
CLL189TD	chr15	42139477	42167617	-0.4072	30	-1.8939	NO						
CLL110TD	chr15	43022309	43028357	-0.5438	14	-1.7192	NO						
CLL100TD	chr15	50731262	51204250	-0.8320	84	-3.5269	YES	chr15	50651053	51058342	-0.9131	83	
CLL141TD	chr15	56385501	56436579	-1.0077	8	-7.3797	YES	chr15	56332487	56599346	-0.9313	9	
CLL175TD	chr15	65410996	65471184	-0.9515	19	-4.4094	YES	chr15	65368825	65478380	-0.8670	20	
CLL110TD	chr16	10971123	10995817	-0.7927	7	-1.9072	NO						
CLL145TD	chr16	50059526	51098366	-0.7230	108	-3.0494	YES	chr16	49962692	50875648	-0.7276	107	
CLL041TD	chr16	50328546	50339633	-0.9191	6	-1.6396	NO						
CLL275TD	chr16	67235259	67241734	0.6217	9	1.8107	NO						
CLL192TD	chr17	5967	2584999	-1.2569	376	-3.2789	YES	chr17	72804	18537304	-0.6050	3102	
CLL192TD	chr17	2596053	2599656	-1.8087	8	-1.9948	YES	chr17	72804	18537304	-0.6050	3102	
CLL189TD	chr17	2596053	2603925	-0.7670	11	-1.9002	NO						
CLL192TD	chr17	2599944	2934193	-1.3907	31	-2.8389	YES	chr17	72804	18537304	-0.6050	3102	
CLL192TD	chr17	2935648	3417866	-0.8273	32	-4.7041	YES	chr17	72804	18537304	-0.6050	3102	
CLL192TD	chr17	3419716	3807209	-1.3656	136	-3.0931	YES	chr17	72804	18537304	-0.6050	3102	
CLL192TD	chr17	3807568	3828027	-2.5119	6	-1.7495	YES	chr17	72804	18537304	-0.6050	3102	
CLL192TD	chr17	3828643	3917372	-1.6252	22	-2.2447	YES	chr17	72804	18537304	-0.6050	3102	
CLL192TD	chr17	3917604	4342925	-0.9298	80	-3.9638	YES	chr17	72804	18537304	-0.6050	3102	
CLL192TD	chr17	4348244	4435801	-1.3769	14	-2.0278	YES	chr17	72804	18537304	-0.6050	3102	
CLL192TD	chr17	4436197	4451242	-1.9605	19	-2.0787	YES	chr17	72804	18537304	-0.6050	3102	
CLL192TD	chr17	4451371	4457441	-1.2206	10	-2.3049	YES	chr17	72804	18537304	-0.6050	3102	
CLL192TD	chr17	4458143	4498380	-1.9351	10	-1.8688	YES	chr17	72804	18537304	-0.6050	3102	
CLL192TD	chr17	4500422	4594644	-1.2243	29	-3.3052	YES	chr17	72804	18537304	-0.6050	3102	
CLL145TD	chr17	4585772	4910709	-0.7493	187	-2.6673	YES	chr17	4579986	4912490	-0.6841	188	
CLL192TD	chr17	4619237	4799227	-1.2823	97	-2.6608	YES	chr17	72804	18537304	-0.6050	3102	
CLL192TD	chr17	4800480	4804024	-2.1384	8	-1.8273	YES	chr17	72804	18537304	-0.6050	3102	
CLL192TD	chr17	4804779	5042231	-1.2205	113	-3.3036	YES	chr17	72804	18537304	-0.6050	3102	
CLL192TD	chr17	5044696	5388443	-0.8610	85	-4.1126	YES	chr17	72804	18537304	-0.6050	3102	
CLL192TD	chr17	5389344	6428669	-1.3372	57	-2.8142	YES	chr17	72804	18537304	-0.6050	3102	
CLL192TD	chr17	6441264	6905704	-0.9479	70	-3.6677	YES	chr17	72804	18537304	-0.6050	3102	
CLL192TD	chr17	6908528	8222580	-1.2734	764	-3.2141	YES	chr17	72804	18537304	-0.6050	3102	
CLL145TD	chr17	7186501	7256220	-0.8010	76	-2.5775	YES	chr17	7186117	7258392	-0.6833	76	
CLL192TD	chr17	8222778	8366594	-0.8242	19	-4.0862	YES	chr17	72804	18537304	-0.6050	3102	
CLL192TD	chr17	8370234	8421908	-1.1691	23	-3.6240	YES	chr17	72804	18537304	-0.6050	3102	
CLL192TD	chr17	8422173	8526211	-0.7201	20	-3.9512	YES	chr17	72804	18537304	-0.6050	3102	
CLL192TD	chr17	8633426	10222061	-1.2305	154	-3.3931	YES	chr17	72804	18537304	-0.6050	3102	
CLL192TD	chr17	10223454	10531892	-0.7020	150	-4.8723	YES	chr17	72804	18537304	-0.6050	3102	
CLL192TD	chr17	10532832	13399511	-0.9611	201	-4.0569	YES	chr17	72804	18537304	-0.6050	3102	
CLL192TD	chr17	13972853	14139839	-1.0640	10	-2.4812	YES	chr17	72804	18537304	-0.6050	3102	
CLL192TD	chr17	14683126	16219950	-0.9559	129	-3.7600	YES	chr17	72804	18537304	-0.6050	3102	
CLL192TD	chr17	16221005	16336799	-1.3820	20	-3.5389	YES	chr17	72804	18537304	-0.6050	3102	
CLL192TD	chr17	16338153	16675251	-0.8093	35	-3.2615	YES	chr17	72804	18537304	-0.6050	3102	
CLL192TD	chr17	16676766	18332957	-1.3070	336	-2.6550	YES	chr17	72804	18537304	-0.6050	3102	
CLL192TD	chr17	18481231	18498417	-0.9459	6	-2.8128	YES	chr17	72804	18537304	-0.6050	3102	
CLL192TD	chr17	19400745	21821981	0.4045	212	2.5914	YES	chr17	18752453	22028853	0.4425	292	
CLL110TD	chr17	28887107	28903008	-0.4911	7	-1.6728	NO						
CLL100TD	chr17	42461610	42463318	-1.4973	7	-1.8743	NO						
CLL023TD	chr17	44336902	45699089	0.5388	135	3.4832	YES	chr17	44165526	81027191	0.3915	4296	
CLL023TD	chr17	45730000	48246413	0.3913	369	2.9082	YES	chr17	44165526	81027191	0.3915	4296	
CLL023TD	chr17	48762032	53237094	0.6592	147	4.4453	YES	chr17	44165526	81027191	0.3915	4296	
CLL023TD	chr17	53342782	54972833	0.5296	54	3.8592	YES	chr17	44165526	81027191	0.3915	4296	
CLL023TD	chr17	54973239	56780503	0.4121	243	3.2151	YES	chr17	44165526	81027191	0.3915	4296	
CLL023TD	chr17	56783887	60744084	0.5738	458	4.0601	YES	chr17	44165526	81027191	0.3915	4296	
CLL274TD	chr17	58088453	58096254	0.8702	8	1.9898	NO						
CLL110TD	chr17	58232611	58236537	-0.9219	7	-1.8002	NO						
CLL023TD	chr17	60788558	61492841	0.5676	33	4.1265	YES	chr17	44165526	81027191	0.3915	4296	
CLL023TD	chr17	61495597	61805651	0.3578	90	2.8247	YES	chr17	44165526	81027191	0.3915	4296	
CLL023TD	chr17	61824220	61899285	0.5523	36	4.5703	YES	chr17	44165526	81027191	0.3915	4296	
CLL023TD	chr17	61901094	62121333	0.3068	85	2.8891	YES	chr17	44165526	81027191	0.3915	4296	

CLL023TD	chr17	62122193	63553910	0.4889	170	4.0546	YES	chr17	44165526	81027191	0.3915	4296	
CLL023TD	chr17	63632030	65928020	0.6218	154	4.3216	YES	chr17	44165526	81027191	0.3915	4296	
CLL023TD	chr17	65936543	66533539	0.4409	76	4.1865	YES	chr17	44165526	81027191	0.3915	4296	
CLL023TD	chr17	66535447	67516362	0.7207	204	4.4307	YES	chr17	44165526	81027191	0.3915	4296	
CLL023TD	chr17	67517155	71166771	0.4756	26	3.5217	YES	chr17	44165526	81027191	0.3915	4296	
CLL023TD	chr17	71192557	78969424	0.3138	1324	2.2168	YES	chr17	44165526	81027191	0.3915	4296	
CLL023TD	chr17	80404445	81006482	0.3677	99	2.6037	YES	chr17	44165526	81027191	0.3915	4296	
CLL008TD	chr18	47434	2795905	-0.5633	168	-2.5584	NO						SUBCLONE
CLL022TD	chr18	47434	3277211	-0.8853	235	-4.0345	YES	chr18	14116	14978275	-0.8543	826	
CLL182TD	chr18	47434	15270910	-0.3462	844	-1.7121	NO						SUBCLONE
CLL008TD	chr18	2847747	5478218	-0.5924	110	-3.0624	NO						SUBCLONE
CLL022TD	chr18	3277728	5410501	-1.1861	32	-4.5117	YES	chr18	14116	14978275	-0.8543	826	
CLL022TD	chr18	5415766	15330053	-0.8564	574	-3.5080	YES	chr18	14116	14978275	-0.8543	826	
CLL008TD	chr18	5956152	18531256	-0.5673	552	-2.5559	NO						SUBCLONE
CLL100TD	chr18	67872788	78005155	-1.0679	220	-3.6304	YES	chr18	68818906	78015254	-0.8682	218	
CLL155TD	chr18	74671557	74962477	-0.6199	16	-2.2153	NO						SUBCLONE
CLL030TD	chr19	1487761	1496569	-1.7960	6	-1.8918	NO						
CLL189TD	chr19	2339769	2344540	-1.5935	7	-1.7747	NO						
CLL191TD	chr19	37309275	37487180	-1.1080	19	-4.6655	YES	chr19	37294161	37493102	-0.9718	19	
CLL136TD	chr19	41021166	41075537	-0.8430	8	-2.0517	NO						
CLL043TD	chr19	51883675	52364144	0.3666	71	2.6405	YES	chr19	51882938	59097086	0.4277	1235	
CLL043TD	chr19	52375741	52671257	0.6200	36	5.3871	YES	chr19	51882938	59097086	0.4277	1235	
CLL043TD	chr19	52772815	53473199	0.7979	68	5.9268	YES	chr19	51882938	59097086	0.4277	1235	
CLL043TD	chr19	53507866	54139805	0.5726	61	4.7213	YES	chr19	51882938	59097086	0.4277	1235	
CLL043TD	chr19	54169885	54265580	0.7745	45	4.6577	YES	chr19	51882938	59097086	0.4277	1235	
CLL043TD	chr19	54290871	55593083	0.3644	398	2.1553	YES	chr19	51882938	59097086	0.4277	1235	
CLL043TD	chr19	55597124	55817568	0.3480	92	1.6494	YES	chr19	51882938	59097086	0.4277	1235	
CLL013TD	chr19	55604133	55610270	-1.2369	6	-1.6191	NO						
CLL043TD	chr19	55828123	59093631	0.4240	444	3.5227	YES	chr19	51882938	59097086	0.4277	1235	
CLL022TD	chr2	41527	25061688	0.5351	1033	3.5597	YES	chr2	50461	89160333	0.4951	5272	
CLL017TD	chr2	41527	30381476	0.5549	1891	3.8323	YES	chr2	16819	30432982	0.5429	1891	
CLL008TD	chr2	41527	89160356	0.4833	5258	3.2568	YES	chr2	16819	89160333	0.4635	5257	
CLL020TD	chr2	41527	89102306	0.5442	5306	3.6805	YES	chr2	16819	89126843	0.5388	5306	
CLL022TD	chr2	25062670	27760245	0.4115	580	2.7774	YES	chr2	50461	89160333	0.4951	5272	
CLL275TD	chr2	25466965	25497762	0.4522	10	1.7179	NO						
CLL022TD	chr2	27788182	32530506	0.4955	433	3.2729	YES	chr2	50461	89160333	0.4951	5272	
CLL022TD	chr2	32532136	64796694	0.5705	1774	3.8790	YES	chr2	50461	89160333	0.4951	5272	
CLL274TD	chr2	42181186	42284725	0.6794	7	1.7297	NO						
CLL274TD	chr2	45169216	45418011	0.6798	6	1.6342	NO						
CLL017TD	chr2	50883447	66798339	0.3224	765	2.3601	YES	chr2	52017346	66917238	0.3384	752	
CLL022TD	chr2	64800020	74604723	0.4794	728	3.2032	YES	chr2	50461	89160333	0.4951	5272	
CLL022TD	chr2	74605039	74762648	0.3171	111	2.5272	YES	chr2	50461	89160333	0.4951	5272	
CLL022TD	chr2	74763079	75157569	0.4746	50	2.8205	YES	chr2	50461	89160333	0.4951	5272	
CLL022TD	chr2	75186449	85051022	0.6177	175	4.1715	YES	chr2	50461	89160333	0.4951	5272	
CLL022TD	chr2	85059163	87049414	0.4835	282	3.2891	YES	chr2	50461	89160333	0.4951	5272	
CLL063TD	chr2	85628249	85661417	0.6690	6	1.6646	NO						
CLL022TD	chr2	87080104	89160691	0.4569	106	2.7180	YES	chr2	50461	89160333	0.4951	5272	
CLL023TD	chr2	89156854	89161356	-1.1652	6	-3.5646	YES	chr2	89132766	89160333	-0.8755	2	
CLL173TD	chr2	89156854	89161356	-2.5486	6	-5.9544	YES	chr2	89132766	89158640	-2.9058	1	
CLL174TD	chr2	89156854	89161356	-1.1072	6	-3.2831	YES	chr2	89132766	89158640	-0.8230	1	
CLL168TD	chr2	89156854	89196972	-5.0728	10	-7.0221	YES	chr2	89132766	89276358	-1.9190	14	
CLL272TD	chr2	89156854	89196972	-3.6236	10	-6.1602	YES	chr2	89132766	89160333	-3.9312	2	
CLL009TD	chr2	89156854	89290378	-3.2773	17	-7.0406	YES	chr2	89132766	89276358	-2.8620	14	
CLL090TD	chr2	89156854	89320097	-0.9598	25	-3.6516	YES	chr2	89132766	89158640	-2.1664	1	
CLL278TD	chr2	89156854	89327096	-4.2794	27	-6.8116	YES	chr2	89132766	89508208	-1.9548	39	
CLL048TD	chr2	89156854	89417220	-1.0894	33	-3.4909	YES	chr2	89132766	89441299	-0.7572	33	
CLL170TD	chr2	89156854	89512875	-0.9394	39	-3.3298	YES	chr2	89132766	89488503	-0.7020	38	
CLL166TD	chr2	89156854	89544238	-1.1298	44	-3.4770	YES	chr2	89126443	89480685	-0.5831	39	
CLL189TD	chr2	89156854	90249067	-0.9460	77	-3.3042	YES	chr2	89132766	89160333	-1.5694	2	
CLL013TD	chr2	89160038	89196972	-0.9390	9	-2.9641	YES	chr2	89159933	89234800	-0.9188	9	
CLL175TD	chr2	89160038	89246786	-0.9414	10	-3.6095	YES	chr2	89163662	89234800	-0.7846	4	
CLL171TD	chr2	89160038	89290378	-4.2233	16	-7.4763	YES	chr2	89159933	89203473	-2.9553	9	
CLL145TD	chr2	89160038	89309863	-0.8463	21	-3.1907	YES	chr2	89163662	89312731	-0.6859	16	
CLL186TD	chr2	89160038	89309446	-1.0574	21	-3.6263	YES	chr2	89159933	89276358	-0.8164	13	
CLL032TD	chr2	89160038	89399739	-0.9336	31	-3.1934	YES	chr2	89159933	89312731	-0.7068	22	
CLL266TD	chr2	89160038	89544964	-0.7795	44	-2.7875	YES	chr2	89159933	89539015	-0.6806	42	
CLL029TD	chr2	89160356	89196972	-2.9727	8	-5.7343	YES	chr2	89185690	89196925	-4.0093	2	
CLL184TD	chr2	89160356	89196972	-4.7055	8	-7.1251	YES	chr2	89163662	89244018	-2.6475	4	
CLL274TD	chr2	89160356	89196972	-0.8589	8	-2.5221	YES	chr2	89185102	89196925	-0.7634	3	
CLL005TD	chr2	89160356	89290378	-0.9660	15	-4.2719	YES	chr2	89129332	89480685	-0.7712	40	
CLL124TD	chr2	89160356	89290378	-0.9793	15	-3.8947	YES	chr2	89163662	89276358	-0.7008	8	
CLL192TD	chr2	89160356	89292312	-0.7631	17	-3.7334	YES	chr2	89163662	89276358	-0.6073	8	
CLL321TD	chr2	89160356	89319463	-0.9034	21	-3.5256	YES	chr2	89159933	89441299	-0.7253	32	
CLL042TD	chr2	89160356	89417220	-1.9590	32	-5.2045	YES	chr2	89163662	89430043	-1.3733	28	
CLL045TD	chr2	89160356	89417220	-0.9708	32	-3.2584	NO						IGK
CLL100TD	chr2	89160356	89475786	-4.1794	36	-6.4438	YES	chr2	89132766	89450569	-2.3855	35	
CLL144TD	chr2	89160356	89533640	-0.9031	38	-3.5036	YES	chr2	89163662	89487166	-0.7306	31	
CLL041TD	chr2	89160356	89513294	-2.1924	39	-5.1665	YES	chr2	89163662	89450569	-1.6698	30	

CLL007TD	chr2	89160356	89534308	-0.8856	41	-3.0161	YES	chr2	89163662	89430043	-0.7962	28	
CLL117TD	chr2	89160356	89568144	-0.9329	45	-3.0710	YES	chr2	89163662	89602064	-0.6157	41	
CLL051TD	chr2	89160691	89417220	-3.3980	31	-5.8378	YES	chr2	89163662	89430043	-2.3841	28	
CLL172TD	chr2	89160691	89442506	-0.7657	32	-3.0839	YES	chr2	89163662	89441299	-0.7525	27	
CLL282TD	chr2	89160691	89417220	-7.7098	32	-6.4613	YES	chr2	89163662	89312731	-3.4583	17	
CLL194TD	chr2	89160691	89513294	-5.9925	38	-6.6104	YES	chr2	89132766	89508208	-2.5227	39	
CLL052TD	chr2	89160691	89513294	-2.2623	39	-5.1414	YES	chr2	89163662	89232525	-1.9294	4	
CLL063TD	chr2	89160691	89533640	-0.7950	39	-3.0358	YES	chr2	89163662	89617070	-0.6622	41	
CLL182TD	chr2	89160691	89513294	-0.9851	39	-3.3092	YES	chr2	89450314	89508208	-0.4770	3	
CLL267TD	chr2	89160691	89544964	-0.8717	42	-2.8987	YES	chr2	89163662	89539015	-0.6202	37	
CLL082TD	chr2	89160691	89568144	-8.8327	43	-6.4135	YES	chr2	89158240	90226606	-1.7380	72	
CLL178TD	chr2	89160691	89568144	-9.5138	43	-6.4361	YES	chr2	89163662	89186090	-4.2350	2	
CLL008TD	chr2	89160691	89975667	-0.6300	47	-2.3127	YES	chr2	89163662	89312731	-0.6907	17	
CLL020TD	chr2	89160995	89196972	-6.9250	6	-7.4900	YES	chr2	89163662	89241426	-3.6293	4	
CLL279TD	chr2	89160995	89196972	-0.6769	6	-3.0212	YES	chr2	89163662	89241426	-0.7602	4	
CLL319TD	chr2	89160995	89196972	-0.9056	6	-2.9543	NO						IGK
CLL038TD	chr2	89160995	89309446	-2.2245	18	-6.0223	YES	chr2	89163662	89276358	-1.8013	8	
CLL022TD	chr2	89160995	89320097	-0.9468	21	-3.1892	YES	chr2	89163662	89312731	-0.6908	17	
CLL270TD	chr2	89160995	89327096	-1.1610	23	-3.8059	YES	chr2	89132766	89312731	-0.8242	23	
CLL040TD	chr2	89160995	89345875	-0.7401	24	-2.7909	YES	chr2	89167033	89312731	-0.2702	16	
CLL054TD	chr2	89160995	89345875	-0.9161	26	-3.1440	YES	chr2	89163662	89312731	-0.7494	17	
CLL152TD	chr2	89160995	89385101	-0.8927	27	-2.9719	YES	chr2	89132766	89312731	-0.7458	23	
CLL053TD	chr2	89160995	89417220	-1.0671	30	-3.1783	YES	chr2	89163662	89430043	-0.7718	28	
CLL136TD	chr2	89160995	89513294	-0.9438	37	-3.1657	YES	chr2	89132766	90265260	-0.5676	83	
CLL083TD	chr2	89160995	89533640	-5.1079	38	-6.3510	YES	chr2	89163662	89203473	-3.4564	4	
CLL159TD	chr2	89160995	89534308	-4.4536	39	-6.3688	YES	chr2	89158240	89539015	-1.7080	42	
CLL197TD	chr2	89160995	89534308	-0.7321	39	-2.9673	YES	chr2	89163662	89534288	-0.6896	36	
CLL006TD	chr2	89160995	89544964	-5.2305	41	-6.2867	YES	chr2	89163662	89203473	-4.0448	4	
CLL141TD	chr2	89160995	89568144	-0.9846	44	-3.0220	YES	chr2	89163662	89450569	-0.8406	31	
CLL322TD	chr2	89161356	89247203	-6.4075	7	-7.8980	YES	chr2	89163662	89203473	-3.1443	4	
CLL323TD	chr2	89161356	89290378	-0.8738	12	-3.6078	YES	chr2	89163662	89276358	-0.6652	8	
CLL277TD	chr2	89161356	89293214	-0.9857	16	-3.5029	YES	chr2	89132766	89276358	-0.8001	14	
CLL064TD	chr2	89161356	89320097	-0.8767	19	-3.2495	NO						IGK
CLL275TD	chr2	89161356	89320097	-0.9910	20	-3.6162	YES	chr2	89132766	89312731	-0.7727	23	
CLL280TD	chr2	89161356	89327096	-8.2141	22	-7.1460	YES	chr2	89132766	89312731	-2.7372	23	
CLL157TD	chr2	89161356	89385101	-8.3899	26	-6.6945	YES	chr2	89163662	89203473	-3.8841	4	
CLL188TD	chr2	89161356	89399319	-0.8845	26	-3.0600	YES	chr2	89163662	89312731	-0.8007	17	
CLL049TD	chr2	89161356	89399739	-3.5881	28	-6.0743	YES	chr2	89163662	89312731	-2.3126	17	
CLL148TD	chr2	89161356	89417220	-0.9248	29	-3.0407	NO						IGK
CLL191TD	chr2	89161356	89459703	-1.0099	33	-3.2878	YES	chr2	89132766	89475572	-0.7996	38	
CLL273TD	chr2	89161356	89512875	-1.0439	35	-2.7556	YES	chr2	89159933	89508208	-0.7447	38	
CLL181TD	chr2	89161356	89544238	-0.8486	38	-3.0251	YES	chr2	89163662	89539015	-0.7093	36	
CLL017TD	chr2	89161356	89544964	-5.9335	40	-6.4746	YES	chr2	89163662	89430043	-2.9179	27	
CLL027TD	chr2	89161356	89544964	-5.0820	40	-6.2860	YES	chr2	89163662	89312731	-2.9040	17	
CLL155TD	chr2	89161356	89568144	-1.2157	41	-3.5311	YES	chr2	89163662	89596790	-0.6940	40	
CLL146TD	chr2	89185051	89320097	-0.7593	19	-3.4230	YES	chr2	89132766	89312731	-0.6997	23	
CLL174TD	chr2	89185051	89327096	-5.9416	21	-7.0279	YES	chr2	89163662	89450569	-2.3986	30	
CLL019TD	chr2	89185051	89417220	-0.7664	28	-2.5157	NO						IGK
CLL290TD	chr2	89185051	89533640	-0.8235	36	-3.0235	YES	chr2	89163662	89534288	-0.6866	36	
CLL173TD	chr2	89185051	89567725	-0.9979	41	-3.0644	NO						IGK
CLL276TD	chr2	89246786	89345454	-1.1372	19	-3.2373	YES	chr2	89243618	89312731	-0.5947	13	
CLL272TD	chr2	89246786	89513294	-1.1561	30	-3.7656	YES	chr2	89199456	89508208	-0.9253	28	
CLL023TD	chr2	89246786	89544964	-1.1567	34	-3.4086	YES	chr2	89216786	89480685	-0.6437	28	
CLL029TD	chr2	89246786	89544964	-0.7752	34	-2.5370	YES	chr2	89198090	89430043	-0.7328	23	
CLL020TD	chr2	89246786	89544964	-0.8032	35	-2.5707	YES	chr2	89243618	89312731	-0.7356	13	
CLL184TD	chr2	89246786	89986315	-0.9825	40	-2.7978	YES	chr2	89247673	89508208	-0.7063	27	
CLL322TD	chr2	89265748	89513294	-0.7624	28	-2.7642	YES	chr2	89266299	89508208	-0.4556	24	
CLL168TD	chr2	89277955	89475786	-0.7927	26	-2.2758	YES	chr2	89312331	90265260	-0.2797	59	
CLL009TD	chr2	89291895	89339688	-0.6891	11	-2.7166	NO						IGK
CLL005TD	chr2	89291895	89345875	-3.6552	13	-5.5784	YES	chr2	89129332	89480685	-0.7712	40	
CLL171TD	chr2	89291895	89385101	-1.0970	13	-3.8686	NO						IGK
CLL038TD	chr2	89309863	89534308	-0.8304	21	-2.6062	NO						IGK
CLL022TD	chr2	89326635	90414113	0.6507	58	2.8512	YES	chr2	89427165	90253749	0.6324	43	
CLL278TD	chr2	89385101	89513294	-4.0207	12	-6.1681	YES	chr2	89132766	89508208	-1.9548	39	
CLL005TD	chr2	89385101	89568144	-0.9056	18	-2.5695	YES	chr2	89129332	89480685	-0.7712	40	
CLL174TD	chr2	89399319	89475786	-7.3360	9	-6.3936	YES	chr2	89163662	89450569	-2.3986	30	
CLL157TD	chr2	89399319	89568144	-1.3725	17	-2.7942	YES	chr2	89427166	90093173	-0.4054	26	
CLL049TD	chr2	89416800	89952847	-0.6986	16	-2.0256	YES	chr2	89427166	90093173	-0.2987	26	
CLL282TD	chr2	89442025	89544964	-0.8066	11	-3.0150	NO						IGK
CLL136TD	chr2	89533640	89568144	-1.9407	6	-3.2386	YES	chr2	89132766	90265260	-0.5676	83	
CLL194TD	chr2	89533640	89568144	-0.7773	6	-2.3608	YES	chr2	89533889	90093173	-0.6219	17	
CLL082TD	chr2	89952847	90249067	-1.1368	31	-2.4598	YES	chr2	89158240	90226606	-1.7380	72	
CLL008TD	chr2	89976153	91963931	0.4251	66	1.6177	YES	chr2	89533889	91815879	0.2988	51	
CLL273TD	chr2	90153708	90259920	-0.9064	17	-1.6899	YES	chr2	90226206	90253749	-0.8810	4	
CLL278TD	chr2	90192914	90261664	-1.2169	17	-2.3904	YES	chr2	90012137	90265260	-0.5540	29	
CLL136TD	chr2	90192914	91723047	-0.7809	22	-2.2314	YES	chr2	89132766	90265260	-0.5676	83	
CLL148TD	chr2	90211703	90259920	-0.9871	13	-2.3221	YES	chr2	90226206	90253749	-0.7617	4	
CLL146TD	chr2	90218695	90259920	-1.2370	11	-2.6152	YES	chr2	90226206	90240890	-1.0300	2	

CLL020TD	chr2	90414113	95458020	0.3657	45	2.1838	NO						
CLL022TD	chr2	91766525	95458020	0.5419	40	2.8444	NO						
CLL008TD	chr2	91968412	92118294	0.9149	12	2.9807	NO						
CLL290TD	chr2	111395518	112252405	-0.6467	52	-3.1365	YES	chr2	111391997	112490498	-0.6445	52	
CLL275TD	chr2	128397592	128412342	0.6969	9	1.7534	NO						
CLL191TD	chr2	131128755	131266247	0.6333	6	2.3401	NO						
CLL048TD	chr2	233410189	240002779	-0.6051	548	-2.9720	YES	chr2	232954062	243041505	-0.5994	961	
CLL048TD	chr2	240048039	241514412	-0.5532	68	-2.5511	YES	chr2	232954062	243041505	-0.5994	961	
CLL048TD	chr2	241888334	242122775	-0.4401	59	-2.4482	YES	chr2	232954062	243041505	-0.5994	961	
CLL048TD	chr2	242169475	243080908	-0.4830	124	-2.5187	YES	chr2	232954062	243041505	-0.5994	961	
CLL020TD	chr20	68319	17596034	-0.6355	976	-2.5639	YES	chr20	14781213	14826157	-1.3738	0	
CLL020TD	chr20	17596522	29612290	-0.4681	493	-2.0619	YES	chr20	17666114	26193955	-0.4327	471	
CLL043TD	chr20	29614140	29647090	0.5403	12	2.6689	NO						
CLL148TD	chr20	31035315	31062354	0.7721	8	1.8309	NO						
CLL191TD	chr20	60882407	60902255	0.5736	24	1.8246	NO						
CLL191TD	chr20	61950773	61960913	0.7516	13	1.7355	NO						
CLL141TD	chr21	15745911	16336974	-0.8867	25	-3.6095	YES	chr21	15711781	16958298	-0.7604	25	
CLL280TD	chr22	22385540	22724128	-0.8774	23	-3.1085	YES	chr22	22381553	22722107	-0.8807	22	
CLL168TD	chr22	22385540	22735388	-0.6581	26	-2.4848	YES	chr22	22385947	22748867	-0.6599	25	
CLL182TD	chr22	22556219	22657565	-1.8207	7	-3.2332	YES	chr22	22551038	23241689	-0.8108	70	
CLL182TD	chr22	22658390	23241756	-1.0228	64	-3.6058	YES	chr22	22551038	23241689	-0.8108	70	
CLL278TD	chr22	22724128	22975934	-0.8894	22	-3.8973	YES	chr22	22713221	23244777	-0.7171	51	
CLL280TD	chr22	22730538	23241756	-9.1392	46	-7.9127	YES	chr22	22724404	23247059	-2.8660	47	
CLL189TD	chr22	22735388	23114750	-0.4651	36	-2.2611	YES	chr22	22825827	23247059	-0.5367	43	
CLL136TD	chr22	22735388	23223249	-0.8792	42	-4.0428	YES	chr22	22733651	23234829	-0.6759	42	
CLL045TD	chr22	22735388	23235817	-0.9112	44	-3.6092	YES	chr22	22736444	23234829	-0.7498	42	
CLL090TD	chr22	22735388	23165257	-1.0235	44	-3.6300	YES	chr22	22736444	23247059	-0.8489	50	
CLL064TD	chr22	22735388	23247126	-1.0508	49	-3.6034	YES	chr22	22736444	23247059	-0.8703	47	
CLL168TD	chr22	22749500	23247126	-2.6228	48	-6.2688	YES	chr22	22749794	23247059	-1.8130	47	
CLL173TD	chr22	22782035	22906026	-9.0236	12	-10.2824	YES	chr22	22765216	23247059	-2.4999	45	
CLL173TD	chr22	22930830	23247126	-6.7858	34	-6.3368	YES	chr22	22765216	23247059	-2.4999	45	
CLL042TD	chr22	22984803	23264746	-0.3468	34	-2.0362	YES	chr22	23026882	23246029	-0.3974	23	
CLL278TD	chr22	22989137	23247126	-0.8146	27	-2.5712	YES	chr22	22713221	23244777	-0.7171	51	
CLL020TD	chr22	23046981	23241756	-0.9664	20	-3.1815	YES	chr22	23041645	23241689	-0.8852	19	
CLL019TD	chr22	23055479	23241756	-0.6726	19	-3.1363	YES	chr22	23056362	23240427	-0.7890	17	
CLL006TD	chr22	23063315	23247126	-5.7736	19	-6.7538	YES	chr22	23056362	23247059	-3.0147	18	
CLL009TD	chr22	23063315	23247126	-0.8632	19	-2.7997	YES	chr22	23056362	23247059	-0.8457	18	
CLL082TD	chr22	23077248	23241756	-1.0199	17	-3.8283	YES	chr22	23064017	23239061	-0.7084	16	
CLL272TD	chr22	23077248	23241756	-0.9569	17	-3.3931	YES	chr22	23085408	23241689	-0.7889	15	
CLL029TD	chr22	23101171	23223249	-7.0442	12	-6.7989	YES	chr22	23094250	23234829	-3.2471	12	
CLL018TD	chr22	23101171	23237534	-0.9201	14	-3.0871	YES	chr22	23103564	23247059	-0.8098	13	
CLL146TD	chr22	23114750	23235817	-6.8565	11	-7.2343	YES	chr22	23108423	23234829	-2.4126	10	
CLL041TD	chr22	23114750	23241756	-2.5279	13	-5.7025	YES	chr22	23103564	23244777	-1.7194	13	
CLL189TD	chr22	23134963	23247126	-1.2274	13	-3.7914	YES	chr22	22825827	23247059	-0.5367	43	
CLL276TD	chr22	23135163	23247126	-0.8349	12	-2.6336	YES	chr22	23138710	23247059	-0.6702	10	
CLL032TD	chr22	23135163	23256401	-0.6361	14	-2.8456	YES	chr22	23138710	23258429	-0.6904	13	
CLL178TD	chr22	23154453	23223249	-10.0000	7	-6.8542	YES	chr22	23094250	23241689	-2.7407	14	
CLL194TD	chr22	23165454	23241756	-1.0530	7	-3.7821	YES	chr22	23165575	23241689	-0.7516	6	
CLL090TD	chr22	23165454	23247126	-4.2365	8	-5.5748	YES	chr22	22736444	23247059	-0.8489	50	
CLL173TD	chr22	29611477	29664292	-0.9553	14	-1.7096	NO						
CLL082TD	chr22	29630096	29654719	-1.1664	6	-1.6548	NO						
CLL030TD	chr22	39224254	39353650	-1.6392	7	-1.8007	NO						
CLL274TD	chr22	46318645	46508605	0.9931	11	1.6096	NO						
CLL064TD	chr22	47243690	51237147	-1.1043	384	-2.4403	YES	chr22	47218653	51224402	-0.9280	383	
CLL023TD	chr3	361444	4403822	-0.7572	110	-3.5738	YES	chr3	61875	5848545	-0.7837	205	
CLL023TD	chr3	4417953	6903034	-0.9197	96	-4.8966	YES	chr3	61875	5848545	-0.7837	205	
CLL141TD	chr3	47030690	48921373	-1.1265	499	-3.4846	YES	chr3	47031601	48917211	-1.0435	496	
CLL174TD	chr3	47437579	47476412	-1.0411	31	-2.9290	YES	chr3	47416540	47522517	-0.8446	32	
CLL174TD	chr3	47852127	48450938	-0.9519	92	-4.1059	YES	chr3	47851098	49978951	-0.8177	692	
CLL174TD	chr3	48451249	48462564	-1.3742	22	-2.6436	YES	chr3	47851098	49978951	-0.8177	692	
CLL174TD	chr3	48463073	49977893	-1.0145	578	-3.1083	YES	chr3	47851098	49978951	-0.8177	692	
CLL275TD	chr3	51467454	51624541	-0.4248	16	-1.7265	NO						
CLL048TD	chr3	118945621	122591232	0.3961	401	3.3587	YES	chr3	118985758	197840480	0.4404	4761	
CLL048TD	chr3	122598047	122634528	0.9536	7	1.9689	YES	chr3	118985758	197840480	0.4404	4761	
CLL100TD	chr3	122630721	122642411	-0.8681	8	-1.6287	NO						
CLL048TD	chr3	122642411	125731374	0.5052	293	2.5767	YES	chr3	118985758	197840480	0.4404	4761	
CLL274TD	chr3	123014846	123036825	1.0001	7	1.7602	NO						
CLL100TD	chr3	123213715	125735525	0.4992	245	3.5786	YES	chr3	122827975	197840480	0.5264	4435	
CLL100TD	chr3	126326724	126682088	0.5648	25	4.2483	YES	chr3	122827975	197840480	0.5264	4435	
CLL100TD	chr3	127337815	129902316	0.3148	280	2.5127	YES	chr3	122827975	197840480	0.5264	4435	
CLL048TD	chr3	127395717	129262015	0.4791	200	2.4662	YES	chr3	118985758	197840480	0.4404	4761	
CLL048TD	chr3	129269529	129695836	0.8263	35	1.6384	YES	chr3	118985758	197840480	0.4404	4761	
CLL189TD	chr3	129284674	129302400	-1.0279	11	-1.8903	NO						
CLL048TD	chr3	129740488	133553361	0.3691	352	3.6268	YES	chr3	118985758	197840480	0.4404	4761	
CLL100TD	chr3	130087997	131068360	0.7251	114	5.3029	YES	chr3	122827975	197840480	0.5264	4435	
CLL100TD	chr3	131101945	132437792	0.7387	154	4.7331	YES	chr3	122827975	197840480	0.5264	4435	
CLL100TD	chr3	132438491	138213827	0.5101	328	3.9642	YES	chr3	122827975	197840480	0.5264	4435	
CLL048TD	chr3	133556966	133698243	0.7874	20	2.2541	YES	chr3	118985758	197840480	0.4404	4761	

CLL048TD	chr3	133876932	139180922	0.4395	315	3.1558	YES	chr3	118985758	197840480	0.4404	4761	
CLL110TD	chr3	138213827	138347883	0.3503	21	1.6341	NO						
CLL100TD	chr3	138216852	138477949	0.7059	45	5.1744	YES	chr3	122827975	197840480	0.5264	4435	
CLL100TD	chr3	139062804	150419876	0.6778	563	4.5470	YES	chr3	122827975	197840480	0.5264	4435	
CLL048TD	chr3	139257561	183776174	0.3933	1830	3.5552	YES	chr3	118985758	197840480	0.4404	4761	
CLL082TD	chr3	143185835	143236876	-0.8840	6	-3.7155	YES	chr3	143106361	143252946	-0.8052	6	
CLL100TD	chr3	150588782	156876649	0.6688	274	4.9933	YES	chr3	122827975	197840480	0.5264	4435	
CLL100TD	chr3	156978860	183059312	0.6781	932	4.8166	YES	chr3	122827975	197840480	0.5264	4435	
CLL100TD	chr3	183097021	183857816	0.4410	124	3.6058	YES	chr3	122827975	197840480	0.5264	4435	
CLL048TD	chr3	183777205	184070039	0.6049	177	2.0672	YES	chr3	118985758	197840480	0.4404	4761	
CLL189TD	chr3	184099493	184296137	-0.4372	25	-1.6806	NO						
CLL100TD	chr3	184428553	185183472	0.6864	72	5.2808	YES	chr3	122827975	197840480	0.5264	4435	
CLL048TD	chr3	184429154	195485943	0.4318	635	3.1223	YES	chr3	118985758	197840480	0.4404	4761	
CLL100TD	chr3	185184562	188425967	0.4814	238	3.6273	YES	chr3	122827975	197840480	0.5264	4435	
CLL100TD	chr3	188464219	193677537	0.6887	205	4.5149	YES	chr3	122827975	197840480	0.5264	4435	
CLL100TD	chr3	194124710	194309252	0.6866	32	4.7116	YES	chr3	122827975	197840480	0.5264	4435	
CLL100TD	chr3	194346582	194390650	0.6598	15	4.6293	YES	chr3	122827975	197840480	0.5264	4435	
CLL100TD	chr3	194947391	195426162	0.6686	47	3.6560	YES	chr3	122827975	197840480	0.5264	4435	
CLL100TD	chr3	195690188	195803892	0.5800	27	3.1515	YES	chr3	122827975	197840480	0.5264	4435	
CLL048TD	chr3	195778753	197894524	0.4102	255	3.0118	YES	chr3	118985758	197840480	0.4404	4761	
CLL100TD	chr3	195937427	196509460	0.4630	69	4.0153	YES	chr3	122827975	197840480	0.5264	4435	
CLL064TD	chr3	196528787	196554015	-0.7817	12	-1.7148	NO						
CLL100TD	chr3	196612028	196666092	0.5543	13	4.7394	YES	chr3	122827975	197840480	0.5264	4435	
CLL100TD	chr3	196771486	197894524	0.5659	130	4.0686	YES	chr3	122827975	197840480	0.5264	4435	
CLL267TD	chr4	420216	514821	-0.3361	9	-2.2863	YES	chr4	421075	479412	-0.7301	2	
CLL009TD	chr4	171525921	190882911	-0.8190	562	-3.7661	YES	chr4	171423540	190916819	-0.7784	566	
CLL030TD	chr5	1815937	2748537	-0.8752	6	-1.6465	NO						
CLL178TD	chr6	395830	32427550	0.4234	2299	2.8874	YES	chr6	387408	50073545	0.4160	4414	
CLL178TD	chr6	32548457	32609030	0.3704	6	2.0682	YES	chr6	387408	50073545	0.4160	4414	
CLL178TD	chr6	32709201	32796601	0.4491	18	3.3952	YES	chr6	387408	50073545	0.4160	4414	
CLL178TD	chr6	32797124	50681696	0.5202	2073	3.2795	YES	chr6	387408	50073545	0.4160	4414	
CLL191TD	chr6	33084685	33138580	0.4796	23	1.6027	NO						
CLL275TD	chr6	41533450	41558949	0.5164	11	1.7043	NO						
CLL049TD	chr6	79911333	90305623	-0.8027	471	-3.7545	YES	chr6	79892342	120465184	-0.7504	1725	
CLL049TD	chr6	90321979	119669970	-0.8049	1252	-3.7773	YES	chr6	79892342	120465184	-0.7504	1725	
CLL186TD	chr7	933354	940151	0.8525	8	2.1271	NO						
CLL019TD	chr7	40085409	40133673	-0.9796	10	-4.1262	YES	chr7	40062122	40144404	-0.8148	10	
CLL274TD	chr7	40899876	42065754	0.4098	12	1.7995	NO						
CLL189TD	chr7	100159791	100175234	-0.6941	15	-1.8074	NO						
CLL178TD	chr7	114563472	133580299	-0.9634	981	-3.7746	YES	chr7	114334126	133589746	-0.8731	981	
CLL041TD	chr7	142364197	142471717	-0.5067	16	-2.5878	NO						
CLL017TD	chr7	142364382	142471717	-1.0837	15	-3.0099	NO						
CLL030TD	chr7	150773805	150776527	-0.9186	7	-1.6947	NO						
CLL290TD	chr8	142120	7056066	-0.7127	230	-3.3682	YES	chr8	161272	29030957	-0.7051	1532	
CLL043TD	chr8	142120	9634112	-0.8545	303	-3.3608	YES	chr8	176293	41305627	-0.6845	2125	
CLL282TD	chr8	163525	7005679	-0.9576	227	-3.9727	YES	chr8	176654	34630057	-0.8628	1705	
CLL290TD	chr8	7154524	11892002	-0.7260	179	-2.8024	YES	chr8	161272	29030957	-0.7051	1532	
CLL282TD	chr8	8090295	11952657	-0.9943	157	-3.7174	YES	chr8	176654	34630057	-0.8628	1705	
CLL043TD	chr8	9945493	11052602	-0.8806	38	-3.2094	YES	chr8	176293	41305627	-0.6845	2125	
CLL043TD	chr8	11152696	11660330	-1.0237	44	-3.3471	YES	chr8	176293	41305627	-0.6845	2125	
CLL043TD	chr8	11666261	12008379	-0.5529	34	-2.1287	YES	chr8	176293	41305627	-0.6845	2125	
CLL282TD	chr8	12040162	12250700	-4.9347	9	-1.9026	YES	chr8	176654	34630057	-0.8628	1705	
CLL290TD	chr8	12215407	29024875	-0.7410	1120	-3.2616	YES	chr8	161272	29030957	-0.7051	1532	
CLL043TD	chr8	12272529	39182704	-0.7961	1607	-3.4472	YES	chr8	176293	41305627	-0.6845	2125	
CLL282TD	chr8	12282445	21883070	-0.9443	367	-4.3620	YES	chr8	176654	34630057	-0.8628	1705	
CLL282TD	chr8	21890627	22059258	-1.1987	87	-2.7754	YES	chr8	176654	34630057	-0.8628	1705	
CLL191TD	chr8	21955519	21979076	0.5146	21	1.7883	NO						
CLL282TD	chr8	22064280	34180476	-0.9702	826	-3.9682	YES	chr8	176654	34630057	-0.8628	1705	
CLL274TD	chr8	28608158	28635287	0.5715	6	1.6421	NO						
CLL282TD	chr8	35383169	43415755	0.4476	638	3.1944	YES	chr8	34638882	43388433	0.4766	637	
CLL043TD	chr8	39416134	41164188	-0.6862	75	-3.2870	YES	chr8	176293	41305627	-0.6845	2125	
CLL282TD	chr8	47886143	56015292	-0.9481	277	-4.5790	YES	chr8	47777490	56161593	-0.8713	277	
CLL178TD	chr8	56879244	67062402	0.4892	261	3.5577	YES	chr8	56882676	86550838	0.5108	930	
CLL064TD	chr8	59515736	67341285	0.5497	191	4.2217	YES	chr8	59529447	144292596	0.5558	3155	
CLL178TD	chr8	67064488	86392838	0.5811	671	4.1582	YES	chr8	56882676	86550838	0.5108	930	
CLL064TD	chr8	67352355	68123663	0.4187	123	3.2839	YES	chr8	59529447	144292596	0.5558	3155	
CLL064TD	chr8	68128800	95412458	0.5539	843	4.0851	YES	chr8	59529447	144292596	0.5558	3155	
CLL290TD	chr8	92972427	97172447	0.4605	220	3.4571	YES	chr8	92935342	146239365	0.4850	2470	
CLL064TD	chr8	95416265	100147772	0.4415	268	3.5017	YES	chr8	59529447	144292596	0.5558	3155	
CLL290TD	chr8	97343228	103326936	0.4646	346	3.4300	YES	chr8	92935342	146239365	0.4850	2470	
CLL064TD	chr8	100148876	144886210	0.5431	1826	3.7659	YES	chr8	59529447	144292596	0.5558	3155	
CLL290TD	chr8	103335510	144459527	0.5322	1515	3.4928	YES	chr8	92935342	146239365	0.4850	2470	
CLL145TD	chr8	110283176	143559513	0.5518	1217	4.0530	YES	chr8	110274049	146294242	0.4730	1670	
CLL145TD	chr8	143560645	144652614	0.7346	112	2.4159	YES	chr8	110274049	146294242	0.4730	1670	
CLL274TD	chr8	143603280	143624721	1.6797	6	1.7961	NO						
CLL290TD	chr8	144460417	145001106	0.4687	101	1.7807	YES	chr8	92935342	146239365	0.4850	2470	
CLL145TD	chr8	144654158	145057016	0.5021	95	1.9805	YES	chr8	110274049	146294242	0.4730	1670	
CLL064TD	chr8	144889662	146277947	0.3392	310	1.8157	NO						SUBCLONE

CLL290TD	chr8	145006781	145113376	0.6070	43	2.1107	YES	chr8	92935342	146239365	0.4850	2470	
CLL145TD	chr8	145058163	146279336	0.6865	246	2.7676	YES	chr8	110274049	146294242	0.4730	1670	
CLL290TD	chr8	145134798	145542116	0.4294	60	1.9806	YES	chr8	92935342	146239365	0.4850	2470	
CLL290TD	chr8	145544967	146277947	0.5117	154	2.1724	YES	chr8	92935342	146239365	0.4850	2470	
CLL266TD	chr9	14750	116709	-0.3767	6	1.8259	NO						
CLL155TD	chr9	14806631	15019428	-0.3135	19	-2.4373	NO						SUBCLONE
CLL155TD	chr9	20294882	26116054	-0.3593	115	-1.9117	NO						SUBCLONE
CLL110TD	chr9	33465663	33472186	-0.4934	15	-1.9860	NO						
CLL083TD	chr9	70484362	75906141	-0.7681	278	-3.4753	YES	chr9	70984281	77075175	-0.7101	274	
CLL110TD	chr9	90498873	90589233	-0.4764	17	-1.9858	NO						
CLL186TD	chr9	95147866	95178890	-0.8864	9	-4.2291	YES	chr9	95142442	95204491	-0.8971	9	
CLL274TD	chr9	130658505	130687342	1.1617	7	1.9659	NO						
CLL191TD	chr9	139752059	139754306	1.0479	6	2.0412	NO						
