# Supplementary figures and images for: Estimation of Copy Number Alterations from Exome Sequencing Data
Source: PLoS One. 2012 Dec 19;7(12):e51422. doi: 10.1371/journal.pone.0051422 (PMC3526607; doi:10.1371/journal.pone.0051422)

**Figure S1.** Comparison of the number of exons involved in CNAs detected by exome2cnv and aCGH.

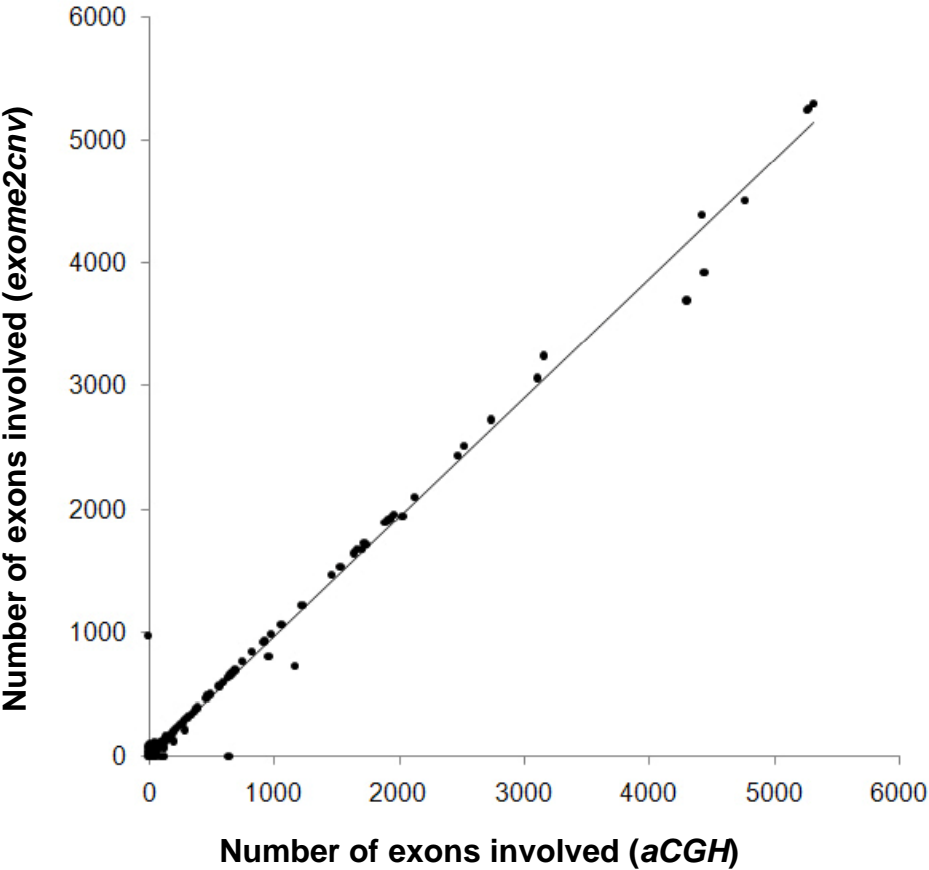

Supplement: Figure S1 — Comparison of the number of exons involved in CNAs detected by exome2cnv and aCGH. (PDF) [file pone.0051422.s003.pdf]
