# Supplementary material for: Estimation of Copy Number Alterations from Exome Sequencing Data
Source: PLoS One. 2012 Dec 19;7(12):e51422. doi: 10.1371/journal.pone.0051422 (PMC3526607; doi:10.1371/journal.pone.0051422)

Figure S2. aCGH data showing two cases (A and B) with a deletion affecting the short arm of chromosome 18 which was detected by exome2cnv but not by aCGH because it is present in a subpopulation of tumor cells. For comparison, a case (C) with a deletion affecting all tumor cells and detected by both methods is shown.

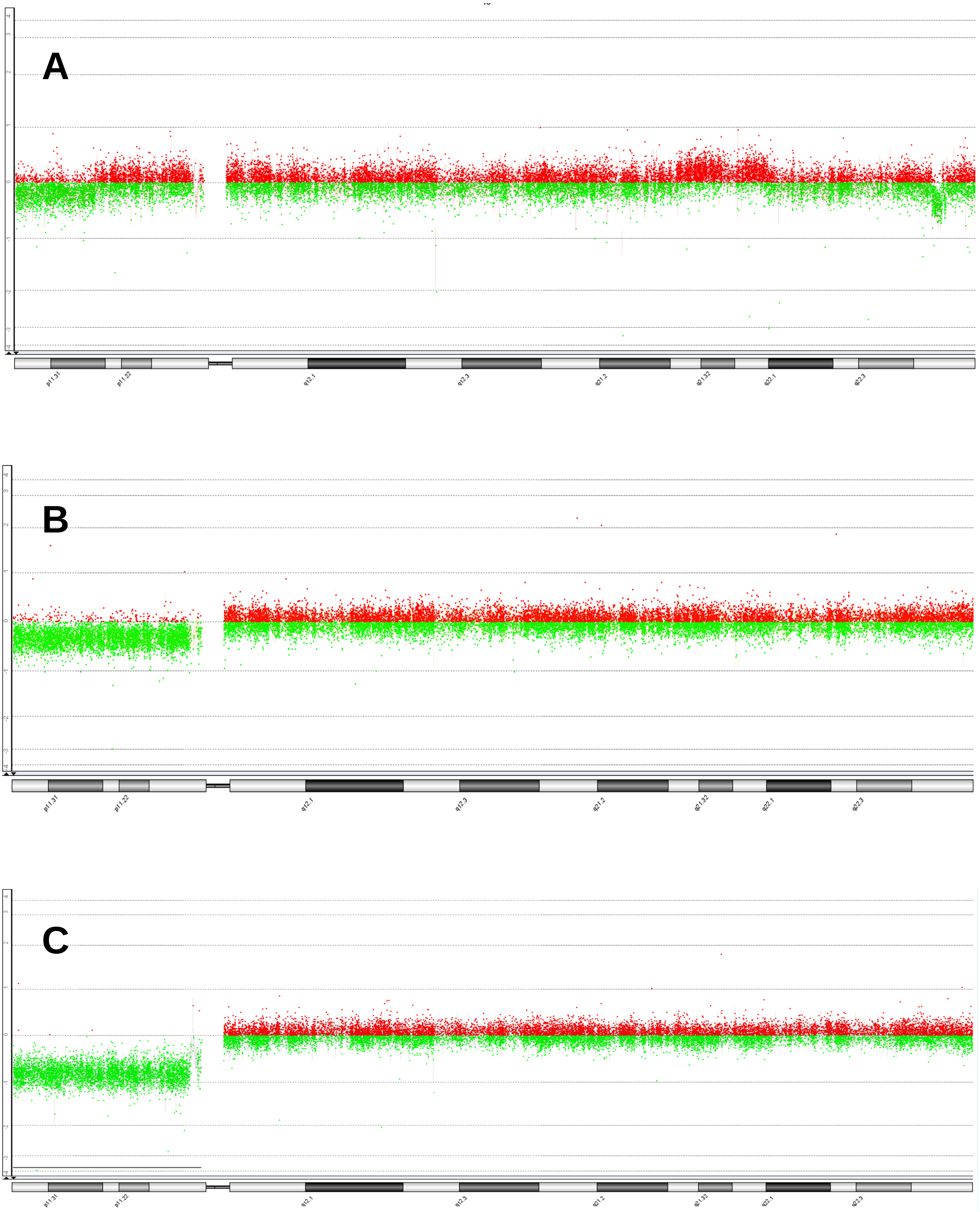

Supplement: Figure S2 — aCGH data showing two cases (A and B) with a deletion affecting the short arm of chromosome 18 which was detected by exome2cnv but not by aCGH because it is present in a subpopulation of tumor cells. For comparison, a case (C) with a deletion affecting all tumor cells and detected by both methods is shown. (PDF) [file pone.0051422.s004.pdf]
